# High Expression of Pseudogene PTTG3P Indicates a Poor Prognosis in Human Breast Cancer

**DOI:** 10.1016/j.omto.2019.03.006

**Published:** 2019-03-27

**Authors:** Weiyang Lou, Bisha Ding, Weimin Fan

**Affiliations:** 1 Program of Innovative Cancer Therapeutics, Division of Hepatobiliary and Pancreatic Surgery, Department of Surgery, First Affiliated Hospital, College of Medicine, Zhejiang University, Hangzhou 310003, China; 2 Key Laboratory of Organ Transplantation, Zhejiang Province, Hangzhou 310003, China; 3 Key Laboratory of Combined Multi-organ Transplantation, Ministry of Public Health, Hangzhou 310000, China; 4 Department of Pathology and Laboratory Medicine, Medical University of South Carolina, Charleston, SC 29425, USA

**Keywords:** breast cancer, pseudogene, pituitary tumor-transforming 3 pseudogene, PTTG3P, prognosis, bioinformatic analysis

## Abstract

Pseudogenes play pivotal roles in tumorigenesis. Previous studies have suggested that pituitary tumor-transforming 3, pseudogene (PTTG3P), serves as an oncogene in human cancers. However, its expression pattern, biological function**,** and underlying mechanism in breast cancer remain unknown. In this study, we demonstrated an elevated expression of PTTG3P in breast cancer and discovered that PTTG3P expression correlated negatively with estrogen receptor (ER) and progesterone receptor (PR) status, but linked positively to basal-like status, triple-negative breast cancer status, Nottingham prognostic index (NPI)**,** and Scarff-Bloom-Richardson grade. High expression of PTTG3P was also found to be associated with a poor prognosis of breast cancer. To explore the potential mechanisms of PTTG3P, a PTTG3P-microRNA (miRNA)-mRNA regulatory network was established. Co-expressed genes of PTTG3P were also obtained. Enrichment analysis for these co-expressed genes revealed that they were significantly enriched in mitotic nuclear division and cell cycle. Subsequent research on mechanism of PTTG3P indicated that its expression correlated positively with PTTG1 expression. However, no significant expression correlation between PTTG3P and PTTG2 was observed. Taken together, our findings suggest that increased expression of pseudogene PTTG3P may be used as a promising prognostic biomarker and novel therapeutic target for breast cancer.

## Introduction

Breast cancer is one of the most common malignancies and remains a leading cause of cancer-related deaths in the female worldwide.^1^ The incidence of breast cancer is gradually increasing in most countries, including the United States.^2^ Despite the huge improvements in screening, diagnosis, and treatment of breast cancer that have been achieved, the patient’s prognosis is still dismal. Thus, it is extremely meaningful and also urgent to search and develop more sensitive and specific prognostic biomarkers for patients with breast cancer.

Pseudogenes, a class of non-coding RNAs and segments of DNA that are related to real functional genes, are traditionally recognized as “junk genes” or “genomic fossils” because they have lost functionality.^3^ Based on the formation mechanism, pseudogenes are generally classified into three subtypes: unitary pseudogenes, duplicated pseudogenes, and processed pseudogenes.^4^ Recent studies have reported that pseudogenes play important roles in regulating parental genes by functioning as antisense RNAs, small-interference RNAs (siRNAs), or endogenous competitors for microRNA (miRNA), RNA-binding protein, or translational machinery.^4^ Pseudogenes are frequently dysregulated in multiple human cancers, and the dysregulation results in cancer onset and progression.^5^, ^6^, ^7^

Pituitary tumor-transforming 3, pseudogene (PTTG3P), is a processed pseudogene that shows high homology to its family members, pituitary tumor-transforming 1 (PTTG1) and -2 (PTTG2).^8^ Numerous investigations have shown that PTTG1 and -2 serve as two key oncogenes in a variety of human cancers, including breast cancer,^9^, ^10^ lung cancer,^11^ pituitary adenoma,^12^ and esophageal squamous cell cancer.^13^ However, there are only a few reports regarding the expression and roles of PTTG3P in cancer. One study, conducted by Weiwei Weng et al., ^14^ revealed that PTTG3P promotes proliferation and invasion and is an indicator of poor prognosis in gastric cancer. Huang et al.^15^ found that PTTG3P enhances hepatocellular carcinoma (HCC) growth and metastasis via upregulating PTTG1 and activating PI3K/AKT signaling. Notably, the expression, function, and corresponding regulatory mechanisms of PTTG3P in breast cancer remain unclear and need to be further elucidated.

Herein, we first assessed the expression pattern of PTTG3P in human breast cancer by data mining and experimental validation. Then, the prognostic significance of PTTG3P expression in breast cancer was also analyzed. Finally, we explored several potential mechanisms of PTTG3P in breast cancer, including construction of the pseudogene PTTG3P-miRNA-mRNA network, analysis of co-expressed genes, and assessment of its correlation with parental genes PTTG1 and -2. These findings facilitate understanding of PTTG3P’s roles and mechanisms in human breast cancer.

## Results

### Pseudogene PTTG3P Expression in Human Breast Cancer

**Figure 1:**
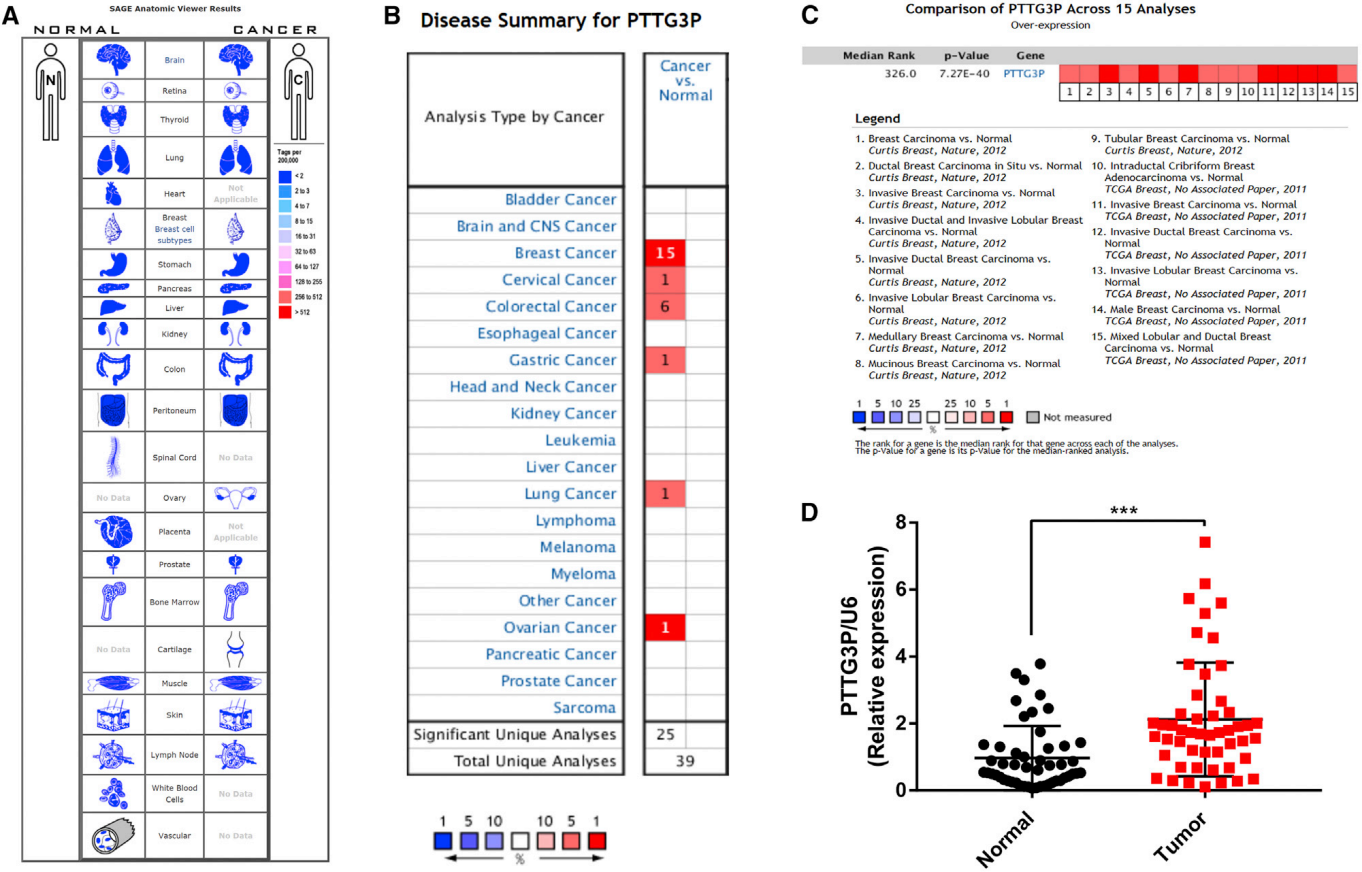
Expression Pattern of PTTG3P in Human Breast Cancer (A) Expression profile of PTTG3P determined by the SAGE Digital Gene Expression Displayer; (B) expression of PTTG3P in different types of cancers by Oncomine analysis of cancer versus normal samples; (C) expression of PTTG3P in breast cancer by Oncomine meta-analysis; and (D) expression of PTTG3P in breast cancer samples compared with matched normal samples. Error bars represent SD. ***p < 0.001.

The expression profile of pseudogene PTTG3P in human breast cancer was first determined by using the SAGE (Serial Analysis of Gene Expression) Digital Gene Expression Display. As shown in Figure 1A, pseudogene PTTG3P expression was not significantly dysregulated in all cancer tissues when compared with corresponding normal tissues, including breast cancer. Next, Oncomine analysis of cancer versus normal samples was used to analyze the expression profile of pseudogene PTTG3P in human breast cancer (Figure 1B). Oncomine meta-analysis was further employed to assess the comprehensive median expression of pseudogene PTTG3P across 15 analyses (Figure 1C). As presented in Table 1, expression of pseudogene PTTG3P was significantly higher in medullary breast carcinoma, invasive breast carcinoma, invasive lobular breast carcinoma, invasive ductal breast carcinoma, mucinous breast carcinoma, ductal breast carcinoma *in situ*, male breast carcinoma, intraductal cribriform breast adenocarcinoma, and mixed lobular and ductal breast carcinoma. For further validation, we also detected the expression of pseudogene PTTG3P in clinical breast cancer samples. As shown in Figure 1D, pseudogene PTTG3P expression in breast cancer tissues was markedly higher than that in normal tissues. All evidence taken together, by combining bioinformatics analysis and experimental validation, we demonstrated that PTTG3P was significantly overexpressed in human breast cancer.

**Table 1:** Significant Changes of PTTG3P Expression in the Transcriptional Level between Different Types of Breast Cancer and Normal Tissues (Oncomine Database)

Subtype of Breast Cancer	p Value	Fold Change	t Test	Rank	Sample Count	Reporter ID
Medullary breast carcinoma	1.52E−19	6.164	17.706	1%	176	ILMN_2049021
Invasive breast carcinoma	3.63E−9	3.270	9.087	1%	165	ILMN_2049021
Invasive ductal breast carcinoma	6.87E−111	3.429	39.869	1%	1700	ILMN_2049021
Invasive lobular breast carcinoma	7.27E−40	2.371	16.045	2%	292	ILMN_2049021
Invasive ductal and invasive lobular breast carcinoma	3.69E−29	2.791	14.788	2%	234	ILMN_2049021
Mucinous breast carcinoma	1.77E−15	2.462	10.742	3%	190	ILMN_2049021
Ductal breast carcinoma *in Situ*	2.13E−4	2.508	5.291	4%	154	ILMN_2049021
Breast carcinoma	3.76E−5	2.915	5.595	5%	158	ILMN_2049021
Tubular breast carcinoma	7.17E−20	2.325	11.684	5%	211	ILMN_2049021
Male breast carcinoma	1.14E−17	2.467	12.903	1%	64	A_23_P60016
Invasive ductal breast carcinoma	1.35E−45	4.791	24.131	1%	450	A_23_P60016
Invasive lobular breast carcinoma	1.05E−16	3.327	11.193	1%	97	A_23_P60016
Invasive breast carcinoma	2.86E−27	3.547	13.653	1%	137	A_23_P60016
Intraductal cribriform breast adenocarcinoma	6.92E−6	3.115	9.941	3%	64	A_23_P60016
Mixed lobular and ductal breast carcinoma	7.72E−5	2.553	6.204	4%	68	A_23_P60016

**Figure 2:**
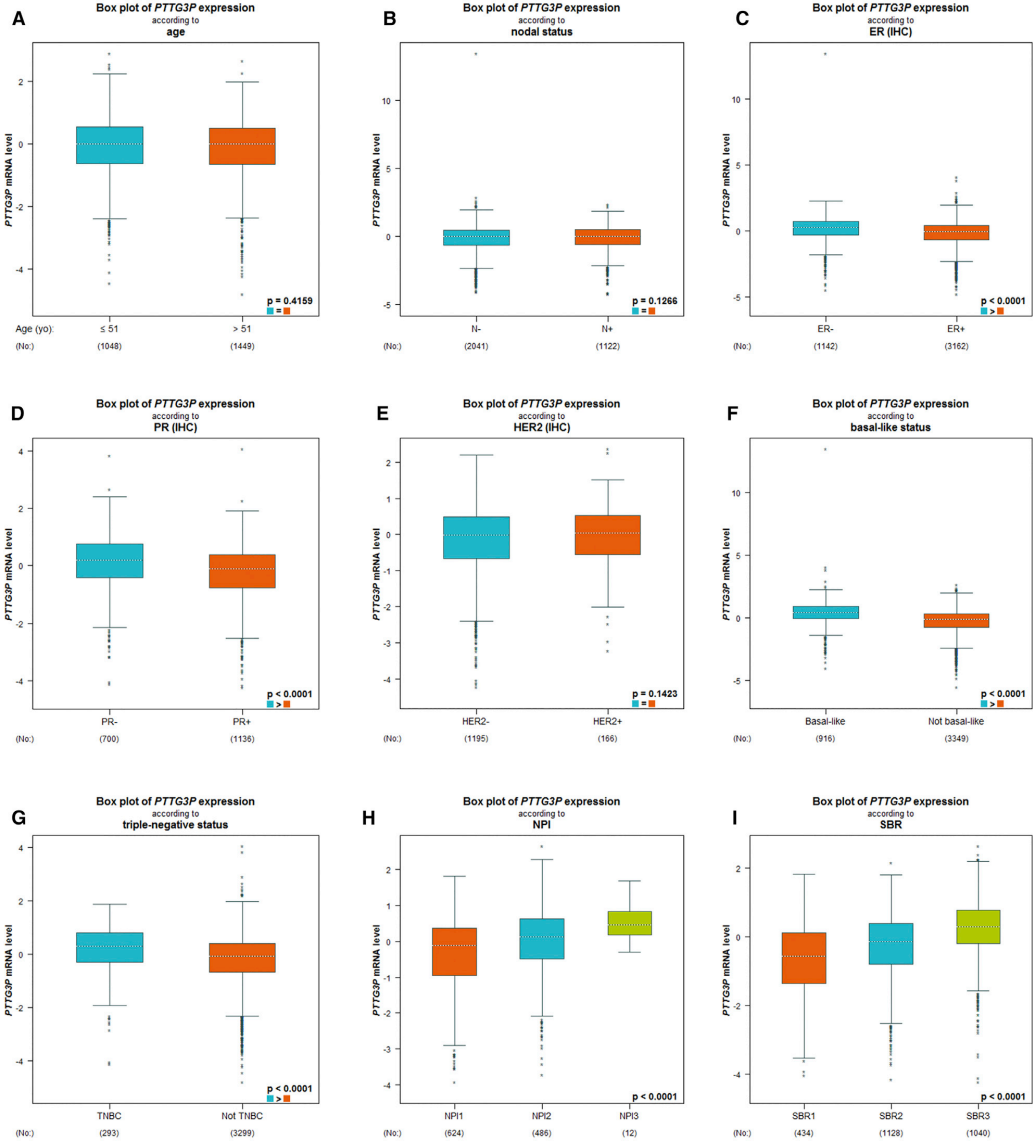
Relationship between Expression of PTTG3P and Various Clinicopathological Parameters of Breast Cancer Determined by the bc-GenExMiner Database (A) Boxplot of PTTG3P expression according to age; (B) boxplot of PTTG3P expression according to nodal status; (C) boxplot of PTTG3P expression according to ER; (D) boxplot of PTTG3P expression according to PR; (E) boxplot of PTTG3P expression according to HER2; (F) boxplot of PTTG3P expression according to basal-like status; (G) boxplot of PTTG3P expression according to triple-negative status; (H) boxplot of PTTG3P expression according to NPI; and (I) boxplot of PTTG3P expression according to SBR.

We further investigated the associations between pseudogene PTTG3P expression and different clinicopathological characteristics in patients with breast cancer, by using bc-GenExMiner (Figure 2; Table S1). Analytic results suggested that there are no significant differences between the ≤51- and >51-year group (p = 0.4159). Nodal and HER2 status also showed no significant differences between the positive and negative groups (nodal status: p = 0.1266; HER2 status: p = 0.1423). Estrogen receptor (ER) and progesterone receptor (PR) status were discovered to be negatively linked to pseudogene PTTG3P expression (ER status: p < 0.0001; PR status: p < 0.0001). Pseudogene PTTG3P was significantly upregulated in basal-like or triple-negative breast cancer when compared with their counterparts (basal-like status: p < 0.0001; triple-negative status: p < 0.0001). The Nottingham prognostic index (NPI) is often used to determine prognosis after surgery for breast cancer. A higher NPI correlated significantly with higher expression of pseudogene PTTG3P expression. Similarly, a more advanced Scarff-Bloom-Richardson (SBR) grade was associated with higher expression of pseudogene PTTG3P expression. To summarize, these results demonstrate that pseudogene PTTG3P expression may play an unfavorable role in patients with breast cancer.

### Prognostic Significance of the Pseudogene PTTG3P in Human Breast Cancer

**Figure 3:**
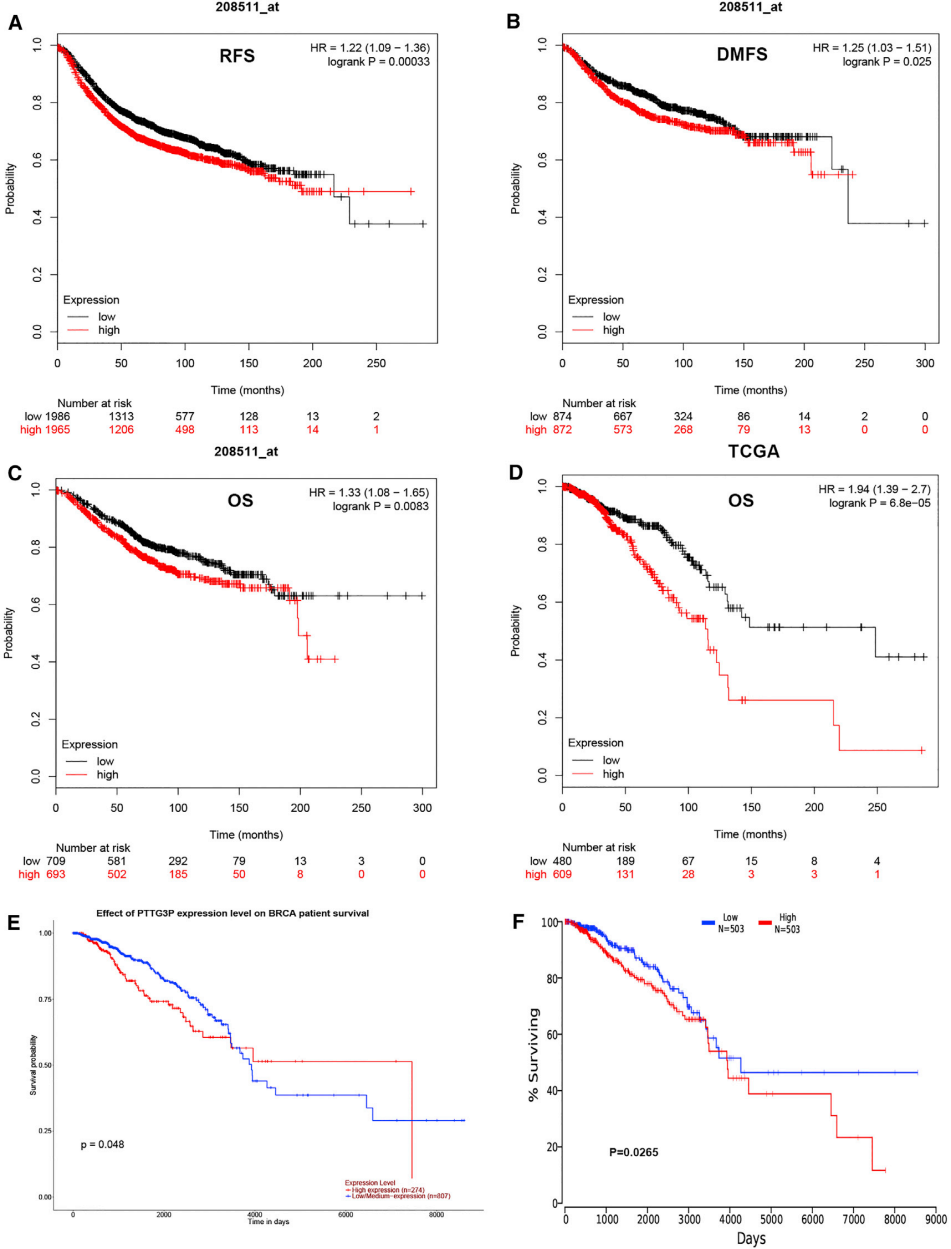
Prognostic Value of PTTG3P in Breast Cancer (A) High expression of PTTG3P indicates a poor RFS of breast cancer (Kaplan-Meier Plotter database); (B) high expression of PTTG3P indicates a poor DMFS of breast cancer (Kaplan-Meier Plotter database); (C and D) high expression of PTTG3P indicates a poor OS of breast cancer (Kaplan-Meier Plotter database); (E) high expression of PTTG3P indicates a poor prognosis of breast cancer (UALCAN database); and (F) high expression of PTTG3P indicates a poor prognosis of breast cancer (OncoLnc database).

Next, the prognostic value of pseudogene PTTG3P mRNA in human breast cancer was further evaluated, using three online databases, including the Kaplan-Meier Plotter, UALCAN, and OncoLnc (Figure 3). As shown in Figures 3A–3D (Kaplan-Meier Plotter), we found that higher expression of pseudogene PTTG3P indicated poorer relapse-free survival (RFS, p = 0.00033), distant metastasis-free survival (DMFS, p = 0.025) and overall survival (OS, p = 0.0083/8.8e−05). The analytic result from UALCAN database showed that high expression of pseudogene PTTG3P suggested unfavorable prognosis of patients with breast cancer (P = 0.048). The data from the OncoLnc database also revealed that high expression of PTTG3P was significantly associated with poor prognosis (p = 0.0265). All these findings indicate that PTTG3P may serve as a poor predictor of prognosis of patients with breast cancer.

### Construction of Potential Pseudogene PTTG3P-miRNA-mRNA Regulatory Network

**Figure 4:**
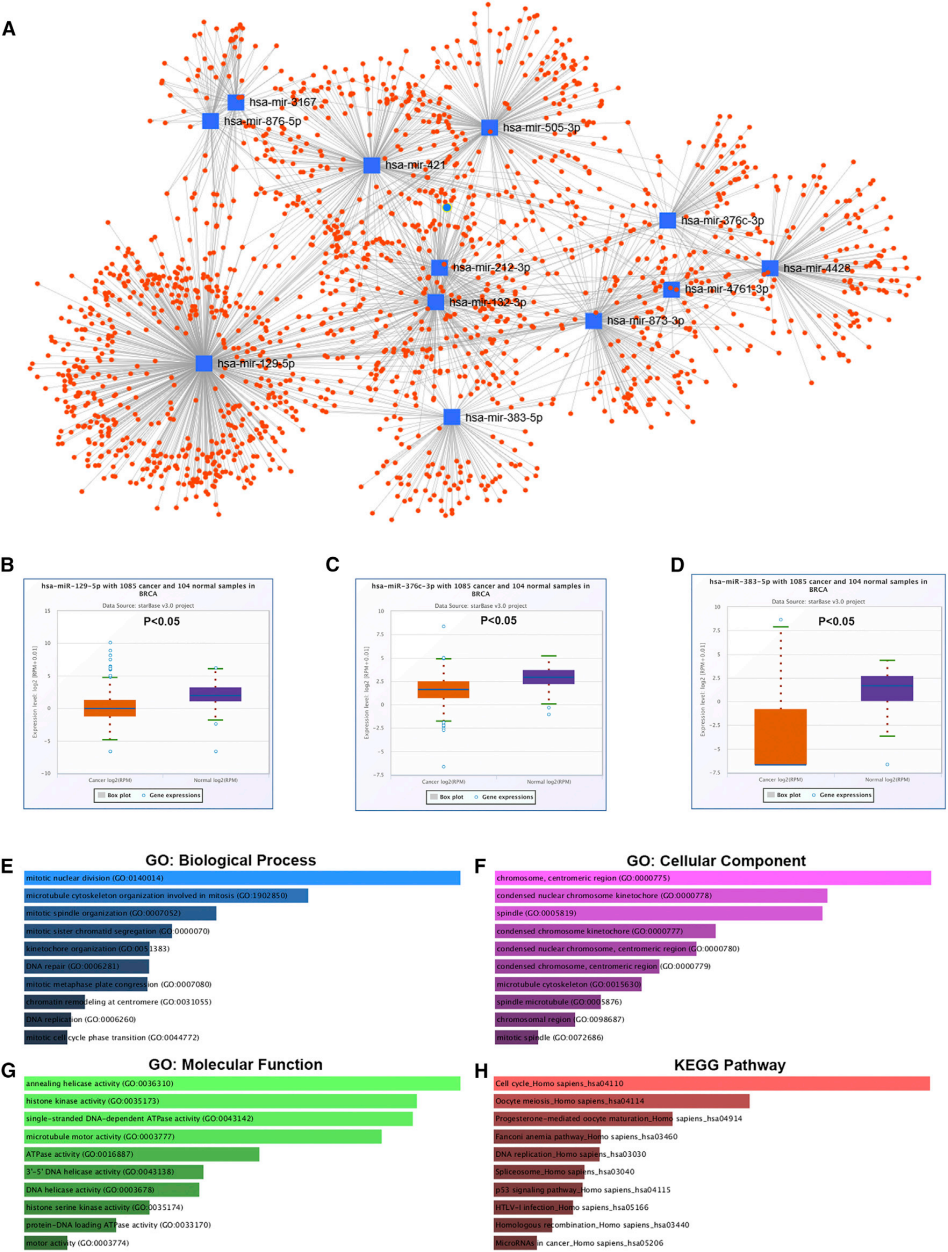
Construction of PTTG3P-miRNA-mRNA Network and Gene Ontology Functional Annotation and KEGG Pathway Enrichment Analysis for the Co-expressed Genes of PTTG3P (A) The potential pseudogene PTTG3P-miRNA-mRNA regulatory network; (B) expression of miR-129-5p in breast cancer tissues and normal controls determined by starBase v3.0; (C) expression of miR-376c-3p in breast cancer tissues and normal controls determined by starBase v3.0; (D) expression of miR-383-5p in breast cancer tissues and normal controls determined by starBase v3.0; (E) the top 10 enriched biological process (BP) items for the co-expressed genes of PTTG3P; (F) the top 10 enriched cellular component (CC) items for the co-expressed genes of PTTG3P; (G) the top 10 enriched molecular function (MF) items for the co-expressed genes of PTTG3P; and (H) the top 10 enriched KEGG pathway items for the co-expressed genes of PTTG3P.

Subsequently, we explored potential regulatory mechanisms of pseudogene PTTG3P, among which the competing endogenous RNA (ceRNA) hypothesis may be a representative approach.^7^ Twelve miRNAs (miR-132-3p, miR-129-5p, miR-212-3p, miR-876-5p, miR-3167, miR-873-3p, miR-4428, miR-4761-3p, miR-421, miR-376c-3p, miR-505-3p, and miR-383-5p) were identified as the candidate miRNAs for pseudogene PTTG3P by starBase v3.0, and 1,713 potential targets of these identified miRNAs were predicted using miRNet (Figure 4A). These miRNA-target gene pairs are listed in Table S2. The pseudogene PTTG3P-miRNA-mRNA regulatory network was finally constructed. Based on the ceRNA mechanism, expression of downstream miRNAs of PTTG3P in breast cancer samples should be decreased when compared with normal controls. Therefore, we further determined the expression of the 12 potential miRNAs in breast cancer by using The Cancer Genome Atlas (TCGA) data (Table S3). Expression levels of miR-129-5p, miR-376c-3p, and miR-383-5p were significantly lower in breast cancer tissues than that in normal tissues, as presented in Figures 4B–4D, respectively. Our current findings suggest that pseudogene PTTG3P may exert oncogenic roles via this miRNA-mRNA regulatory network. However, more experiments need to be conducted to further validate these findings.

### Co-expressed Genes of Pseudogene PTTG3P and Functional Enrichment Analysis

Identifying the co-expressed genes would facilitate a better understanding of potential functions of PTTG3P in breast cancer. Thus, the co-expressed genes of pseudogene PTTG3P were predicted by using the UALCAN database, and a total of 220 co-expressed genes were finally obtained, as presented in Table S4. Next, the STRING database was introduced, to construct a protein-protein interaction (PPI) network for these co-expressed genes (Figure S1). Moreover, we performed functional analysis for these genes. Three categories of Gene Ontology (GO) functional annotation were included, using the Enrichr database: biological process (BP), cellular component (CC), and molecular function (MF). As presented in Figures 4E–4G, the enriched GO functions for these co-expressed genes included mitotic nuclear division (GO:0140014), microtubule cytoskeleton organization involved in mitosis (GO:1902850), and mitotic spindle organization (GO:0007052) in the BP category; the chromosome, centromeric region (GO:0000775), condensed nuclear chromosome kinetochore (GO:0000778), and spindle (GO:0005819) in the CC category; and annealing helicase activity (GO:0036310), histone kinase activity (GO:0035173), and single-stranded DNA-dependent ATPase activity (GO:0043142) in the MF category. Subsequently, we conducted KEGG (Kyoto Encyclopedia of Genes and Genomes) pathway enrichment analysis (Figure 4E). The top 10 enriched KEGG pathways were cell cycle (hsa04110), oocyte meiosis (hsa04114), progesterone-mediated oocyte maturation (hsa04914), Fanconi anemia pathway (hsa03460), DNA replication (hsa03030), spliceosome (hsa03040), p53 signaling pathway (hsa04115), HTLV-I infection (hsa05166), homologous recombination (hsa03440), and microRNAs in cancer (hsa05206).

### Relationship between Pseudogene PTTG3P and Its Parental Genes PTTG1 and -2

**Figure 5:**
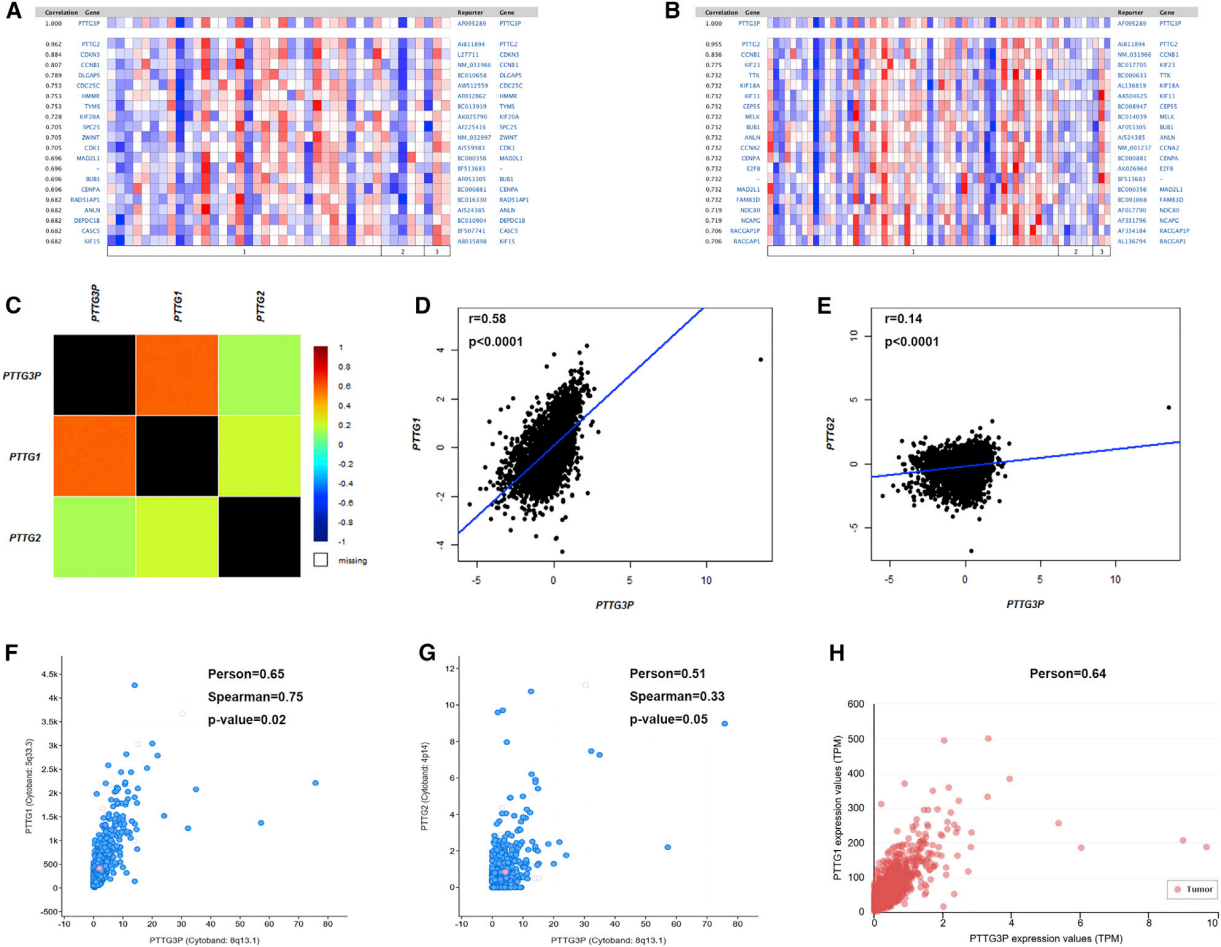
Expression Correlation of PTTG3 with Parental Genes Analyzed by Oncomine, bc-GenExMiner, cBioPortal, and UALCAN Databases (A and B) PTTG3P co-expression of genes analyzed using Oncomine database; (C–E) expression relationship between PTTG3P and PTTG1/PTTG2 in breast cancer analyzed using bc-GenExMiner database; (F and G) expression correlation between PTTG3P and PTTG1/PTTG2 in breast cancer analyzed using cBioPortal database; and (H) expression correlation between PTTG3P and -1 in breast cancer analyzed using the UALCAN database.

**Figure 6:**
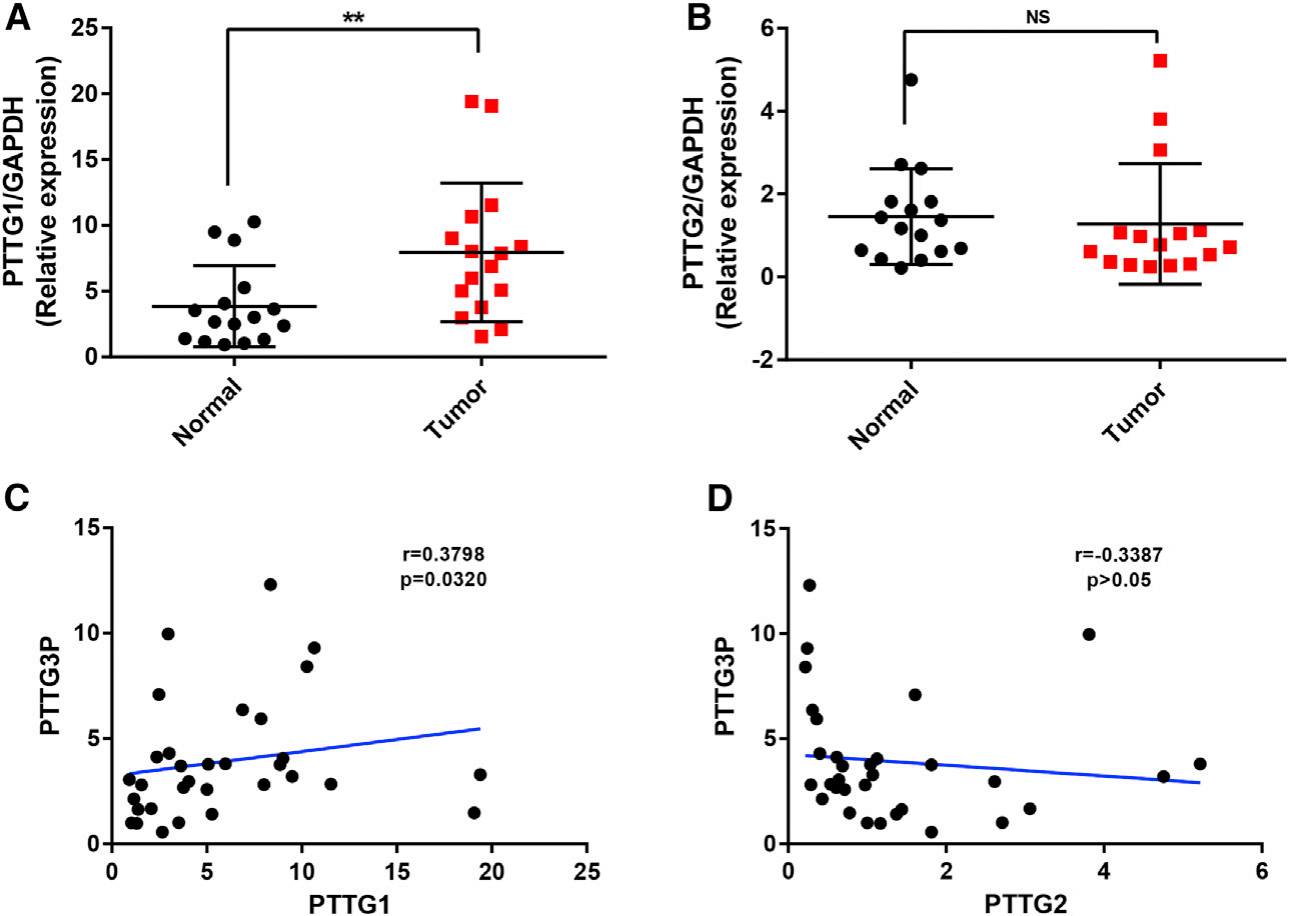
Expression of PTTG1/PTTG2 and Correlation between PTTG3P Expression and PTTG1/PTTG2 Expression in Clinical Breast Cancer Samples (A) The level of PTTG1 was detected in 16 breast cancer tissues and paired non-tumor tissues by qRT-PCR; (B) the level of PTTG2 was detected in 16 breast cancer tissues and paired non-tumor tissues by qRT-PCR; (C) the correlation of PTTG3P with PTTG1 in the 32 clinical samples; (D) the correlation of PTTG3P with PTTG2 in the 32 clinical samples. Error bars represent SD. **p < 0.01; ns represents no significance.

It has been well documented that pseudogenes can regulate their parental genes by multiple ways in the pathogenesis of human cancers.^16^, ^17^ PTTG1 and -2 are two parental genes of PTTG3P. The correlation of pseudogene PTTG3P and -1 or -2 was first analyzed using several databases, such as Oncomine, bc-GenExMiner, cBioPortal, and UALCAN (Figure 5). As shown in Figures 5A, 5B, and S2, Oncomine co-expression analysis indicated that PTTG1 and -2 ranked as the two top correlated genes among all correlated genes. Similar results were acquired by analytic data from bc-GenExMiner and cBioPortal, as presented in Figures 5C–5G. Data mining in the UALCAN database also confirmed a positive correlation of PTTG1 and -3P expression (Figure 5H). However, no significant expression association between PTTG3P and -2 was found. To confirm accuracy of these analytic results, experimental validation was further performed via detection of the expression levels of PTTG1 and -2 and assessment of the expression correlation of PTTG3P with PTTG1 and -2 in clinical breast cancer patients. As shown in Figure 6A, the expression level of PTTG1 in breast cancer samples was markedly higher than that in matched normal breast samples. A positive expression correlation of PTTG3P with PTTG1 was observed (Figure 6C). However, there was no significant difference in PTTG2 expression between tumor and normal tissues (Figure 6B). Figure 6D demonstrates that PTTG3P expression did not correlate significantly with PTTG2 expression. All these findings suggest a positive correlation of PTTG3P expression with PTTG1 expression, implying that PTTG3P may promote breast cancer progression by upregulating PTTG1. However, it needs to be further validated by much more study.

## Discussion

Pseudogenes have long been dismissed as junk genes. However, in recent years, growing evidence has demonstrated key roles of pseudogenes in physiological and pathological processes. The dysregulation of pseudogenes has a close connection with human disorders, especially cancer.^18^, ^19^, ^20^, ^21^ Several cancer-associated pseudogenes have been identified, including PTENP1,^22^ HMGA1P6, HMGA1P7,^23^ POU5F1B,^24^ FOXO3P,^25^ and INTS6P1.^26^ PTTG3P is a novel pseudogene, which has been found to be closely associated with the onset and progression of some types of human cancer, such as gastric cancer and hepatocellular carcinoma.^14^, ^15^ However, the expression pattern and potential roles of PTTG3P in breast cancer, as well as its underlying regulatory mechanisms, remain largely unknown.

In this study, we first determined the expression level of PTTG3P in breast cancer, based on bioinformatics analyses and experimental validation. The SAGE Digital Gene Expression Display showed no macroscopic differences in the expression of PTTG3P in the cancer and normal groups, partially because of the gene’s extremely low overall expression. Oncomine analysis revealed that expression of PTTG3P in breast cancer tissues was significantly higher than that in normal tissues. qRT-PCR for clinical samples also demonstrated a high expression level of PTTG3P in breast cancer tissues when compared with their matched normal tissues. Moreover, a negative association between PTTG3P expression and ER and PR status was found. Conversely, basal-like status, TNBC status, NPI, or SBR grade was positively linked to PTTG3P expression. Subsequently, the Kaplan-Meier Plotter, UALCAN, and OncoLnc databases were used to study the prognostic role of PTTG3P in breast cancer, and the analytic results displayed that high expression of PTTG3P indicated poor RFS, OS, and DMFS. These findings suggest that increased expression of PTTG3P may be a promising prognostic biomarker in breast cancer.

Pseudogenes exert their functions in three levels, including DNA, RNA, and protein by a variety of mechanisms, involving gene conversion, homologous recombination, exonization, insertional mutations, and serving as antisense RNAs, endo-siRNAs, or competing endogenous RNAs.^4^ To explore the potential regulatory mechanisms of PTTG3P, we first constructed a pseudogene-miRNA-mRNA interaction network. In this network, 12 potential miRNAs were identified as candidates for PTTG3P and 1,713 target genes for the identified miRNAs were finally predicted. Next, we further determined the expression levels of the 12 potential miRNAs in breast cancer and found that 3 of 12 (miR-129-5p, miR-376c-3p, and miR-383-5p) miRNAs were significantly downregulated in breast cancer. According to the ceRNA hypothesis, the three miRNAs may be three downstream miRNAs. Moreover, previous studies have confirmed that all three miRNAs function as tumor suppressors in various types of human cancers, including breast cancer.^27^, ^28^, ^29^, ^30^, ^31^ Co-expression analysis may also provide critical clues for investigating the mechanisms of PTTG3P. UALCAN database analysis acquired 220 positively correlated genes. Functional annotation and KEGG pathway enrichment analysis showed that the co-expressed genes were mainly enriched in nuclear division, cell cycle, and oocyte meiosis. Therefore, we supposed that PTTG3P exerted its roles in cancer by these biological processes.

Numerous studies have suggested that pseudogenes can regulate their parental genes and thereby are involved in different cellular processes.^16^ PTTG1 and -2 are two parental genes of PTTG3P. It has been well documented that PTTG1 and -2 act as two oncogenes in multiple human cancers.^12^, ^32^, ^33^ We found that PTTG3P expression was significantly positively correlated with PTTG1 expression in the Oncomine, bc-GenExMiner, cBioPortal, and UALCAN databases, but was markedly associated with PTTG2 expression only in the Oncomine and bc-GenExMiner databases. The correlation of PTTG3P with PTTG1 or -2 was further assessed in clinical samples. The results revealed that PTTG3P correlated positively only with PTTG1. Previous studies have suggested that PTTG1 exerts oncogenic roles mainly by regulating the cell cycle.^15^, ^33^ These findings and the present results provide ample evidence that PTTG3P may promote breast cancer progression through modulation of cell cycle-associated processes by partially upregulating expression of its parental genes, especially PTTG1.

**Figure 7:**
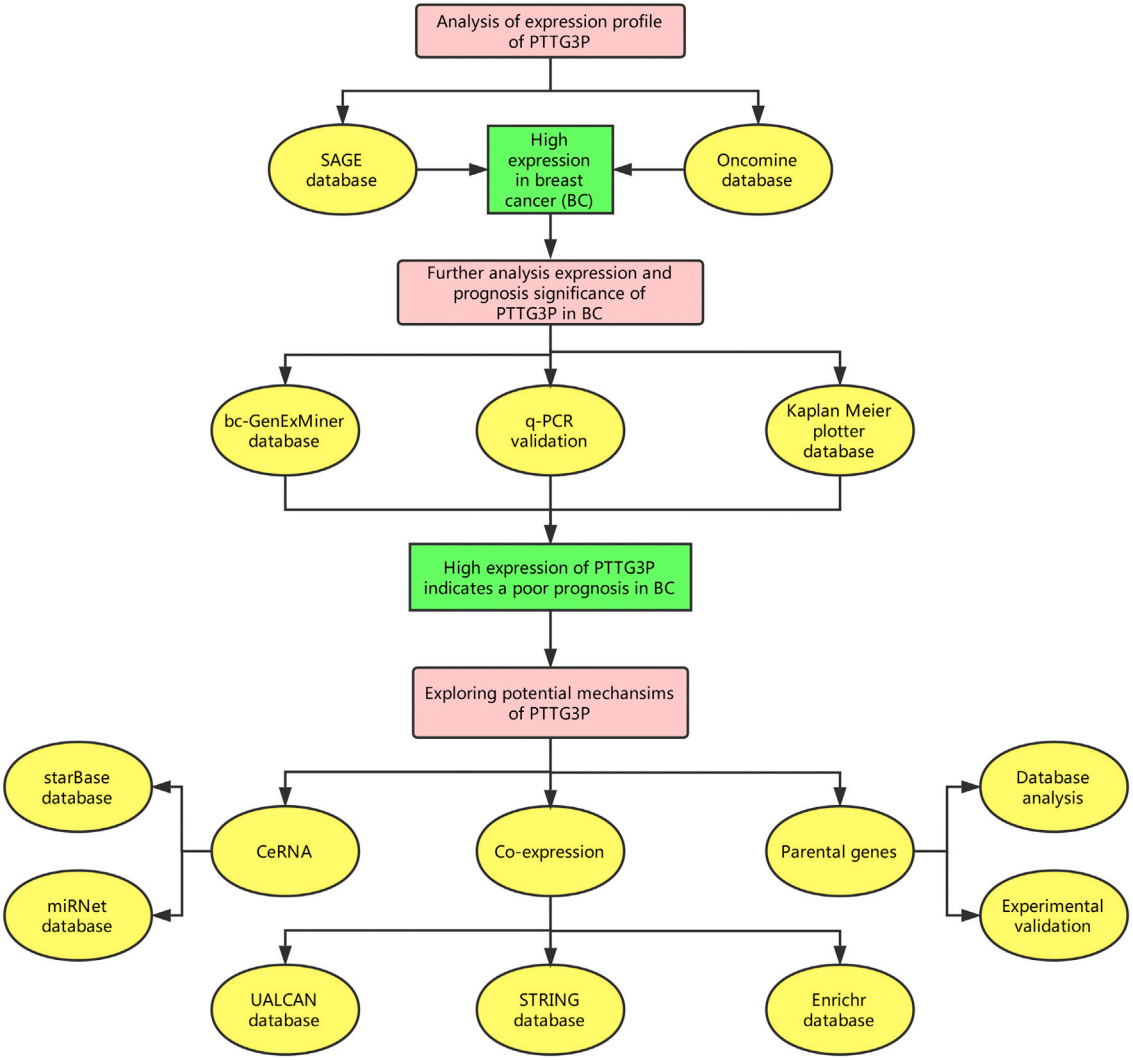
The Visual Flow-Process Diagram of This Study

Altogether, based on bioinformatics analysis and experimental validation (Figure 7), we provided a reliable integrated analysis of the pseudogene PTTG3P expression pattern, prognostic value, and potential regulatory mechanisms in breast cancer. In this study, high expression of pseudogene PTTG3P in breast cancer was confirmed, and increased expression of PTTG3P indicated a poor prognosis of breast cancer. Furthermore, a potential pseudogene PTTG3P-miRNA-mRNA regulatory network was constructed, and the three most possible downstream miRNAs (miR-129-5p, miR-376c-3p, and miR-383-5p) of PTTG3P were identified. Our findings also supported that PTTG3P may upregulate PTTG1 expression, thus functioning as an oncogene in breast cancer. In summary, pseudogene PTTG3P may serve as a novel prognostic biomarker and a therapeutic target for patients with breast cancer. In the future, more laboratory experiments and clinical trials need to be performed to further validate the findings in this study.

## Materials and Methods

### Expression Profile Analysis Using Serial Analysis of Gene Expression and Oncomine

The expression profile of pseudogene PTTG3P was analyzed with two online databases, including SAGE and Oncomine. First, all available published SAGE data were used to analyze PTTG3P expression in 23 normal human tissues and corresponding malignant counterparts, and PTTG3P expression profile was displayed using the SAGE Anatomic Viewer (http://cgap.nci.nih.gov/SAGE/AnatomicViewer/). We also determined the PTTG3P expression profile using Oncomine, which is a cancer microarray database and an integrated data-mining platform, to facilitate discovery from genome-wide expression analyses.^34^ To date, the Oncomine database has 19 cancer types containing 92 datasets. Oncomine analysis of cancer versus normal samples in these 19 cancer types was conducted. p < 0.05, |fold change| > 1.5 and gene rank in the top 10% were set as the thresholds. We also conducted a meta-analysis of datasets in breast cancer. The Oncomine database was also used to perform a co-expression analysis of PTTG3P and other genes.

### Breast Cancer Gene-Expression Miner

Breast cancer gene-expression miner (bc-GenExMiner), a statistical mining tool of published annotated genomic data, was introduced to assess the correlation of pseudogene PTTG3P expression level with specific clinicopathological features of breast cancer, including age, nodal status, hormonal receptors status (ER and PR), HER2, pathological subtype, NPI, and SBR grade.^35^, ^36^ bc-GenExMiner was also used to determine the expression correlation of PTTG3P with PTTG1 and 2. p < 0.05 was considered statistically significant.

### Survival Analysis Using Kaplan-Meier Plotter, UALCAN, and OncoLnc Databases

The Kaplan-Meier Plotter (http://kmplot.com/analysis/), an online database established from gene expression data and survival information of cancer patients downloaded from the Gene Expression Omnibus (GEO) database, was used to evaluate the prognostic value of PTTG3P in breast cancer.^37^ PTTG3P was first entered into the database. A Kaplan-Meier survival plot, hazards ratio (HR), 95% confidence interval (CI), and log rank p were directly determined and displayed on the webpage. A log rank p < 0.05 was considered statistically significant.

UALCAN (http://ualcan.path.uab.edu/index.html/) is a portal for facilitating tumor subgroup gene expression and survival analyses, providing easy access to publicly available cancer transcriptome data containing TCGA.^38^ It was employed to evaluate the effect of PTTG3P on breast cancer patient survival. p < 0.05 was considered statistically significant.

OncoLnc (http://www.oncolnc.org/), a database for interactively investigating survival correlations and retrieving clinical data associated with expression data for mRNAs, miRNAs, long non-coding RNAs, and pseudogenes, was also used to assess the prognostic value of PTTG3P in breast cancer.^6^ p < 0.05 was considered statistically significant.

### Construction of the Pseudogene-miRNA-mRNA Regulatory Network

The pseudogene PTTG3P-miRNA-mRNA regulatory network was constructed. First, starBase v3.0 (http://starbase.sysu.edu.cn/) was employed to identify potential miRNAs binding to pseudogene PTTG3P.^39^ Subsequently, target genes for identified miRNAs were predicted using the miRNet database (https://www.mirnet.ca/), which is an easy-to-use tool for comprehensive statistical analysis and functional interpretation of data from miRNAs studies.^40^ Expression levels of the potential miRNAs in breast cancer were also detected by starBase v3.0 (http://starbase.sysu.edu.cn/). p < 0.05 was considered statistically significant.

### Co-expression Analysis

Those genes co-expressed with pseudogene PTTG3P were analyzed using UALCAN.^41^ Genes positively correlated with PTTG3P in breast cancer were downloaded from UALCAN. Genes with extremely low expression (median transcripts per million < 0.5) were filtered out, and only genes with a Pearson correlation coefficient ≥ 0.3 were included.

### Protein-Protein Interaction Network

Protein-protein interaction network for the co-expressed genes was established using STRING v10.5 (https://string-db.org/cgi/input.pl/), which is a database of predicted functional associations between proteins.^42^ The network displayed on the webpage was gathered into three clusters and then exported as a high-resolution bitmap.

### Gene Ontology and KEGG Pathway Enrichment Analysis

The Enrichr database (http://amp.pharm.mssm.edu/Enrichr/) was employed to perform GO functional annotation and KEGG pathway enrichment analysis for these co-expressed genes.^43^ GO functional analysis contained three categories, including BP, CC, and MF. The bar charts of GO and KEGG analysis for these co-expressed genes were directly downloaded from the webpage.

### cBioPortal Database Analysis

In addition to using the Oncomine, bc-GenExMiner, and UALCAN databases mentioned above, we conducted a correlation analysis of pseudogene PTTG3P and its parental gene PTTG1 and -2 using the cBioPortal database (http://www.cbioportal.org/).^44^ p < 0.05 was considered statistically significant.

### Gathering of Clinical Samples and Ethics Statement

Altogether, 50 clinical breast cancer tissues and matched normal tissues were collected from the Zhejiang Cancer Hospital (Hangzhou, China). All the samples were kept in liquid nitrogen until the extraction of RNA. Written informed consent for the biological experiments was obtained from every patient involved in the study, and the study was approved by the Ethics Committee of the Zhejiang Cancer Hospital.

### RNA Extraction and qRT-PCR Analysis

RNA extraction and qRT-PCR were performed as described previously.^38^, ^45^, ^46^ The primers used in this study were purchased from Ribobio (Guangzhou, China) and are listed in Table S5.

### Statistical Analysis

Each experiment was performed in triplicate and repeated at least three times. GraphPad Prism Software, Version 7, was introduced to conduct statistical analysis. The statistical significance of the differences between two groups was estimated by two-tailed Student’s t test. Spearman correlation coefficients were calculated to evaluate the association between PTTG3P and -1 or -2.

## Author Contributions

Conceptualization, W.L. and W.F.; methodology, W.L. and B.D.; software, W.L. and B.D.; writing of the original draft, W.L.; writing review and editing, W.L., B.D. and W.F.; and funding acquisition, W.F.

## Conflicts of Interest

The authors declare no competing interests.

## Supplemental Information

Document S1. Figures S1 and S2 and Tables S1–S5

Document S2. Article plus Supplemental Information
