# Supplementary material for: High Expression of Pseudogene PTTG3P Indicates a Poor Prognosis in Human Breast Cancer
Source: Mol Ther Oncolytics. 2019 Mar 27;14:15–26. doi: 10.1016/j.omto.2019.03.006 (PMC6463746; doi:10.1016/j.omto.2019.03.006)
Supplement: Document S2. Article plus Supplemental Information [file mmc2.pdf]

Molecular Therapy
Oncolytics
Original Article

AMERICAN SOCIETY of GENE & CELL THERAPY

High Expression of Pseudogene PTTG3P Indicates a Poor Prognosis in Human Breast Cancer

Weiyang Lou,[1,2,3] Bisha Ding,[1,2,3] and Weimin Fan[1,2,3,4]

[1]Program of Innovative Cancer Therapeutics, Division of Hepatobiliary and Pancreatic Surgery, Department of Surgery, First Affiliated Hospital, College of Medicine, Zhejiang University, Hangzhou 310003, China; [2]Key Laboratory of Organ Transplantation, Zhejiang Province, Hangzhou 310003, China; [3]Key Laboratory of Combined Multi-organ Transplantation, Ministry of Public Health, Hangzhou 310000, China; [4]Department of Pathology and Laboratory Medicine, Medical University of South Carolina, Charleston, SC 29425, USA

Pseudogenes play pivotal roles in tumorigenesis. Previous studies have suggested that pituitary tumor-transforming 3, pseudogene (PTTG3P), serves as an oncogene in human cancers. However, its expression pattern, biological function, and underlying mechanism in breast cancer remain unknown. In this study, we demonstrated an elevated expression of PTTG3P in breast cancer and discovered that PTTG3P expression correlated negatively with estrogen receptor (ER) and progesterone receptor (PR) status, but linked positively to basal-like status, triple-negative breast cancer status, Nottingham prognostic index (NPI), and Scarff-Bloom-Richardson grade. High expression of PTTG3P was also found to be associated with a poor prognosis of breast cancer. To explore the potential mechanisms of PTTG3P, a PTTG3P-microRNA (miRNA)-mRNA regulatory network was established. Co-expressed genes of PTTG3P were also obtained. Enrichment analysis for these co-expressed genes revealed that they were significantly enriched in mitotic nuclear division and cell cycle. Subsequent research on mechanism of PTTG3P indicated that its expression correlated positively with PTTG1 expression. However, no significant expression correlation between PTTG3P and PTTG2 was observed. Taken together, our findings suggest that increased expression of pseudogene PTTG3P may be used as a promising prognostic biomarker and novel therapeutic target for breast cancer.

INTRODUCTION

Breast cancer is one of the most common malignancies and remains a leading cause of cancer-related deaths in the female worldwide.[1] The incidence of breast cancer is gradually increasing in most countries, including the United States.[2] Despite the huge improvements in screening, diagnosis, and treatment of breast cancer that have been achieved, the patient's prognosis is still dismal. Thus, it is extremely meaningful and also urgent to search and develop more sensitive and specific prognostic biomarkers for patients with breast cancer.

Pseudogenes, a class of non-coding RNAs and segments of DNA that are related to real functional genes, are traditionally recognized as "junk genes" or "genomic fossils" because they have lost functionality.[3] Based on the formation mechanism, pseudogenes are generally classified into three subtypes: unitary pseudogenes, duplicated pseudogenes, and processed pseudogenes.[4] Recent studies have reported that pseudogenes play important roles in regulating parental genes by functioning as antisense RNAs, small-interference RNAs (siRNAs), or endogenous competitors for microRNA (miRNA), RNA-binding protein, or translational machinery.[4] Pseudogenes are frequently dysregulated in multiple human cancers, and the dysregulation results in cancer onset and progression.[5–7]

Pituitary tumor-transforming 3, pseudogene (PTTG3P), is a processed pseudogene that shows high homology to its family members, pituitary tumor-transforming 1 (PTTG1) and -2 (PTTG2).[8] Numerous investigations have shown that PTTG1 and -2 serve as two key oncogenes in a variety of human cancers, including breast cancer,[9,10] lung cancer,[11] pituitary adenoma,[12] and esophageal squamous cell cancer.[13] However, there are only a few reports regarding the expression and roles of PTTG3P in cancer. One study, conducted by Weiwei Weng et al.,[14] revealed that PTTG3P promotes proliferation and invasion and is an indicator of poor prognosis in gastric cancer. Huang et al.[15] found that PTTG3P enhances hepatocellular carcinoma (HCC) growth and metastasis via upregulating PTTG1 and activating PI3K/AKT signaling. Notably, the expression, function, and corresponding regulatory mechanisms of PTTG3P in breast cancer remain unclear and need to be further elucidated.

Herein, we first assessed the expression pattern of PTTG3P in human breast cancer by data mining and experimental validation. Then, the prognostic significance of PTTG3P expression in breast cancer was also analyzed. Finally, we explored several potential mechanisms of PTTG3P in breast cancer, including construction of the pseudogene

Received 1 December 2018; accepted 13 March 2019;
https://doi.org/10.1016/j.omto.2019.03.006.

Correspondence: Weiyang Lou, Program of Innovative Cancer Therapeutics, Division of Hepatobiliary and Pancreatic Surgery, Department of Surgery, First Affiliated Hospital, College of Medicine, Zhejiang University, Hangzhou 310003, China.
E-mail: 11718264@zju.edu.cn
Correspondence: Weimin Fan, Program of Innovative Cancer Therapeutics, Division of Hepatobiliary and Pancreatic Surgery, Department of Surgery, First Affiliated Hospital, College of Medicine, Zhejiang University, Hangzhou 310003, China.
E-mail: fanw@zju.edu.cn

This is an open access article under the CC BY license (http://creativecommons.org/licenses/by/4.0/).

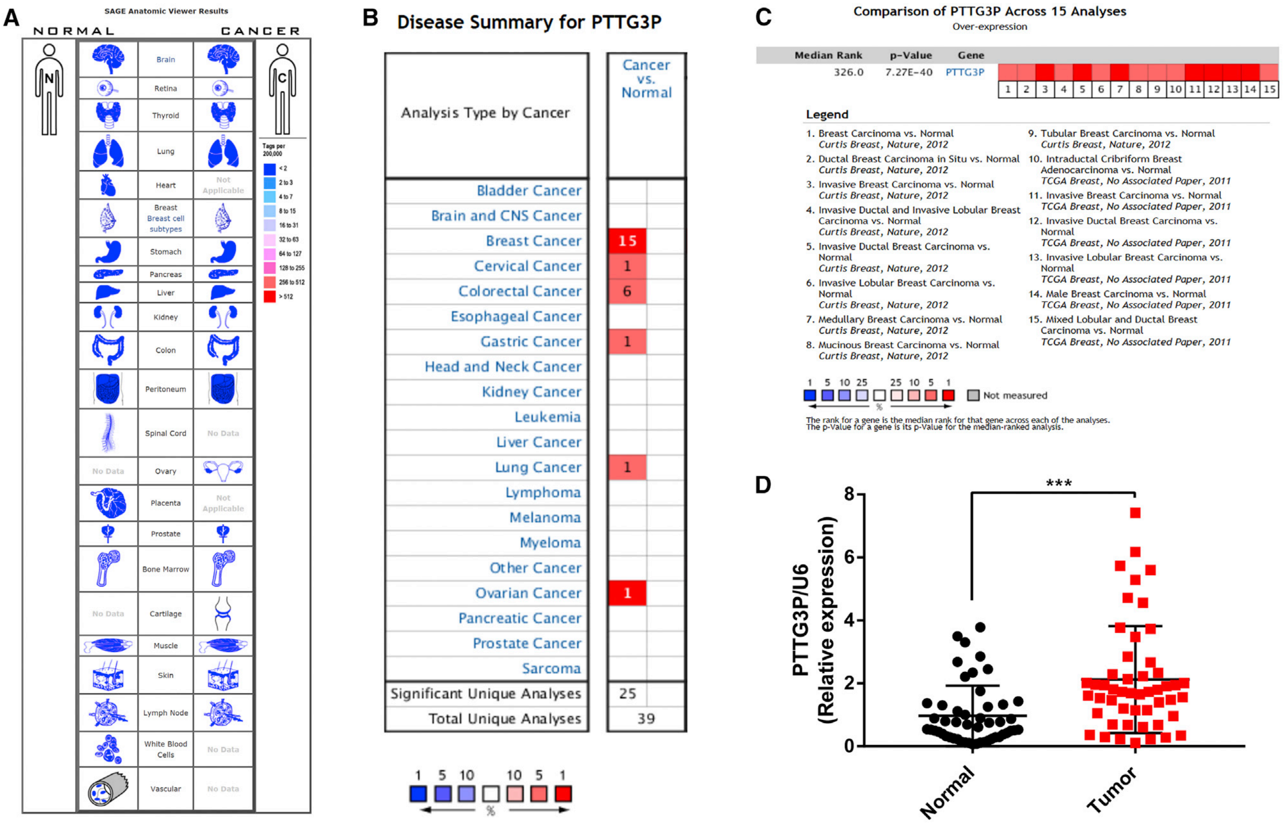

Figure 1. Expression Pattern of PTTG3P in Human Breast Cancer

(A) Expression profile of PTTG3P determined by the SAGE Digital Gene Expression Displayer; (B) expression of PTTG3P in different types of cancers by Oncomine analysis of cancer versus normal samples; (C) expression of PTTG3P in breast cancer by Oncomine meta-analysis; and (D) expression of PTTG3P in breast cancer samples compared with matched normal samples. Error bars represent SD. ***$p < 0.001$.

PTTG3P-miRNA-mRNA network, analysis of co-expressed genes, and assessment of its correlation with parental genes PTTG1 and -2. These findings facilitate understanding of PTTG3P's roles and mechanisms in human breast cancer.

RESULTS

Pseudogene PTTG3P Expression in Human Breast Cancer

The expression profile of pseudogene PTTG3P in human breast cancer was first determined by using the SAGE (Serial Analysis of Gene Expression) Digital Gene Expression Display. As shown in Figure 1A, pseudogene PTTG3P expression was not significantly dysregulated in all cancer tissues when compared with corresponding normal tissues, including breast cancer. Next, Oncomine analysis of cancer versus normal samples was used to analyze the expression profile of pseudogene PTTG3P in human breast cancer (Figure 1B). Oncomine meta-analysis was further employed to assess the comprehensive median expression of pseudogene PTTG3P across 15 analyses (Figure 1C). As presented in Table 1, expression of pseudogene PTTG3P was significantly higher in medullary breast carcinoma, invasive breast carcinoma, invasive lobular breast carcinoma, invasive ductal breast carcinoma, mucinous breast carcinoma, ductal breast carcinoma *in situ*, male breast carcinoma, intraductal cribriform breast adenocarcinoma, and mixed lobular and ductal breast carcinoma. For further validation, we also detected the expression of pseudogene PTTG3P in clinical breast cancer samples. As shown in Figure 1D, pseudogene PTTG3P expression in breast cancer tissues was markedly higher than that in normal tissues. All evidence taken together, by combining bioinformatics analysis and experimental validation, we demonstrated that PTTG3P was significantly overexpressed in human breast cancer.

We further investigated the associations between pseudogene PTTG3P expression and different clinicopathological characteristics in patients with breast cancer, by using bc-GenExMiner (Figure 2; Table S1). Analytic results suggested that there are no significant differences between the ≤51- and >51-year group ($p = 0.4159$). Nodal and HER2 status also showed no significant differences between the positive and negative groups (nodal status: $p = 0.1266$; HER2 status: $p = 0.1423$). Estrogen receptor (ER) and progesterone receptor (PR) status were discovered to be negatively linked to pseudogene PTTG3P expression (ER status: $p < 0.0001$; PR status: $p < 0.0001$). Pseudogene PTTG3P was significantly upregulated in basal-like or triple-negative breast cancer when compared with their counterparts (basal-like

Table 1. Significant Changes of PTTG3P Expression in the Transcriptional Level between Different Types of Breast Cancer and Normal Tissues (Oncomine Database)

Subtype of Breast Cancer	p Value	Fold Change	t Test	Rank	Sample Count	Reporter ID
Medullary breast carcinoma	1.52E−19	6.164	17.706	1%	176	ILMN_2049021
Invasive breast carcinoma	3.63E−9	3.270	9.087	1%	165	ILMN_2049021
Invasive ductal breast carcinoma	6.87E−111	3.429	39.869	1%	1700	ILMN_2049021
Invasive lobular breast carcinoma	7.27E−40	2.371	16.045	2%	292	ILMN_2049021
Invasive ductal and invasive lobular breast carcinoma	3.69E−29	2.791	14.788	2%	234	ILMN_2049021
Mucinous breast carcinoma	1.77E−15	2.462	10.742	3%	190	ILMN_2049021
Ductal breast carcinoma *in Situ*	2.13E−4	2.508	5.291	4%	154	ILMN_2049021
Breast carcinoma	3.76E−5	2.915	5.595	5%	158	ILMN_2049021
Tubular breast carcinoma	7.17E−20	2.325	11.684	5%	211	ILMN_2049021
Male breast carcinoma	1.14E−17	2.467	12.903	1%	64	A_23_P60016
Invasive ductal breast carcinoma	1.35E−45	4.791	24.131	1%	450	A_23_P60016
Invasive lobular breast carcinoma	1.05E−16	3.327	11.193	1%	97	A_23_P60016
Invasive breast carcinoma	2.86E−27	3.547	13.653	1%	137	A_23_P60016
Intraductal cribriform breast adenocarcinoma	6.92E−6	3.115	9.941	3%	64	A_23_P60016
Mixed lobular and ductal breast carcinoma	7.72E−5	2.553	6.204	4%	68	A_23_P60016

status: $p < 0.0001$; triple-negative status: $p < 0.0001$). The Nottingham prognostic index (NPI) is often used to determine prognosis after surgery for breast cancer. A higher NPI correlated significantly with higher expression of pseudogene PTTG3P expression. Similarly, a more advanced Scarff-Bloom-Richardson (SBR) grade was associated with higher expression of pseudogene PTTG3P expression. To summarize, these results demonstrate that pseudogene PTTG3P expression may play an unfavorable role in patients with breast cancer.

Prognostic Significance of the Pseudogene PTTG3P in Human Breast Cancer

Next, the prognostic value of pseudogene PTTG3P mRNA in human breast cancer was further evaluated, using three online databases, including the Kaplan-Meier Plotter, UALCAN, and OncoLnc (Figure 3). As shown in Figures 3A–3D (Kaplan-Meier Plotter), we found that higher expression of pseudogene PTTG3P indicated poorer relapse-free survival (RFS, p = 0.00033), distant metastasis-free survival (DMFS, p = 0.025) and overall survival (OS, p = 0.0083/8.8e−05). The analytic result from UALCAN database showed that high expression of pseudogene PTTG3P suggested unfavorable prognosis of patients with breast cancer (P = 0.048). The data from the OncoLnc database also revealed that high expression of PTTG3P was significantly associated with poor prognosis (p = 0.0265). All these findings indicate that PTTG3P may serve as a poor predictor of prognosis of patients with breast cancer.

Construction of Potential Pseudogene PTTG3P-miRNA-mRNA Regulatory Network

Subsequently, we explored potential regulatory mechanisms of pseudogene PTTG3P, among which the competing endogenous RNA (ceRNA) hypothesis may be a representative approach.[7] Twelve miRNAs (miR-132-3p, miR-129-5p, miR-212-3p, miR-876-5p, miR-3167, miR-873-3p, miR-4428, miR-4761-3p, miR-421, miR-376c-3p, miR-505-3p, and miR-383-5p) were identified as the candidate miRNAs for pseudogene PTTG3P by starBase v3.0, and 1,713 potential targets of these identified miRNAs were predicted using miRNet (Figure 4A). These miRNA-target gene pairs are listed in Table S2. The pseudogene PTTG3P-miRNA-mRNA regulatory network was finally constructed. Based on the ceRNA mechanism, expression of downstream miRNAs of PTTG3P in breast cancer samples should be decreased when compared with normal controls. Therefore, we further determined the expression of the 12 potential miRNAs in breast cancer by using The Cancer Genome Atlas (TCGA) data (Table S3). Expression levels of miR-129-5p, miR-376c-3p, and miR-383-5p were significantly lower in breast cancer tissues than that in normal tissues, as presented in Figures 4B–4D, respectively. Our current findings suggest that pseudogene PTTG3P may exert oncogenic roles via this miRNA-mRNA regulatory network. However, more experiments need to be conducted to further validate these findings.

Co-expressed Genes of Pseudogene PTTG3P and Functional Enrichment Analysis

Identifying the co-expressed genes would facilitate a better understanding of potential functions of PTTG3P in breast cancer. Thus, the co-expressed genes of pseudogene PTTG3P were predicted by using the UALCAN database, and a total of 220 co-expressed genes were finally obtained, as presented in Table S4. Next, the STRING database was introduced, to construct a protein-protein interaction (PPI) network for these co-expressed genes (Figure S1). Moreover, we performed functional analysis for these genes. Three categories of Gene Ontology (GO) functional annotation were included, using the Enrichr database: biological process (BP), cellular component (CC),

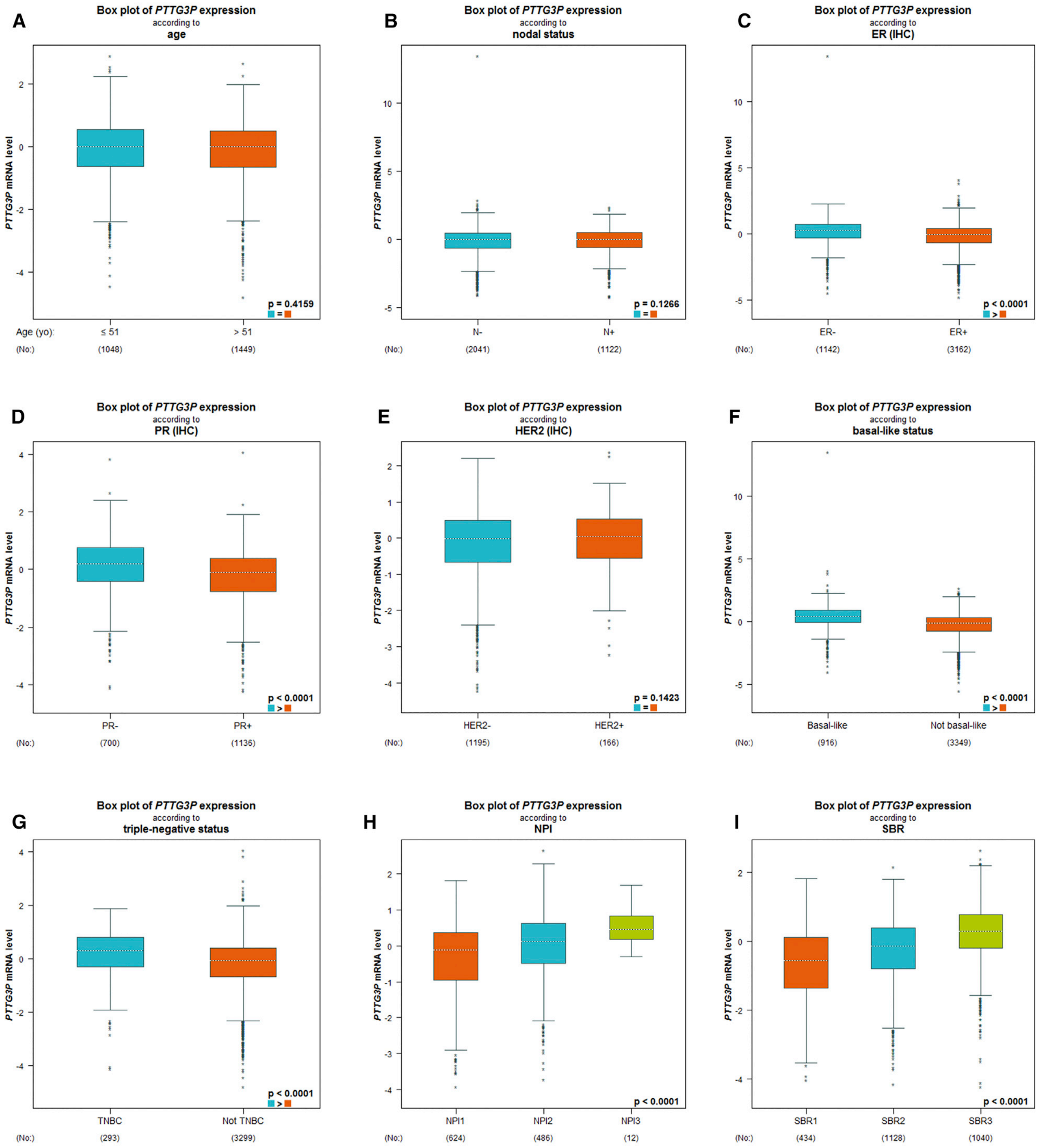

Figure 2. Relationship between Expression of PTTG3P and Various Clinicopathological Parameters of Breast Cancer Determined by the bc-GenExMiner Database

(A) Boxplot of PTTG3P expression according to age; (B) boxplot of PTTG3P expression according to nodal status; (C) boxplot of PTTG3P expression according to ER; (D) boxplot of PTTG3P expression according to PR; (E) boxplot of PTTG3P expression according to HER2; (F) boxplot of PTTG3P expression according to basal-like status; (G) boxplot of PTTG3P expression according to triple-negative status; (H) boxplot of PTTG3P expression according to NPI; and (I) boxplot of PTTG3P expression according to SBR.

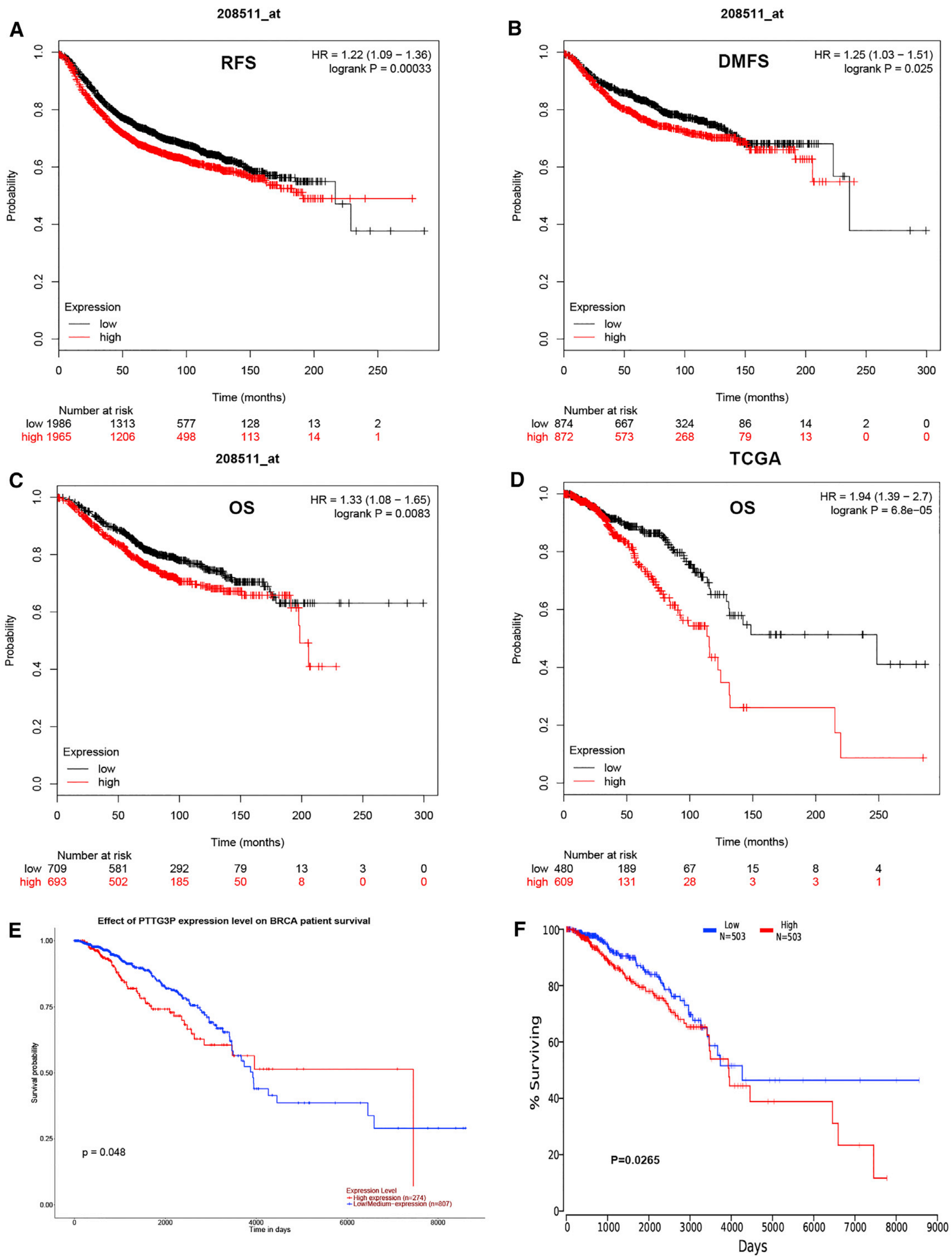

Figure 3. Prognostic Value of PTTG3P in Breast Cancer

(A) High expression of PTTG3P indicates a poor RFS of breast cancer (Kaplan-Meier Plotter database); (B) high expression of PTTG3P indicates a poor DMFS of breast cancer (Kaplan-Meier Plotter database); (C and D) high expression of PTTG3P indicates a poor OS of breast cancer (Kaplan-Meier Plotter database); (E) high expression of PTTG3P indicates a poor prognosis of breast cancer (UALCAN database); and (F) high expression of PTTG3P indicates a poor prognosis of breast cancer (OncoLnc database).

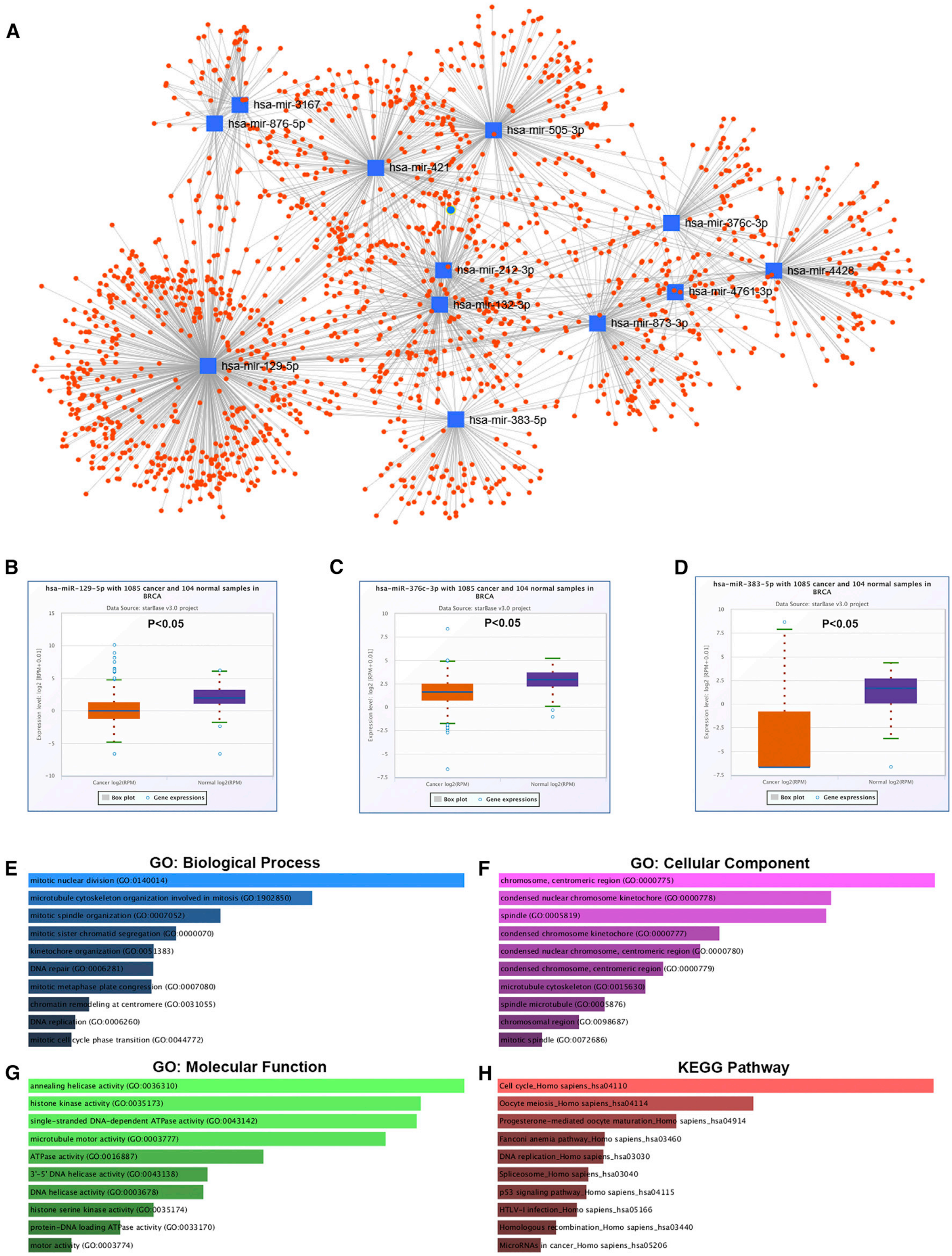

(legend on next page)

and molecular function (MF). As presented in Figures 4E–4G, the enriched GO functions for these co-expressed genes included mitotic nuclear division (GO:0140014), microtubule cytoskeleton organization involved in mitosis (GO:1902850), and mitotic spindle organization (GO:0007052) in the BP category; the chromosome, centromeric region (GO:0000775), condensed nuclear chromosome kinetochore (GO:0000778), and spindle (GO:0005819) in the CC category; and annealing helicase activity (GO:0036310), histone kinase activity (GO:0035173), and single-stranded DNA-dependent ATPase activity (GO:0043142) in the MF category. Subsequently, we conducted KEGG (Kyoto Encyclopedia of Genes and Genomes) pathway enrichment analysis (Figure 4E). The top 10 enriched KEGG pathways were cell cycle (hsa04110), oocyte meiosis (hsa04114), progesterone-mediated oocyte maturation (hsa04914), Fanconi anemia pathway (hsa03460), DNA replication (hsa03030), spliceosome (hsa03040), p53 signaling pathway (hsa04115), HTLV-I infection (hsa05166), homologous recombination (hsa03440), and microRNAs in cancer (hsa05206).

Relationship between Pseudogene PTTG3P and Its Parental Genes PTTG1 and -2

It has been well documented that pseudogenes can regulate their parental genes by multiple ways in the pathogenesis of human cancers.[16,17] PTTG1 and -2 are two parental genes of PTTG3P. The correlation of pseudogene PTTG3P and -1 or -2 was first analyzed using several databases, such as Oncomine, bc-GenExMiner, cBioPortal, and UALCAN (Figure 5). As shown in Figures 5A, 5B, and S2, Oncomine co-expression analysis indicated that PTTG1 and -2 ranked as the two top correlated genes among all correlated genes. Similar results were acquired by analytic data from bc-GenExMiner and cBioPortal, as presented in Figures 5C–5G. Data mining in the UALCAN database also confirmed a positive correlation of PTTG1 and -3P expression (Figure 5H). However, no significant expression association between PTTG3P and -2 was found. To confirm accuracy of these analytic results, experimental validation was further performed via detection of the expression levels of PTTG1 and -2 and assessment of the expression correlation of PTTG3P with PTTG1 and -2 in clinical breast cancer patients. As shown in Figure 6A, the expression level of PTTG1 in breast cancer samples was markedly higher than that in matched normal breast samples. A positive expression correlation of PTTG3P with PTTG1 was observed (Figure 6C). However, there was no significant difference in PTTG2 expression between tumor and normal tissues (Figure 6B). Figure 6D demonstrates that PTTG3P expression did not correlate significantly with PTTG2 expression. All these findings suggest a positive correlation of PTTG3P expression with PTTG1 expression, implying that PTTG3P may promote breast cancer progression by upregulating PTTG1. However, it needs to be further validated by much more study.

DISCUSSION

Pseudogenes have long been dismissed as junk genes. However, in recent years, growing evidence has demonstrated key roles of pseudogenes in physiological and pathological processes. The dysregulation of pseudogenes has a close connection with human disorders, especially cancer.[18–21] Several cancer-associated pseudogenes have been identified, including PTENP1,[22] HMGA1P6, HMGA1P7,[23] POU5F1B,[24] FOXO3P,[25] and INTS6P1.[26] PTTG3P is a novel pseudogene, which has been found to be closely associated with the onset and progression of some types of human cancer, such as gastric cancer and hepatocellular carcinoma.[14,15] However, the expression pattern and potential roles of PTTG3P in breast cancer, as well as its underlying regulatory mechanisms, remain largely unknown.

In this study, we first determined the expression level of PTTG3P in breast cancer, based on bioinformatics analyses and experimental validation. The SAGE Digital Gene Expression Display showed no macroscopic differences in the expression of PTTG3P in the cancer and normal groups, partially because of the gene's extremely low overall expression. Oncomine analysis revealed that expression of PTTG3P in breast cancer tissues was significantly higher than that in normal tissues. qRT-PCR for clinical samples also demonstrated a high expression level of PTTG3P in breast cancer tissues when compared with their matched normal tissues. Moreover, a negative association between PTTG3P expression and ER and PR status was found. Conversely, basal-like status, TNBC status, NPI, or SBR grade was positively linked to PTTG3P expression. Subsequently, the Kaplan-Meier Plotter, UALCAN, and OncoLnc databases were used to study the prognostic role of PTTG3P in breast cancer, and the analytic results displayed that high expression of PTTG3P indicated poor RFS, OS, and DMFS. These findings suggest that increased expression of PTTG3P may be a promising prognostic biomarker in breast cancer.

Pseudogenes exert their functions in three levels, including DNA, RNA, and protein by a variety of mechanisms, involving gene conversion, homologous recombination, exonization, insertional mutations, and serving as antisense RNAs, endo-siRNAs, or competing endogenous RNAs.[4] To explore the potential regulatory mechanisms of PTTG3P, we first constructed a pseudogene-miRNA-mRNA interaction network. In this network, 12 potential miRNAs were identified as candidates for PTTG3P and 1,713 target genes for the identified

Figure 4. Construction of PTTG3P-miRNA-mRNA Network and Gene Ontology Functional Annotation and KEGG Pathway Enrichment Analysis for the Co-expressed Genes of PTTG3P

(A) The potential pseudogene PTTG3P-miRNA-mRNA regulatory network; (B) expression of miR-129-5p in breast cancer tissues and normal controls determined by starBase v3.0; (C) expression of miR-376c-3p in breast cancer tissues and normal controls determined by starBase v3.0; (D) expression of miR-383-5p in breast cancer tissues and normal controls determined by starBase v3.0; (E) the top 10 enriched biological process (BP) items for the co-expressed genes of PTTG3P; (F) the top 10 enriched cellular component (CC) items for the co-expressed genes of PTTG3P; (G) the top 10 enriched molecular function (MF) items for the co-expressed genes of PTTG3P; and (H) the top 10 enriched KEGG pathway items for the co-expressed genes of PTTG3P.

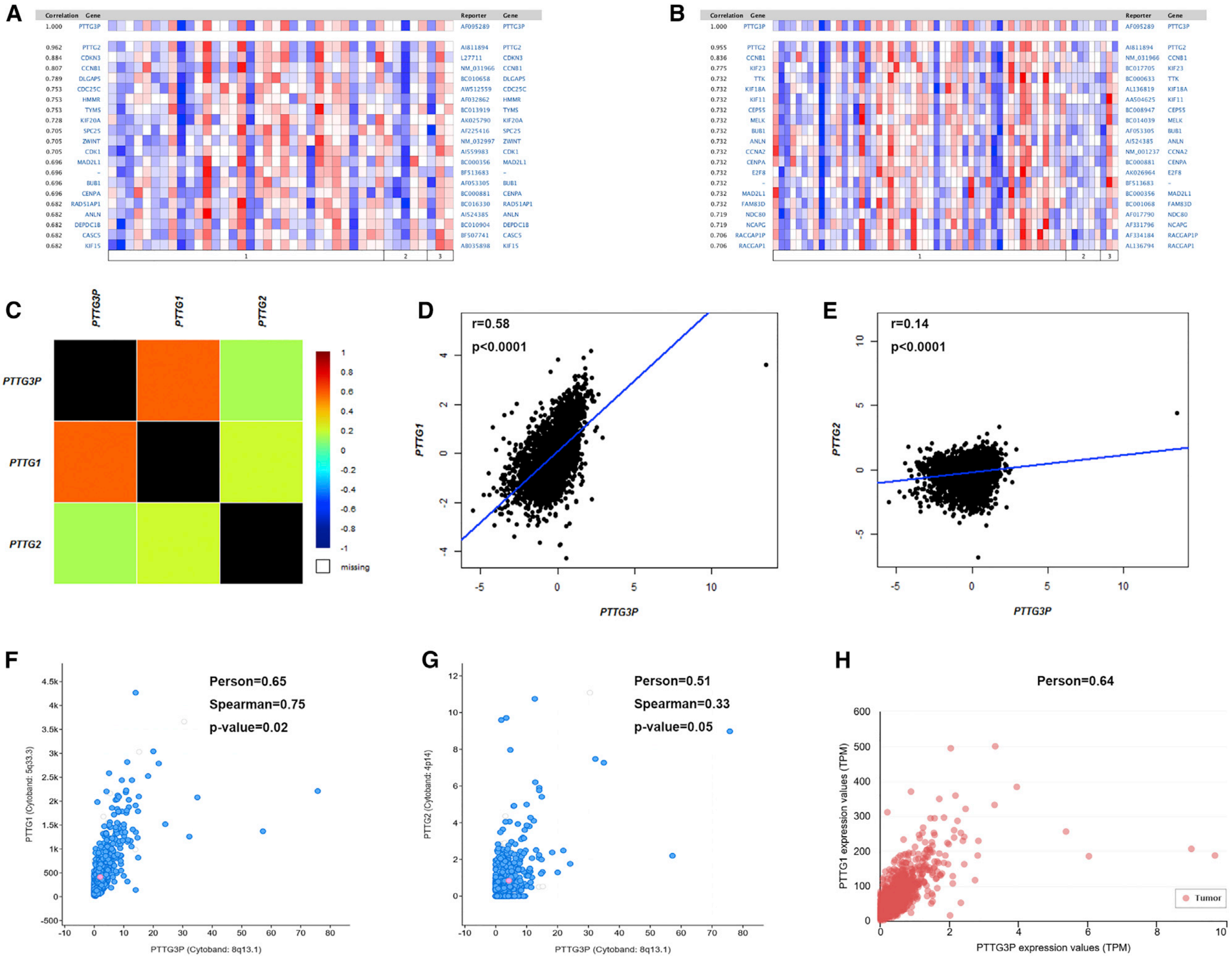

Figure 5. Expression Correlation of PTTG3 with Parental Genes Analyzed by Oncomine, bc-GenExMiner, cBioPortal, and UALCAN Databases

(A and B) PTTG3P co-expression of genes analyzed using Oncomine database; (C–E) expression relationship between PTTG3P and PTTG1/PTTG2 in breast cancer analyzed using bc-GenExMiner database; (F and G) expression correlation between PTTG3P and PTTG1/PTTG2 in breast cancer analyzed using cBioPortal database; and (H) expression correlation between PTTG3P and -1 in breast cancer analyzed using the UALCAN database.

miRNAs were finally predicted. Next, we further determined the expression levels of the 12 potential miRNAs in breast cancer and found that 3 of 12 (miR-129-5p, miR-376c-3p, and miR-383-5p) miRNAs were significantly downregulated in breast cancer. According to the ceRNA hypothesis, the three miRNAs may be three downstream miRNAs. Moreover, previous studies have confirmed that all three miRNAs function as tumor suppressors in various types of human cancers, including breast cancer.[27–31] Co-expression analysis may also provide critical clues for investigating the mechanisms of PTTG3P. UALCAN database analysis acquired 220 positively correlated genes. Functional annotation and KEGG pathway enrichment analysis showed that the co-expressed genes were mainly enriched in nuclear division, cell cycle, and oocyte meiosis. Therefore, we supposed that PTTG3P exerted its roles in cancer by these biological processes.

Numerous studies have suggested that pseudogenes can regulate their parental genes and thereby are involved in different cellular processes.[16] PTTG1 and -2 are two parental genes of PTTG3P. It has been well documented that PTTG1 and -2 act as two oncogenes in multiple human cancers.[12,32,33] We found that PTTG3P expression was significantly positively correlated with PTTG1 expression in the Oncomine, bc-GenExMiner, cBioPortal, and UALCAN databases, but was markedly associated with PTTG2 expression only in the Oncomine and bc-GenExMiner databases. The correlation of PTTG3P with PTTG1 or -2 was further assessed in clinical samples. The results revealed that PTTG3P correlated positively only with PTTG1. Previous studies have suggested that PTTG1 exerts oncogenic roles mainly by regulating the cell cycle.[15,33] These findings and the present results provide ample evidence that PTTG3P may promote breast cancer progression through modulation of cell

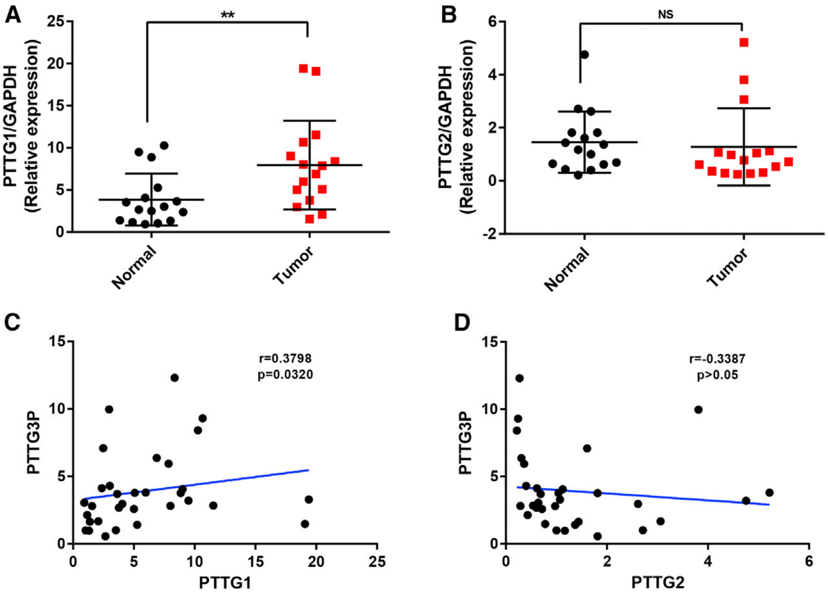

Figure 6. Expression of PTTG1/PTTG2 and Correlation between PTTG3P Expression and PTTG1/PTTG2 Expression in Clinical Breast Cancer Samples

(A) The level of PTTG1 was detected in 16 breast cancer tissues and paired non-tumor tissues by qRT-PCR; (B) the level of PTTG2 was detected in 16 breast cancer tissues and paired non-tumor tissues by qRT-PCR; (C) the correlation of PTTG3P with PTTG1 in the 32 clinical samples; (D) the correlation of PTTG3P with PTTG2 in the 32 clinical samples. Error bars represent SD. **p < 0.01; ns represents no significance.

cycle-associated processes by partially upregulating expression of its parental genes, especially PTTG1.

Altogether, based on bioinformatics analysis and experimental validation (Figure 7), we provided a reliable integrated analysis of the pseudogene PTTG3P expression pattern, prognostic value, and potential regulatory mechanisms in breast cancer. In this study, high expression of pseudogene PTTG3P in breast cancer was confirmed, and increased expression of PTTG3P indicated a poor prognosis of breast cancer. Furthermore, a potential pseudogene PTTG3P-miRNA-mRNA regulatory network was constructed, and the three most possible downstream miRNAs (miR-129-5p, miR-376c-3p, and miR-383-5p) of PTTG3P were identified. Our findings also supported that PTTG3P may upregulate PTTG1 expression, thus functioning as an oncogene in breast cancer. In summary, pseudogene PTTG3P may serve as a novel prognostic biomarker and a therapeutic target for patients with breast cancer. In the future, more laboratory experiments and clinical trials need to be performed to further validate the findings in this study.

MATERIALS AND METHODS

Expression Profile Analysis Using Serial Analysis of Gene Expression and Oncomine

The expression profile of pseudogene PTTG3P was analyzed with two online databases, including SAGE and Oncomine. First, all available published SAGE data were used to analyze PTTG3P expression in 23 normal human tissues and corresponding malignant counterparts, and PTTG3P expression profile was displayed using the SAGE Anatomic Viewer (http://cgap.nci.nih.gov/SAGE/AnatomicViewer/). We also determined the PTTG3P expression profile using Oncomine, which is a cancer microarray database and an integrated data-mining platform, to facilitate discovery from genome-wide expression analyses.[34] To date, the Oncomine database has 19 cancer types containing 92 datasets. Oncomine analysis of cancer versus normal samples in these 19 cancer types was conducted. $p < 0.05$, $|\text{fold change}| > 1.5$ and gene rank in the top 10% were set as the thresholds. We also conducted a meta-analysis of datasets in breast cancer. The Oncomine database was also used to perform a co-expression analysis of PTTG3P and other genes.

Breast Cancer Gene-Expression Miner

Breast cancer gene-expression miner (bc-GenExMiner), a statistical mining tool of published annotated genomic data, was introduced to assess the correlation of pseudogene PTTG3P expression level with specific clinicopathological features of breast cancer, including age, nodal status, hormonal receptors status (ER and PR), HER2, pathological subtype, NPI, and SBR grade.[35,36] bc-GenExMiner was also used to determine the expression correlation of PTTG3P with PTTG1 and 2. $p < 0.05$ was considered statistically significant.

Survival Analysis Using Kaplan-Meier Plotter, UALCAN, and OncoLnc Databases

The Kaplan-Meier Plotter (http://kmplot.com/analysis/), an online database established from gene expression data and survival information of cancer patients downloaded from the Gene Expression Omnibus (GEO) database, was used to evaluate the prognostic value of PTTG3P in breast cancer.[37] PTTG3P was first entered into the database. A Kaplan-Meier survival plot, hazards ratio (HR), 95% confidence interval (CI), and log rank p were directly determined and displayed on the webpage. A log rank $p < 0.05$ was considered statistically significant.

UALCAN (http://ualcan.path.uab.edu/index.html/) is a portal for facilitating tumor subgroup gene expression and survival analyses, providing easy access to publicly available cancer transcriptome data containing TCGA.[38] It was employed to evaluate the effect of PTTG3P on breast cancer patient survival. $p < 0.05$ was considered statistically significant.

OncoLnc (http://www.oncolnc.org/), a database for interactively investigating survival correlations and retrieving clinical data associated

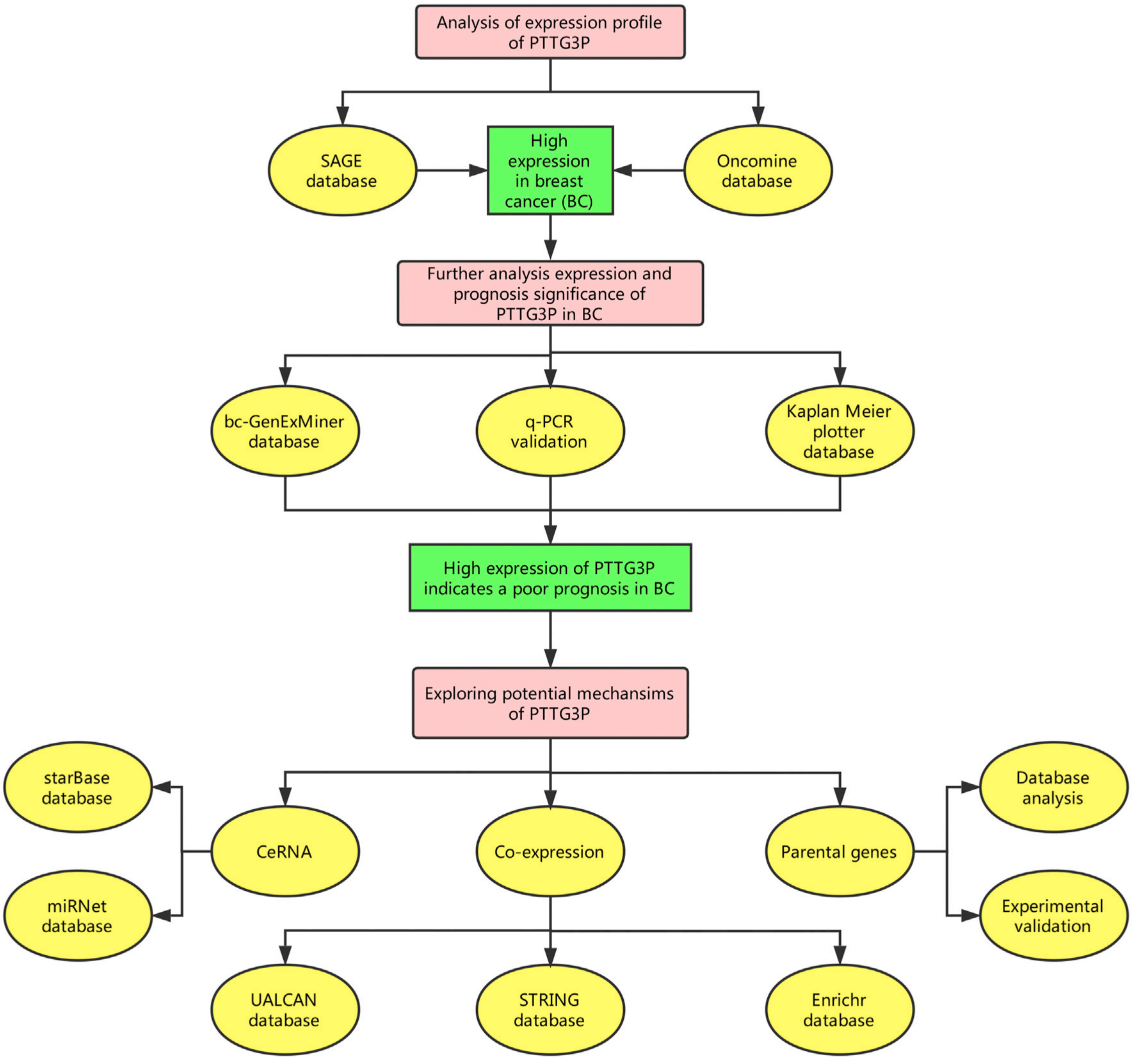

Figure 7. The Visual Flow-Process Diagram of This Study

with expression data for mRNAs, miRNAs, long non-coding RNAs, and pseudogenes, was also used to assess the prognostic value of PTTG3P in breast cancer.[6] $p < 0.05$ was considered statistically significant.

Construction of the Pseudogene-miRNA-mRNA Regulatory Network

The pseudogene PTTG3P-miRNA-mRNA regulatory network was constructed. First, starBase v3.0 (http://starbase.sysu.edu.cn/) was employed to identify potential miRNAs binding to pseudogene PTTG3P.[39] Subsequently, target genes for identified miRNAs were predicted using the miRNet database (https://www.mirnet.ca/), which is an easy-to-use tool for comprehensive statistical analysis and functional interpretation of data from miRNAs studies.[40] Expression levels of the potential miRNAs in breast cancer were also detected by starBase v3.0 (http://starbase.sysu.edu.cn/). $p < 0.05$ was considered statistically significant.

Co-expression Analysis

Those genes co-expressed with pseudogene PTTG3P were analyzed using UALCAN.[41] Genes positively correlated with PTTG3P in breast cancer were downloaded from UALCAN. Genes with extremely low expression (median transcripts per million < 0.5) were filtered out, and only genes with a Pearson correlation coefficient ≥ 0.3 were included.

Protein-Protein Interaction Network

Protein-protein interaction network for the co-expressed genes was established using STRING v10.5 (https://string-db.org/cgi/input.pl/), which is a database of predicted functional associations between

proteins.[42] The network displayed on the webpage was gathered into three clusters and then exported as a high-resolution bitmap.

Gene Ontology and KEGG Pathway Enrichment Analysis

The Enrichr database (http://amp.pharm.mssm.edu/Enrichr/) was employed to perform GO functional annotation and KEGG pathway enrichment analysis for these co-expressed genes.[43] GO functional analysis contained three categories, including BP, CC, and MF. The bar charts of GO and KEGG analysis for these co-expressed genes were directly downloaded from the webpage.

cBioPortal Database Analysis

In addition to using the Oncomine, bc-GenExMiner, and UALCAN databases mentioned above, we conducted a correlation analysis of pseudogene PTTG3P and its parental gene PTTG1 and -2 using the cBioPortal database (http://www.cbioportal.org/).[44] $p < 0.05$ was considered statistically significant.

Gathering of Clinical Samples and Ethics Statement

Altogether, 50 clinical breast cancer tissues and matched normal tissues were collected from the Zhejiang Cancer Hospital (Hangzhou, China). All the samples were kept in liquid nitrogen until the extraction of RNA. Written informed consent for the biological experiments was obtained from every patient involved in the study, and the study was approved by the Ethics Committee of the Zhejiang Cancer Hospital.

RNA Extraction and qRT-PCR Analysis

RNA extraction and qRT-PCR were performed as described previously.[38,45,46] The primers used in this study were purchased from Ribobio (Guangzhou, China) and are listed in Table S5.

Statistical Analysis

Each experiment was performed in triplicate and repeated at least three times. GraphPad Prism Software, Version 7, was introduced to conduct statistical analysis. The statistical significance of the differences between two groups was estimated by two-tailed Student's t test. Spearman correlation coefficients were calculated to evaluate the association between PTTG3P and -1 or -2.

SUPPLEMENTAL INFORMATION

Supplemental Information can be found online at https://doi.org/10.1016/j.omto.2019.03.006.

AUTHOR CONTRIBUTIONS

Conceptualization, W.L. and W.F.; methodology, W.L. and B.D.; software, W.L. and B.D.; writing of the original draft, W.L.; writing review and editing, W.L., B.D. and W.F.; and funding acquisition, W.F.

CONFLICTS OF INTEREST

The authors declare no competing interests.

ACKNOWLEDGMENTS

The authors would like to acknowledge financial support for this work from grants NNSF-81372462 and NNSF-81572987.

REFERENCES

1. Cui, X., Yi, Q., Jing, X., Huang, Y., Tian, J., Long, C., Xiang, Z., Tan, B., Li, Y., Zhu, J., et al. (2018). Mining Prognostic Significance of MEG3 in Human Breast Cancer Using Bioinformatics Analysis. Cell. Physiol. Biochem. *50*, 41–51.
2. DeSantis, C.E., Ma, J., Goding Sauer, A., Newman, L.A., and Jemal, A. (2017). Breast cancer statistics, 2017, racial disparity in mortality by state. CA Cancer J. Clin. *67*, 439–448.
3. Sasidharan, R., and Gerstein, M. (2008). Genomics: protein fossils live on as RNA. Nature *453*, 729–731.
4. Xiao-Jie, L., Ai-Mei, G., Li-Juan, J., and Jiang, X. (2015). Pseudogene in cancer: real functions and promising signature. J. Med. Genet. *52*, 17–24.
5. Chen, B., Wang, C., Zhang, J., Zhou, Y., Hu, W., and Guo, T. (2018). New insights into long noncoding RNAs and pseudogenes in prognosis of renal cell carcinoma. Cancer Cell Int. *18*, 157.
6. Saha, S.K., Jeong, Y., Cho, S., and Cho, S.G. (2018). Systematic expression alteration analysis of master reprogramming factor OCT4 and its three pseudogenes in human cancer and their prognostic outcomes. Sci. Rep. *8*, 14806.
7. Song, H., Yang, J., Zhang, Y., Zhou, J., Li, Y., and Hao, X. (2019). Integrated analysis of pseudogene RP11-564D11.3 expression and its potential roles in hepatocellular carcinoma. Epigenomics *11*, 267–280.
8. Chen, L., Puri, R., Lefkowitz, E.J., and Kakar, S.S. (2000). Identification of the human pituitary tumor transforming gene (hPTTG) family: molecular structure, expression, and chromosomal localization. Gene *248*, 41–50.
9. Repo, H., Gurvits, N., Löyttyniemi, E., Nykänen, M., Lintunen, M., Karra, H., Kurki, S., Kuopio, T., Talvinen, K., Söderström, M., and Kronqvist, P. (2017). PTTG1-interacting protein (PTTG1IP/PBF) predicts breast cancer survival. BMC Cancer *17*, 705.
10. Xiea, Y., and Wangb, R. (2016). Pttg1 Promotes Growth of Breast Cancer through P27 Nuclear Exclusion. Cell. Physiol. Biochem *38*, 393–400.
11. Mirandola, L., Figueroa, J.A., Phan, T.T., Grizzi, F., Kim, M., Rahman, R.L., Jenkins, M.R., Cobos, E., Jumper, C., Alalawi, R., and Chiriva-Internati, M. (2015). Novel antigens in non-small cell lung cancer: SP17, AKAP4, and PTTG1 are potential immunotherapeutic targets. Oncotarget *6*, 2812–2826.
12. Fu, D., Zhang, Y., and Cui, H. (2018). Long noncoding RNA CCAT2 is activated by E2F1 and exerts oncogenic properties by interacting with PTTG1 in pituitary adenomas. Am. J. Cancer Res. *8*, 245–255.
13. Feng, W., Xiaoyan, X., Shenglei, L., Hongtao, L., and Guozhong, J. (2017). PTTG1 cooperated with GLI1 leads to epithelial-mesenchymal transition in esophageal squamous cell cancer. Oncotarget *8*, 92388–92400.
14. Weng, W., Ni, S., Wang, Y., Xu, M., Zhang, Q., Yang, Y., Wu, Y., Xu, Q., Qi, P., Tan, C., et al. (2017). PTTG3P promotes gastric tumour cell proliferation and invasion and is an indicator of poor prognosis. J. Cell. Mol. Med. *21*, 3360–3371.
15. Huang, J.L., Cao, S.W., Ou, Q.S., Yang, B., Zheng, S.H., Tang, J., Chen, J., Hu, Y.W., Zheng, L., and Wang, Q. (2018). The long non-coding RNA PTTG3P promotes cell growth and metastasis via up-regulating PTTG1 and activating PI3K/AKT signaling in hepatocellular carcinoma. Mol. Cancer *17*, 93.
16. An, Y., Furber, K.L., and Ji, S. (2017). Pseudogenes regulate parental gene expression via ceRNA network. J. Cell. Mol. Med. *21*, 185–192.
17. Poliseno, L. (2012). Pseudogenes: newly discovered players in human cancer. Sci. Signal. *5*, re5.
18. De Martino, M., Forzati, F., Arra, C., Fusco, A., and Esposito, F. (2016). HMGA1-pseudogenes and cancer. Oncotarget *7*, 28724–28735.
19. Han, L., Yuan, Y., Zheng, S., Yang, Y., Li, J., Edgerton, M.E., Diao, L., Xu, Y., Verhaak, R.G.W., and Liang, H. (2014). The Pan-Cancer analysis of pseudogene expression reveals biologically and clinically relevant tumour subtypes. Nat. Commun. *5*, 3963.
20. Hu, X., Yang, L., and Mo, Y.Y. (2018). Role of Pseudogenes in Tumorigenesis. Cancers (Basel) *10*, 256.
21. Tutar, L., Özgür, A., and Tutar, Y. (2018). Involvement of miRNAs and Pseudogenes in Cancer. Methods Mol. Biol. *1699*, 45–66.
22. Yu, G., Yao, W., Gumireddy, K., Li, A., Wang, J., Xiao, W., Chen, K., Xiao, H., Li, H., Tang, K., et al. (2014). Pseudogene PTENP1 functions as a competing endogenous

RNA to suppress clear-cell renal cell carcinoma progression. Mol. Cancer Ther. *13*, 3086–3097.

23. Esposito, F., De Martino, M., D'Angelo, D., Mussnich, P., Raverot, G., Jaffrain-Rea, M.L., Fraggetta, F., Trouillas, J., and Fusco, A. (2015). HMGA1-pseudogene expression is induced in human pituitary tumors. Cell Cycle *14*, 1471–1475.
24. Hayashi, H., Arao, T., Togashi, Y., Kato, H., Fujita, Y., De Velasco, M.A., Kimura, H., Matsumoto, K., Tanaka, K., Okamoto, I., et al. (2015). The OCT4 pseudogene POU5F1B is amplified and promotes an aggressive phenotype in gastric cancer. Oncogene *34*, 199–208.
25. Yang, W., Du, W.W., Li, X., Yee, A.J., and Yang, B.B. (2016). Foxo3 activity promoted by non-coding effects of circular RNA and Foxo3 pseudogene in the inhibition of tumor growth and angiogenesis. Oncogene *35*, 3919–3931.
26. Peng, H., Ishida, M., Li, L., Saito, A., Kamiya, A., Hamilton, J.P., Fu, R., Olaru, A.V., An, F., Popescu, I., et al. (2015). Pseudogene INTS6P1 regulates its cognate gene INTS6 through competitive binding of miR-17-5p in hepatocellular carcinoma. Oncotarget *6*, 5666–5677.
27. Deng, Y., Xiong, Y., and Liu, Y. (2016). miR-376c inhibits cervical cancer cell proliferation and invasion by targeting BMI1. Int. J. Exp. Pathol. *97*, 257–265.
28. Jiang, J., Xie, C., Liu, Y., Shi, Q., and Chen, Y. (2019). Up-regulation of miR-383-5p suppresses proliferation and enhances chemosensitivity in ovarian cancer cells by targeting TRIM27. Biomed. Pharmacother *109*, 595–601.
29. Meng, R., Fang, J., Yu, Y., Hou, L.K., Chi, J.R., Chen, A.X., Zhao, Y., and Cao, X.C. (2018). miR-129-5p suppresses breast cancer proliferation by targeting CBX4. Neoplasma *65*, 572–578.
30. Xiao, G., Li, X., Li, G., Zhang, B., Xu, C., Qin, S., Du, N., Wang, J., Tang, S.C., Zhang, J., et al. (2017). MiR-129 blocks estrogen induction of NOTCH signaling activity in breast cancer stem-like cells. Oncotarget *8*, 103261–103273.
31. Zeng, H., Wang, L., Wang, J., Chen, T., Li, H., Zhang, K., Chen, J., Zhen, S., Tuluhong, D., Li, J., and Wang, S. (2018). microRNA-129-5p suppresses Adriamycin resistance in breast cancer by targeting SOX2. Arch. Biochem. Biophys. *651*, 52–60.
32. Guo, Y., Shao, Y., Chen, J., Xu, S., Zhang, X., and Liu, H. (2016). Expression of pituitary tumor-transforming 2 in human glioblastoma cell lines and its role in glioblastoma tumorigenesis. Exp. Ther. Med. *11*, 1847–1852.
33. Zhi, T., Jiang, K., Xu, X., Yu, T., Wu, W., Nie, E., Zhou, X., Jin, X., Zhang, J., Wang, Y., and Liu, N. (2017). MicroRNA-520d-5p inhibits human glioma cell proliferation and induces cell cycle arrest by directly targeting PTTG1. Am. J. Transl. Res. *9*, 4872–4887.
34. Rhodes, D.R., Yu, J., Shanker, K., Deshpande, N., Varambally, R., Ghosh, D., Barrette, T., Pandey, A., and Chinnaiyan, A.M. (2004). ONCOMINE: a cancer microarray database and integrated data-mining platform. Neoplasia *6*, 1–6.
35. Jézéquel, P., Campone, M., Gouraud, W., Guérin-Charbonnel, C., Leux, C., Ricolleau, G., and Campion, L. (2012). bc-GenExMiner: an easy-to-use online platform for gene prognostic analyses in breast cancer. Breast Cancer Res. Treat. *131*, 765–775.
36. Jezequel, P., Frenel, J.S., Campion, L., Guerin-Charbonnel, C., Gouraud, W., Ricolleau, G., and Campone, M. (2013). bc-GenExMiner 3.0: new mining module computes breast cancer gene expression correlation analyses. Database *2013*, bas060.
37. Győrffy, B., Surowiak, P., Budczies, J., and Lánczky, A. (2013). Online survival analysis software to assess the prognostic value of biomarkers using transcriptomic data in non-small-cell lung cancer. PLoS ONE *8*, e82241.
38. Lou, W., Chen, J., Ding, B., Chen, D., Zheng, H., Jiang, D., Xu, L., Bao, C., Cao, G., and Fan, W. (2018). Identification of invasion-metastasis-associated microRNAs in hepatocellular carcinoma based on bioinformatic analysis and experimental validation. J. Transl. Med. *16*, 266.
39. Li, J.H., Liu, S., Zhou, H., Qu, L.H., and Yang, J.H. (2014). starBase v2.0: decoding miRNA-ceRNA, miRNA-ncRNA and protein-RNA interaction networks from large-scale CLIP-Seq data. Nucleic Acids Res. *42*, D92–D97.
40. Fan, Y., Siklenka, K., Arora, S.K., Ribeiro, P., Kimmins, S., and Xia, J. (2016). miRNet: dissecting miRNA-target interactions and functional associations through network-based visual analysis. Nucleic Acids Res. *44*, W135–W141.
41. Chandrashekar, D.S., Bashel, B., Balasubramanya, S.A.H., Creighton, C.J., Ponce-Rodriguez, I., Chakravarthi, B.V.S.K., and Varambally, S. (2017). UALCAN: A Portal for Facilitating Tumor Subgroup Gene Expression and Survival Analyses. Neoplasia *19*, 649–658.
42. Szklarczyk, D., Franceschini, A., Kuhn, M., Simonovic, M., Roth, A., Minguez, P., Doerks, T., Stark, M., Muller, J., Bork, P., et al. (2011). The STRING database in 2011: functional interaction networks of proteins, globally integrated and scored. Nucleic Acids Res. *39*, D561–D568.
43. Kuleshov, M.V., Jones, M.R., Rouillard, A.D., Fernandez, N.F., Duan, Q., Wang, Z., Koplev, S., Jenkins, S.L., Jagodnik, K.M., Lachmann, A., et al. (2016). Enrichr: a comprehensive gene set enrichment analysis web server 2016 update. Nucleic Acids Res. *44*, W90–W97.
44. Cerami, E., Gao, J., Dogrusoz, U., Gross, B.E., Sumer, S.O., Aksoy, B.A., Jacobsen, A., Byrne, C.J., Heuer, M.L., Larsson, E., et al. (2012). The cBio cancer genomics portal: an open platform for exploring multidimensional cancer genomics data. Cancer Discov. *2*, 401–404.
45. Lou, W., Liu, J., Ding, B., Chen, D., Xu, L., Ding, J., Jiang, D., Zhou, L., Zheng, S., and Fan, W. (2019). Identification of potential miRNA-mRNA regulatory network contributing to pathogenesis of HBV-related HCC. J. Transl. Med. *17*, 7.
46. Lou, W., Liu, J., Ding, B., Xu, L., and Fan, W. (2018). Identification of chemoresistance-associated miRNAs in breast cancer. Cancer Manag. Res. *10*, 4747–4757.

OMTO, Volume *14*

Supplemental Information

High Expression of Pseudogene PTTG3P Indicates a Poor Prognosis in Human Breast Cancer

Weiyang Lou, Bisha Ding, and Weimin Fan

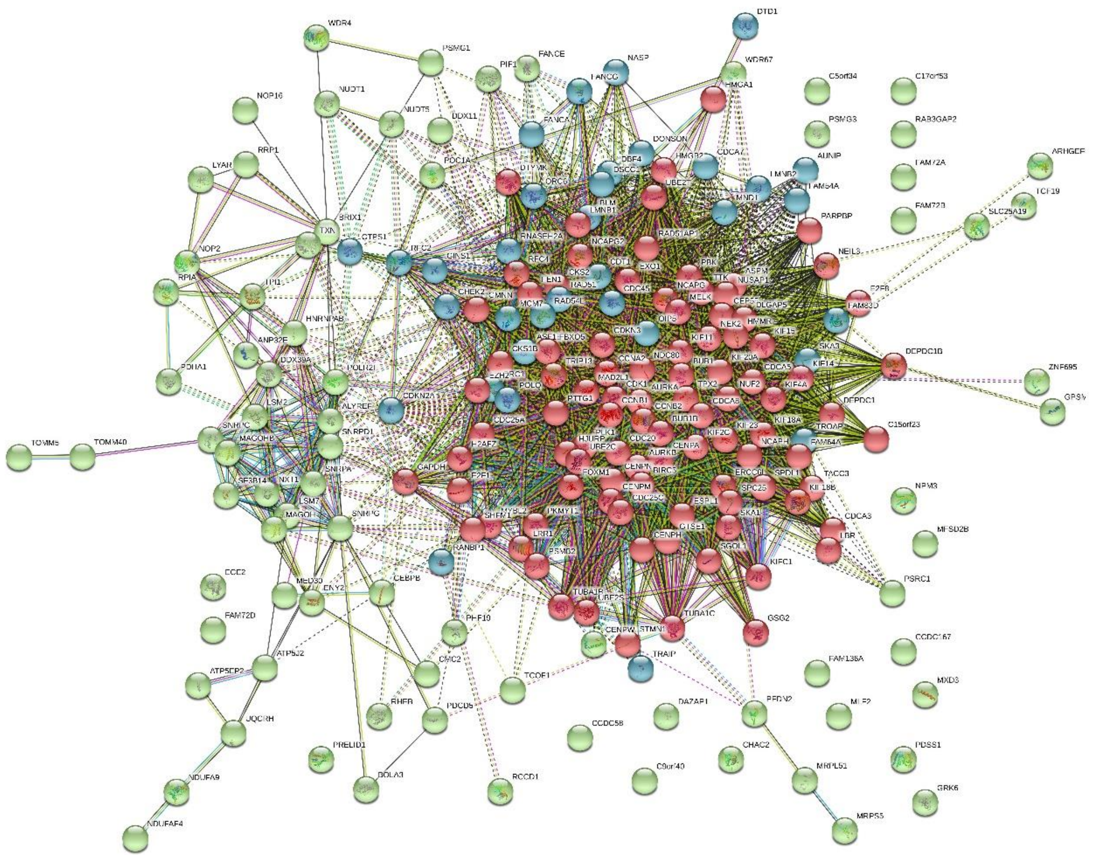

Figure S1. The protein-protein interaction network of the co-expressed genes of PTTG3P constructed by STRING V10.5.

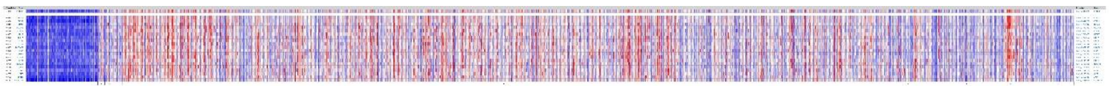

Figure S2. PTTG3P co-expression of genes analyzed using Oncomine database.

Table S1. The relationship between PTTG3P expression and different clinicopathological parameters of breast carcinoma.

Variables	Number	PTTG3P<br>mRNA expression	P-value
Age			0.4159
≤ 51	1048	-	
> 51	1449	-	
Nodal Statas			0.1266
-	2041	-	
+	1122	-	
ER			**<0.0001**
-	1142	Up	
+	3162	-	
PR			**<0.0001**
-	700	Up	

+	1136	-	
HER2			0.1423
-	1195	-	
+	166	-	
Basal-like Status			**<0.0001**
Not	3349	-	
Basal-like	916	Up	
Triple-negative Status			**<0.0001**
Not	3299	-	
TNBC	293	Up	
NPI			**<0.0001**
1	624	-	
2	486	Up	
3	12	Up	
SBR			**<0.0001**
1	434	-	
2	1128	Up	
3	1040	Up	

Table S2. The potential miRNA-target gene pairs predicted by miRNet.

miRNA	Gene
hsa-mir-129-5p	HBS1L
hsa-mir-129-5p	SH3BP2
hsa-mir-129-5p	SIX1
hsa-mir-129-5p	SLC4A1
hsa-mir-129-5p	SLC16A1
hsa-mir-129-5p	SUMO2
hsa-mir-129-5p	SNX2
hsa-mir-129-5p	SOX4
hsa-mir-129-5p	SOX12
hsa-mir-129-5p	SP1
hsa-mir-129-5p	STAR
hsa-mir-129-5p	ADAM17
hsa-mir-129-5p	TAGLN
hsa-mir-129-5p	TAPBP
hsa-mir-129-5p	TDGF1P3
hsa-mir-129-5p	TEAD3
hsa-mir-129-5p	TERF2
hsa-mir-129-5p	NR2F2
hsa-mir-129-5p	THRB
hsa-mir-129-5p	TRAF3
hsa-mir-129-5p	TRPS1
hsa-mir-129-5p	TWIST1
hsa-mir-129-5p	UBE2G2

hsa-mir-129-5p	UBE2V2
hsa-mir-129-5p	VIM
hsa-mir-129-5p	VLDLR
hsa-mir-129-5p	WEE1
hsa-mir-129-5p	YES1
hsa-mir-129-5p	YY1
hsa-mir-129-5p	ZNF708
hsa-mir-129-5p	ZNF154
hsa-mir-129-5p	ZNF215
hsa-mir-129-5p	ZNF226
hsa-mir-129-5p	LAPTM5
hsa-mir-129-5p	SLBP
hsa-mir-129-5p	BRD3
hsa-mir-129-5p	SLC25A16
hsa-mir-129-5p	ADAM12
hsa-mir-129-5p	RND2
hsa-mir-129-5p	EOMES
hsa-mir-129-5p	HIST1H4C
hsa-mir-129-5p	TTF2
hsa-mir-129-5p	SORBS2
hsa-mir-129-5p	CBX4
hsa-mir-129-5p	AKR7A2
hsa-mir-129-5p	TP63
hsa-mir-129-5p	NCOA1
hsa-mir-129-5p	BECN1
hsa-mir-129-5p	NOL4
hsa-mir-129-5p	HIST1H2BJ
hsa-mir-129-5p	PIAS2
hsa-mir-129-5p	MAP3K13
hsa-mir-129-5p	VAPA
hsa-mir-129-5p	TIAF1
hsa-mir-129-5p	LRAT
hsa-mir-129-5p	TBRG4
hsa-mir-129-5p	RPS6KA5
hsa-mir-129-5p	PIWIL1
hsa-mir-129-5p	SNAP29
hsa-mir-129-5p	RAB9A
hsa-mir-129-5p	KIF3B
hsa-mir-129-5p	CIAO1
hsa-mir-129-5p	GRAP2
hsa-mir-129-5p	ZRANB2
hsa-mir-129-5p	CRIPT
hsa-mir-129-5p	KCNK6
hsa-mir-129-5p	HOMER1

hsa-mir-129-5p	RASAL2
hsa-mir-129-5p	AKAP5
hsa-mir-129-5p	ADAMTS4
hsa-mir-129-5p	SPTLC2
hsa-mir-129-5p	EI24
hsa-mir-129-5p	H6PD
hsa-mir-129-5p	GABBR2
hsa-mir-129-5p	CLOCK
hsa-mir-129-5p	RNF14
hsa-mir-129-5p	ABCG1
hsa-mir-129-5p	USP6NL
hsa-mir-129-5p	SEMA3E
hsa-mir-129-5p	KIAA0408
hsa-mir-129-5p	CCP110
hsa-mir-129-5p	JAKMIP2
hsa-mir-129-5p	SEC24D
hsa-mir-129-5p	ZC3H11A
hsa-mir-129-5p	PAN2
hsa-mir-129-5p	ZBTB5
hsa-mir-129-5p	CCS
hsa-mir-129-5p	SH2B3
hsa-mir-129-5p	HMGXB4
hsa-mir-129-5p	ABCC5
hsa-mir-129-5p	ACTR1A
hsa-mir-129-5p	TRIM13
hsa-mir-129-5p	CALCOCO2
hsa-mir-129-5p	SF3A1
hsa-mir-129-5p	MCRS1
hsa-mir-129-5p	FST
hsa-mir-129-5p	TRIM38
hsa-mir-129-5p	ANP32B
hsa-mir-129-5p	RABAC1
hsa-mir-129-5p	COLEC10
hsa-mir-129-5p	CDC42EP3
hsa-mir-129-5p	IGF2BP3
hsa-mir-129-5p	IGF2BP2
hsa-mir-129-5p	CD226
hsa-mir-129-5p	DCTN6
hsa-mir-129-5p	TNFSF13B
hsa-mir-129-5p	GMEB1
hsa-mir-129-5p	UGT2B11
hsa-mir-129-5p	POLQ
hsa-mir-129-5p	MAP3K2
hsa-mir-129-5p	ABL2

hsa-mir-129-5p	ADD3
hsa-mir-129-5p	AK1
hsa-mir-129-5p	APC
hsa-mir-129-5p	XIAP
hsa-mir-129-5p	ARSD
hsa-mir-129-5p	ARSE
hsa-mir-129-5p	ZFHX3
hsa-mir-129-5p	ATRX
hsa-mir-129-5p	AZF1
hsa-mir-129-5p	BCL7A
hsa-mir-129-5p	BDKRB2
hsa-mir-129-5p	BID
hsa-mir-129-5p	PRDM1
hsa-mir-129-5p	BMPR2
hsa-mir-129-5p	C1S
hsa-mir-129-5p	CACNG1
hsa-mir-129-5p	CALM1
hsa-mir-129-5p	CBS
hsa-mir-129-5p	CDH1
hsa-mir-129-5p	CDK6
hsa-mir-129-5p	CHRM2
hsa-mir-129-5p	AP1S1
hsa-mir-129-5p	CCR4
hsa-mir-129-5p	CCR6
hsa-mir-129-5p	COL1A1
hsa-mir-129-5p	COL9A2
hsa-mir-129-5p	KLF6
hsa-mir-129-5p	COX6B1
hsa-mir-129-5p	CPE
hsa-mir-129-5p	CPT1A
hsa-mir-129-5p	HAPLN1
hsa-mir-129-5p	MAPK14
hsa-mir-129-5p	VCAN
hsa-mir-129-5p	CTNS
hsa-mir-129-5p	DAB2
hsa-mir-129-5p	DARS
hsa-mir-129-5p	DDX3X
hsa-mir-129-5p	DMXL1
hsa-mir-129-5p	TIMM8A
hsa-mir-129-5p	DNAH9
hsa-mir-129-5p	ARID3A
hsa-mir-129-5p	DSG3
hsa-mir-129-5p	DUSP1
hsa-mir-129-5p	EBF1

hsa-mir-129-5p	EFNB1
hsa-mir-129-5p	EIF1AX
hsa-mir-129-5p	EMP2
hsa-mir-129-5p	EP300
hsa-mir-129-5p	EPB41L1
hsa-mir-129-5p	EPB42
hsa-mir-129-5p	EPS8
hsa-mir-129-5p	ESD
hsa-mir-129-5p	ESR1
hsa-mir-129-5p	ETV6
hsa-mir-129-5p	ACSL4
hsa-mir-129-5p	FMR1
hsa-mir-129-5p	FRK
hsa-mir-129-5p	KDSR
hsa-mir-129-5p	GABRB1
hsa-mir-129-5p	GALNT1
hsa-mir-129-5p	GDNF
hsa-mir-129-5p	GFPT1
hsa-mir-129-5p	GLUL
hsa-mir-129-5p	GM2A
hsa-mir-129-5p	GNAL
hsa-mir-129-5p	GNAQ
hsa-mir-129-5p	GRM3
hsa-mir-129-5p	GTF2H1
hsa-mir-129-5p	HMGB1
hsa-mir-129-5p	HNF4G
hsa-mir-129-5p	HNRNPA1
hsa-mir-129-5p	HOXD11
hsa-mir-129-5p	SP110
hsa-mir-129-5p	IGF1
hsa-mir-129-5p	IL10RB
hsa-mir-129-5p	IL12RB2
hsa-mir-129-5p	IMPA2
hsa-mir-129-5p	IREB2
hsa-mir-129-5p	CD82
hsa-mir-129-5p	KCNJ3
hsa-mir-129-5p	KCNJ6
hsa-mir-129-5p	L1CAM
hsa-mir-129-5p	LIMS1
hsa-mir-129-5p	FADS1
hsa-mir-129-5p	LPP
hsa-mir-129-5p	CAPRIN1
hsa-mir-129-5p	MBD1
hsa-mir-129-5p	MBL2

hsa-mir-129-5p	MCM4
hsa-mir-129-5p	MLLT6
hsa-mir-129-5p	MYO10
hsa-mir-129-5p	NDUFV3
hsa-mir-129-5p	NEUROD2
hsa-mir-129-5p	NFATC2
hsa-mir-129-5p	NFIB
hsa-mir-129-5p	NFRKB
hsa-mir-129-5p	NOTCH1
hsa-mir-129-5p	NPTX1
hsa-mir-129-5p	NR4A2
hsa-mir-129-5p	OMD
hsa-mir-129-5p	PAX7
hsa-mir-129-5p	PCK1
hsa-mir-129-5p	PCM1
hsa-mir-129-5p	PDE3A
hsa-mir-129-5p	PDGFRA
hsa-mir-129-5p	PDPK1
hsa-mir-129-5p	ABCB1
hsa-mir-129-5p	PIK3C3
hsa-mir-129-5p	PITX1
hsa-mir-129-5p	PLSCR1
hsa-mir-129-5p	POLR2D
hsa-mir-129-5p	POU2F2
hsa-mir-129-5p	POU3F1
hsa-mir-129-5p	PPARA
hsa-mir-129-5p	PRKCD
hsa-mir-129-5p	MAPK1
hsa-mir-129-5p	MAP2K2
hsa-mir-129-5p	PRKX
hsa-mir-129-5p	PSMB9
hsa-mir-129-5p	PTMA
hsa-mir-129-5p	PTPN2
hsa-mir-129-5p	PTPN14
hsa-mir-129-5p	PVR
hsa-mir-129-5p	RAB13
hsa-mir-129-5p	RAB27A
hsa-mir-129-5p	RANGAP1
hsa-mir-129-5p	RBBP4
hsa-mir-129-5p	RET
hsa-mir-129-5p	RGS16
hsa-mir-129-5p	RORA
hsa-mir-129-5p	RPS23
hsa-mir-129-5p	SATB1

hsa-mir-129-5p	SCN2B	
hsa-mir-129-5p	LMBRD2	
hsa-mir-129-5p	TIMM50	
hsa-mir-129-5p	FOXP2	
hsa-mir-129-5p	SYAP1	
hsa-mir-129-5p	TP53INP1	
hsa-mir-129-5p	CEP41	
hsa-mir-129-5p	GLCCI1	
hsa-mir-129-5p	LACTB	
hsa-mir-129-5p	GALNT13	
hsa-mir-129-5p	OSBPL10	
hsa-mir-129-5p		3-Mar
hsa-mir-129-5p	ZNF554	
hsa-mir-129-5p	WDR92	
hsa-mir-129-5p	FAM210B	
hsa-mir-129-5p	GINM1	
hsa-mir-129-5p	EXOC3-AS1	
hsa-mir-129-5p	SNAP47	
hsa-mir-129-5p	LEAP2	
hsa-mir-129-5p	DEFB118	
hsa-mir-129-5p	EXOSC6	
hsa-mir-129-5p	FGD4	
hsa-mir-129-5p	ANKRD9	
hsa-mir-129-5p	ISCA2	
hsa-mir-129-5p	FSD2	
hsa-mir-129-5p	SGK494	
hsa-mir-129-5p	ZNF440	
hsa-mir-129-5p	SWSAP1	
hsa-mir-129-5p	ZNF573	
hsa-mir-129-5p	TRABD2A	
hsa-mir-129-5p	TYW5	
hsa-mir-129-5p	GALM	
hsa-mir-129-5p	OCIAD2	
hsa-mir-129-5p	FAM241A	
hsa-mir-129-5p	RAET1E	
hsa-mir-129-5p	MPLKIP	
hsa-mir-129-5p	UNC5D	
hsa-mir-129-5p	SLITRK4	
hsa-mir-129-5p	OTUD6A	
hsa-mir-129-5p	SPIN4	
hsa-mir-129-5p	ASB8	
hsa-mir-129-5p	BRI3BP	
hsa-mir-129-5p	UBE2F	
hsa-mir-129-5p	WFDC6	

hsa-mir-129-5p	SESN3
hsa-mir-129-5p	TMEM120B
hsa-mir-129-5p	HNRNPA1L2
hsa-mir-129-5p	CMTM4
hsa-mir-129-5p	WIPF2
hsa-mir-129-5p	CLDN19
hsa-mir-129-5p	BTBD19
hsa-mir-129-5p	TTL
hsa-mir-129-5p	CKAP2L
hsa-mir-129-5p	C2orf15
hsa-mir-129-5p	GPR155
hsa-mir-129-5p	DTX3L
hsa-mir-129-5p	PPM1K
hsa-mir-129-5p	SLC38A9
hsa-mir-129-5p	SRFBP1
hsa-mir-129-5p	PRELID2
hsa-mir-129-5p	RUNDC3B
hsa-mir-129-5p	METTL27
hsa-mir-129-5p	TTC39B
hsa-mir-129-5p	ZDHHC15
hsa-mir-129-5p	ZNF519
hsa-mir-129-5p	KANK4
hsa-mir-129-5p	PARP15
hsa-mir-129-5p	FUT11
hsa-mir-129-5p	ADAMTS18
hsa-mir-129-5p	ZNF384
hsa-mir-129-5p	AGO3
hsa-mir-129-5p	AGO4
hsa-mir-129-5p	VSTM4
hsa-mir-129-5p	IBA57
hsa-mir-129-5p	PDE12
hsa-mir-129-5p	LRRC55
hsa-mir-129-5p	ZNF25
hsa-mir-129-5p	FAM124A
hsa-mir-129-5p	HNRNPA3
hsa-mir-129-5p	C10orf111
hsa-mir-129-5p	C6orf223
hsa-mir-129-5p	OARD1
hsa-mir-129-5p	ZNRF2
hsa-mir-129-5p	MCOLN2
hsa-mir-129-5p	TMEM151A
hsa-mir-129-5p	ANKS4B
hsa-mir-129-5p	MAGI3
hsa-mir-129-5p	AUTS8

hsa-mir-129-5p	KCNRG	
hsa-mir-129-5p	ZFP82	
hsa-mir-129-5p	NWD1	
hsa-mir-129-5p	SEC14L4	
hsa-mir-129-5p	RNF149	
hsa-mir-129-5p	PRRT3	
hsa-mir-129-5p	CEP57L1	
hsa-mir-129-5p	SCAI	
hsa-mir-129-5p	POTED	
hsa-mir-129-5p	MMAB	
hsa-mir-129-5p	CYP27C1	
hsa-mir-129-5p	ZDHHC21	
hsa-mir-129-5p		14-Sep
hsa-mir-129-5p	FAM71F2	
hsa-mir-129-5p	ZNF429	
hsa-mir-129-5p	ZNF699	
hsa-mir-129-5p	TMEM233	
hsa-mir-129-5p	LINC01551	
hsa-mir-129-5p	PLEKHM3	
hsa-mir-129-5p	ONECUT3	
hsa-mir-129-5p	ZNF793	
hsa-mir-129-5p	C7orf65	
hsa-mir-129-5p	YY2	
hsa-mir-129-5p	SRCAP	
hsa-mir-129-5p	RAB10	
hsa-mir-129-5p	SF3A3	
hsa-mir-129-5p	PNRC1	
hsa-mir-129-5p	ACOT2	
hsa-mir-129-5p	CKAP4	
hsa-mir-129-5p	CCNI	
hsa-mir-129-5p	FGFR1OP	
hsa-mir-129-5p	PLA2G16	
hsa-mir-129-5p	NUDT3	
hsa-mir-129-5p	FAM107A	
hsa-mir-129-5p	ZNF277	
hsa-mir-129-5p	SLC2A6	
hsa-mir-129-5p	AKAP10	
hsa-mir-129-5p	DUSP10	
hsa-mir-129-5p	PXMP4	
hsa-mir-129-5p	IKZF3	
hsa-mir-129-5p	NTNG1	
hsa-mir-129-5p	FNDC3A	
hsa-mir-129-5p	MLXIP	
hsa-mir-129-5p	MON1B	

hsa-mir-129-5p	DIS3
hsa-mir-129-5p	EPN2
hsa-mir-129-5p	IGSF9B
hsa-mir-129-5p	EXPH5
hsa-mir-129-5p	TNRC6B
hsa-mir-129-5p	RAD54L2
hsa-mir-129-5p	NUP205
hsa-mir-129-5p	TTLL12
hsa-mir-129-5p	METAP1
hsa-mir-129-5p	LPIN1
hsa-mir-129-5p	ARL6IP1
hsa-mir-129-5p	CAMTA1
hsa-mir-129-5p	ICOSLG
hsa-mir-129-5p	KIAA0930
hsa-mir-129-5p	TTC28
hsa-mir-129-5p	DPY19L1
hsa-mir-129-5p	RYBP
hsa-mir-129-5p	SLC44A1
hsa-mir-129-5p	CBX6
hsa-mir-129-5p	POFUT1
hsa-mir-129-5p	KAT6B
hsa-mir-129-5p	ACAP2
hsa-mir-129-5p	ADAT1
hsa-mir-129-5p	NUP62
hsa-mir-129-5p	TMEM245
hsa-mir-129-5p	HYPK
hsa-mir-129-5p	MTO1
hsa-mir-129-5p	FBXL2
hsa-mir-129-5p	TECPR1
hsa-mir-129-5p	LETMD1
hsa-mir-129-5p	RTTN
hsa-mir-129-5p	NGDN
hsa-mir-129-5p	TKFC
hsa-mir-129-5p	NSMF
hsa-mir-129-5p	ZNF500
hsa-mir-129-5p	TIAM2
hsa-mir-129-5p	TIMM8B
hsa-mir-129-5p	OSTF1
hsa-mir-129-5p	ZNF285
hsa-mir-129-5p	RNF11
hsa-mir-129-5p	FAM184B
hsa-mir-129-5p	SIGLEC9
hsa-mir-129-5p	RPS6KA6
hsa-mir-129-5p	P2RY10

hsa-mir-129-5p	RRP7A
hsa-mir-129-5p	POLL
hsa-mir-129-5p	APOBEC3C
hsa-mir-129-5p	OSTM1
hsa-mir-129-5p	SPCS1
hsa-mir-129-5p	DNAJC15
hsa-mir-129-5p	CFAP20
hsa-mir-129-5p	PRICKLE4
hsa-mir-129-5p	STRN3
hsa-mir-129-5p	KCNIP3
hsa-mir-129-5p	ST8SIA3
hsa-mir-129-5p	YARS2
hsa-mir-129-5p	MEMO1
hsa-mir-129-5p	MRPL4
hsa-mir-129-5p	KLHL5
hsa-mir-129-5p	ABHD5
hsa-mir-129-5p	RDH11
hsa-mir-129-5p	HSD17B12
hsa-mir-129-5p	PLEKHO1
hsa-mir-129-5p	GLTP
hsa-mir-129-5p	CRIM1
hsa-mir-129-5p	PHF21A
hsa-mir-129-5p	TAOK3
hsa-mir-129-5p	UFM1
hsa-mir-129-5p	MRPS23
hsa-mir-129-5p	CHMP3
hsa-mir-129-5p	LSM8
hsa-mir-129-5p	EMCN
hsa-mir-129-5p	ATP8A2
hsa-mir-129-5p	WNT4
hsa-mir-129-5p	RBM47
hsa-mir-129-5p	GNL3L
hsa-mir-129-5p	RSBN1
hsa-mir-129-5p	DYM
hsa-mir-129-5p	TRPM7
hsa-mir-129-5p	GIN1
hsa-mir-129-5p	BIVM
hsa-mir-129-5p	PGPEP1
hsa-mir-129-5p	CNTLN
hsa-mir-129-5p	MTMR10
hsa-mir-129-5p	LAX1
hsa-mir-129-5p	TTC19
hsa-mir-129-5p	TRMT10C
hsa-mir-129-5p	RNF125

hsa-mir-129-5p	PIGX
hsa-mir-129-5p	SLC35F6
hsa-mir-129-5p	TTC38
hsa-mir-129-5p	PHIP
hsa-mir-129-5p	PTCD3
hsa-mir-129-5p	TMEM51
hsa-mir-129-5p	FIGN
hsa-mir-129-5p	THAP1
hsa-mir-129-5p	LINS1
hsa-mir-129-5p	RIF1
hsa-mir-129-5p	SBNO1
hsa-mir-129-5p	UBA6
hsa-mir-129-5p	ELP2
hsa-mir-129-5p	CHDH
hsa-mir-129-5p	DHTKD1
hsa-mir-129-5p	FEM1A
hsa-mir-129-5p	SVOP
hsa-mir-129-5p	SLC30A10
hsa-mir-129-5p	SOX6
hsa-mir-129-5p	CDV3
hsa-mir-129-5p	LRRC40
hsa-mir-129-5p	DENND4C
hsa-mir-129-5p	LUC7L
hsa-mir-129-5p	TMEM30A
hsa-mir-129-5p	FGD6
hsa-mir-129-5p	DCP1A
hsa-mir-129-5p	CENPN
hsa-mir-129-5p	CISD1
hsa-mir-129-5p	C21orf62
hsa-mir-129-5p	CTNNBL1
hsa-mir-129-5p	PARD3
hsa-mir-129-5p	SAR1A
hsa-mir-129-5p	LHX9
hsa-mir-129-5p	YAE1D1
hsa-mir-129-5p	LYRM4
hsa-mir-129-5p	MAN1C1
hsa-mir-129-5p	PAK5
hsa-mir-129-5p	NIPAL3
hsa-mir-129-5p	ERGIC1
hsa-mir-129-5p	SMAGP
hsa-mir-129-5p	GJD2
hsa-mir-129-5p	LRTM1
hsa-mir-129-5p	AS3MT
hsa-mir-129-5p	ISY1

hsa-mir-129-5p	HEG1
hsa-mir-129-5p	MTA3
hsa-mir-129-5p	PCDH19
hsa-mir-129-5p	KCTD16
hsa-mir-129-5p	TAOK1
hsa-mir-129-5p	SEMA6A
hsa-mir-129-5p	CEP126
hsa-mir-129-5p	FNIP2
hsa-mir-129-5p	SLAIN2
hsa-mir-129-5p	POGK
hsa-mir-129-5p	USP28
hsa-mir-129-5p	KIAA1549
hsa-mir-129-5p	ZFP14
hsa-mir-129-5p	GATAD1
hsa-mir-129-5p	ZNF410
hsa-mir-129-5p	ZNF462
hsa-mir-129-5p	PBOV1
hsa-mir-129-5p	ANKEF1
hsa-mir-129-5p	RBM26
hsa-mir-129-5p	RRAGC
hsa-mir-129-5p	PAPD5
hsa-mir-129-5p	GMCL1
hsa-mir-129-5p	NOM1
hsa-mir-129-5p	UNKL
hsa-mir-129-5p	REEP1
hsa-mir-129-5p	COA7
hsa-mir-129-5p	SLC2A11
hsa-mir-129-5p	METRN
hsa-mir-129-5p	ZNF655
hsa-mir-129-5p	TSEN34
hsa-mir-129-5p	C1orf50
hsa-mir-129-5p	SMIM7
hsa-mir-129-5p	TTPAL
hsa-mir-129-5p	DCAF10
hsa-mir-129-5p	GLB1L
hsa-mir-129-5p	LRRC2
hsa-mir-129-5p	NKAIN1
hsa-mir-129-5p	CERS4
hsa-mir-129-5p	ZYG11B
hsa-mir-129-5p	AAGAB
hsa-mir-129-5p	ZNF419
hsa-mir-129-5p	GSTCD
hsa-mir-129-5p	PTCD2
hsa-mir-129-5p	METTL8

hsa-mir-129-5p	TNIP3
hsa-mir-129-5p	KIAA0319L
hsa-mir-129-5p	SLC35E1
hsa-mir-129-5p	TRMT2B
hsa-mir-129-5p	FBXL18
hsa-mir-129-5p	C3orf36
hsa-mir-129-5p	BICC1
hsa-mir-129-5p	ZNF703
hsa-mir-129-5p	ORAI2
hsa-mir-129-5p	KLHL15
hsa-mir-129-5p	EPC1
hsa-mir-129-5p	WDR82
hsa-mir-129-5p	PNPLA3
hsa-mir-129-5p	APOL6
hsa-mir-129-5p	LMAN2L
hsa-mir-129-5p	PLA2G12A
hsa-mir-129-5p	FAM83D
hsa-mir-129-5p	TSPAN14
hsa-mir-129-5p	SPRY4
hsa-mir-129-5p	NRIP2
hsa-mir-129-5p	LONP2
hsa-mir-129-5p	MIXL1
hsa-mir-129-5p	KCTD10
hsa-mir-129-5p	KREMEN1
hsa-mir-129-5p	MAGT1
hsa-mir-129-5p	CHCHD5
hsa-mir-129-5p	YIPF4
hsa-mir-129-5p	SLC25A33
hsa-mir-129-5p	CCDC77
hsa-mir-129-5p	LZIC
hsa-mir-129-5p	NIFK
hsa-mir-129-5p	HOOK3
hsa-mir-129-5p	BRSK1
hsa-mir-129-5p	ZBTB37
hsa-mir-129-5p	NTNG2
hsa-mir-129-5p	PRRC2B
hsa-mir-129-5p	CNDP1
hsa-mir-129-5p	NFKBID
hsa-mir-129-5p	NFATC2IP
hsa-mir-129-5p	SLC35B4
hsa-mir-129-5p	MICALCL
hsa-mir-129-5p	LSM10
hsa-mir-129-5p	TMEM241
hsa-mir-129-5p	HIST1H2AH

hsa-mir-129-5p	PAQR8
hsa-mir-129-5p	STON2
hsa-mir-129-5p	SELENOI
hsa-mir-129-5p	SCIN
hsa-mir-129-5p	CHRFAM7A
hsa-mir-129-5p	KBTBD6
hsa-mir-129-5p	KNSTRN
hsa-mir-129-5p	ANGEL2
hsa-mir-129-5p	BOD1
hsa-mir-129-5p	ZNF502
hsa-mir-129-5p	ANKRD44
hsa-mir-129-5p	ZNF845
hsa-mir-129-5p	DSEL
hsa-mir-129-5p	ISPD
hsa-mir-129-5p	SHISA9
hsa-mir-129-5p	C16orf52
hsa-mir-129-5p	C17orf99
hsa-mir-129-5p	ERVMER34-1
hsa-mir-129-5p	RNF103-CHMP3
hsa-mir-129-5p	RBMXL1
hsa-mir-129-5p	RNF165
hsa-mir-129-5p	ARGFX
hsa-mir-129-5p	C6orf132
hsa-mir-129-5p	TEX22
hsa-mir-129-5p	ZBTB8A
hsa-mir-129-5p	NCF1
hsa-mir-129-5p	CCDC169
hsa-mir-129-5p	SLC35E2B
hsa-mir-129-5p	GTF2H5
hsa-mir-129-5p	TRIM72
hsa-mir-212-3p	EIF2S3
hsa-mir-212-3p	ACSL4
hsa-mir-212-3p	ACHE
hsa-mir-212-3p	ADCY1
hsa-mir-212-3p	AMD1
hsa-mir-212-3p	APBA1
hsa-mir-212-3p	ART4
hsa-mir-212-3p	BRCA1
hsa-mir-212-3p	CCNA2
hsa-mir-212-3p	CCNB1
hsa-mir-212-3p	CDKN1A
hsa-mir-212-3p	PLAGL2
hsa-mir-212-3p	PSMA2
hsa-mir-212-3p	GNB1

hsa-mir-212-3p	HOXC4
hsa-mir-212-3p	HSPA1B
hsa-mir-212-3p	KCNJ2
hsa-mir-212-3p	KPNA1
hsa-mir-212-3p	LDLR
hsa-mir-212-3p	LIFR
hsa-mir-212-3p	SMAD2
hsa-mir-212-3p	MECP2
hsa-mir-212-3p	MYC
hsa-mir-212-3p	PFAS
hsa-mir-212-3p	WT1
hsa-mir-212-3p	ZNF711
hsa-mir-212-3p	FXR1
hsa-mir-212-3p	DPF1
hsa-mir-212-3p	KDM5C
hsa-mir-212-3p	FZD6
hsa-mir-212-3p	PEA15
hsa-mir-212-3p	SAP30
hsa-mir-212-3p	USP8
hsa-mir-212-3p	ABCG2
hsa-mir-212-3p	DAZAP2
hsa-mir-212-3p	HS3ST3B1
hsa-mir-212-3p	STAG1
hsa-mir-212-3p	CD226
hsa-mir-212-3p	NUP50
hsa-mir-212-3p	RAB18
hsa-mir-212-3p	RRS1
hsa-mir-212-3p	OTUD3
hsa-mir-212-3p	CRTC1
hsa-mir-212-3p	TSPAN12
hsa-mir-212-3p	SGK3
hsa-mir-212-3p	MTO1
hsa-mir-212-3p	ASF1A
hsa-mir-212-3p	AGO1
hsa-mir-212-3p	EML4
hsa-mir-212-3p	GMNN
hsa-mir-212-3p	PHF20L1
hsa-mir-212-3p	IER3IP1
hsa-mir-212-3p	IRAK4
hsa-mir-212-3p	PTCH1
hsa-mir-212-3p	PXN
hsa-mir-212-3p	RAB5B
hsa-mir-212-3p	RB1
hsa-mir-212-3p	RBP2

hsa-mir-212-3p	RFXAP
hsa-mir-212-3p	SOD2
hsa-mir-212-3p	SOX4
hsa-mir-212-3p	SOX11
hsa-mir-212-3p	ELOC
hsa-mir-212-3p	TJP1
hsa-mir-212-3p	CHAC1
hsa-mir-212-3p	SH3TC2
hsa-mir-212-3p	DCAF17
hsa-mir-212-3p	SLC25A32
hsa-mir-212-3p	VMP1
hsa-mir-212-3p	CALN1
hsa-mir-212-3p	SLC10A7
hsa-mir-212-3p	HOOK3
hsa-mir-212-3p	TJAP1
hsa-mir-212-3p	ZNF280B
hsa-mir-212-3p	TRUB1
hsa-mir-212-3p	UBXN2A
hsa-mir-212-3p	BMPER
hsa-mir-212-3p	OLFML2A
hsa-mir-212-3p	ARID2
hsa-mir-212-3p	TWISTNB
hsa-mir-212-3p	ZNF724
hsa-mir-212-3p	CCDC169
hsa-mir-212-3p	OCLN
hsa-mir-212-3p	SETD5
hsa-mir-212-3p	PARP11
hsa-mir-212-3p	CYP20A1
hsa-mir-212-3p	TNRC6C
hsa-mir-212-3p	PRDM15
hsa-mir-212-3p	C6orf106
hsa-mir-212-3p	PRAMEF1
hsa-mir-212-3p	GID4
hsa-mir-212-3p	ALKBH4
hsa-mir-212-3p	BRWD1
hsa-mir-212-3p	SLC38A2
hsa-mir-132-3p	GJE1
hsa-mir-132-3p	CCDC169
hsa-mir-132-3p	EOGT
hsa-mir-132-3p	KRT6C
hsa-mir-132-3p	COX8C
hsa-mir-132-3p	KCTD4
hsa-mir-132-3p	FAM221B
hsa-mir-132-3p	ZNF724

hsa-mir-132-3p	SYNDIG1L
hsa-mir-132-3p	KRTAP4-11
hsa-mir-132-3p	NCS1
hsa-mir-132-3p	TSPAN12
hsa-mir-132-3p	MTO1
hsa-mir-132-3p	ASF1A
hsa-mir-132-3p	FGF22
hsa-mir-132-3p	B3GAT1
hsa-mir-132-3p	AGO2
hsa-mir-132-3p	EML4
hsa-mir-132-3p	SEC61A1
hsa-mir-132-3p	DMGDH
hsa-mir-132-3p	MDFIC
hsa-mir-132-3p	GMNN
hsa-mir-132-3p	PHF20L1
hsa-mir-132-3p	IER3IP1
hsa-mir-132-3p	IRAK4
hsa-mir-132-3p	JPT1
hsa-mir-132-3p	ZDHHC2
hsa-mir-132-3p	SNX7
hsa-mir-132-3p	POLK
hsa-mir-132-3p	HACD3
hsa-mir-132-3p	CTDSPL2
hsa-mir-132-3p	BRWD1
hsa-mir-132-3p	SLC38A2
hsa-mir-132-3p	CCSER2
hsa-mir-132-3p	GNL3L
hsa-mir-132-3p	ALKBH4
hsa-mir-132-3p	MTMR10
hsa-mir-132-3p	KLHL11
hsa-mir-132-3p	VPS13D
hsa-mir-132-3p	SETD5
hsa-mir-132-3p	ETNK2
hsa-mir-132-3p	YY1AP1
hsa-mir-132-3p	UBE2W
hsa-mir-132-3p	LGR4
hsa-mir-132-3p	TRIM36
hsa-mir-132-3p	SOX6
hsa-mir-132-3p	DOCK10
hsa-mir-132-3p	INTS8
hsa-mir-132-3p	MUC13
hsa-mir-132-3p	PARP11
hsa-mir-132-3p	RTN4
hsa-mir-132-3p	CYP20A1

hsa-mir-132-3p	TNRC6C
hsa-mir-132-3p	PRX
hsa-mir-132-3p	AASDHPPT
hsa-mir-132-3p	FKBP10
hsa-mir-132-3p	PRDM15
hsa-mir-132-3p	PRSS22
hsa-mir-132-3p	SAMSN1
hsa-mir-132-3p	C6orf106
hsa-mir-132-3p	PRAMEF1
hsa-mir-132-3p	C8orf33
hsa-mir-132-3p	GID4
hsa-mir-132-3p	CHAC1
hsa-mir-132-3p	RNF128
hsa-mir-132-3p	SH3TC2
hsa-mir-132-3p	FAT4
hsa-mir-132-3p	GSTCD
hsa-mir-132-3p	DCAF17
hsa-mir-132-3p	SLC25A32
hsa-mir-132-3p	VMP1
hsa-mir-132-3p	TMEM47
hsa-mir-132-3p	TLN2
hsa-mir-132-3p	CALN1
hsa-mir-132-3p	SLC10A7
hsa-mir-132-3p	HOOK3
hsa-mir-132-3p	FBN3
hsa-mir-132-3p	EBPL
hsa-mir-132-3p	PARP10
hsa-mir-132-3p	WNT3A
hsa-mir-132-3p	EMILIN3
hsa-mir-132-3p	DEPDC7
hsa-mir-132-3p	ADAMTSL1
hsa-mir-132-3p	TJAP1
hsa-mir-132-3p	OSBPL8
hsa-mir-132-3p	SFXN2
hsa-mir-132-3p	FAT3
hsa-mir-132-3p	ARL14EP
hsa-mir-132-3p	CMTM3
hsa-mir-132-3p	CST9L
hsa-mir-132-3p	FAM199X
hsa-mir-132-3p	MUC17
hsa-mir-132-3p	ZNF280B
hsa-mir-132-3p	TRUB1
hsa-mir-132-3p	KBTBD3
hsa-mir-132-3p	FAM81A

hsa-mir-132-3p	ANKRD29
hsa-mir-132-3p	ARL6IP6
hsa-mir-132-3p	SYNE3
hsa-mir-132-3p	SPRED1
hsa-mir-132-3p	UBXN2A
hsa-mir-132-3p	LCA5
hsa-mir-132-3p	BMPER
hsa-mir-132-3p	OLFML2A
hsa-mir-132-3p	ARID2
hsa-mir-132-3p	CC2D1B
hsa-mir-132-3p	ARL13B
hsa-mir-132-3p	ADGRF4
hsa-mir-132-3p	TWISTNB
hsa-mir-132-3p	SYT14
hsa-mir-132-3p	ADH1A
hsa-mir-132-3p	ALK
hsa-mir-132-3p	AMD1
hsa-mir-132-3p	ANXA2
hsa-mir-132-3p	APBA1
hsa-mir-132-3p	TRIM23
hsa-mir-132-3p	ARF6
hsa-mir-132-3p	ARR3
hsa-mir-132-3p	ART4
hsa-mir-132-3p	TNFRSF17
hsa-mir-132-3p	BDNF
hsa-mir-132-3p	BNIP2
hsa-mir-132-3p	CA1
hsa-mir-132-3p	CALU
hsa-mir-132-3p	CASP7
hsa-mir-132-3p	CCNA2
hsa-mir-132-3p	CCNB1
hsa-mir-132-3p	CDH3
hsa-mir-132-3p	CDKN1A
hsa-mir-132-3p	CFTR
hsa-mir-132-3p	CHRNA5
hsa-mir-132-3p	CR2
hsa-mir-132-3p	CRK
hsa-mir-132-3p	CSTF3
hsa-mir-132-3p	CYP2E1
hsa-mir-132-3p	CYP26A1
hsa-mir-132-3p	CD55
hsa-mir-132-3p	HBEGF
hsa-mir-132-3p	ECT2
hsa-mir-132-3p	EGFR

hsa-mir-132-3p	EIF2S3
hsa-mir-132-3p	EPHA4
hsa-mir-132-3p	EYA4
hsa-mir-132-3p	FABP7
hsa-mir-132-3p	ACSL4
hsa-mir-132-3p	FGF2
hsa-mir-132-3p	FOXO1
hsa-mir-132-3p	FUT1
hsa-mir-132-3p	GATA3
hsa-mir-132-3p	NIPSNAP2
hsa-mir-132-3p	GCNT1
hsa-mir-132-3p	GK
hsa-mir-132-3p	GNB1
hsa-mir-132-3p	GTF2H1
hsa-mir-132-3p	HOXC4
hsa-mir-132-3p	HSPA1B
hsa-mir-132-3p	IMPA1
hsa-mir-132-3p	IRAK1
hsa-mir-132-3p	KCNJ9
hsa-mir-132-3p	KIR3DL1
hsa-mir-132-3p	KIR3DL2
hsa-mir-132-3p	KPNA1
hsa-mir-132-3p	RPSA
hsa-mir-132-3p	STMN1
hsa-mir-132-3p	LDLR
hsa-mir-132-3p	LFNG
hsa-mir-132-3p	LIFR
hsa-mir-132-3p	LIG4
hsa-mir-132-3p	LOXL1
hsa-mir-132-3p	SMAD2
hsa-mir-132-3p	MAP1B
hsa-mir-132-3p	MEF2A
hsa-mir-132-3p	MAP3K3
hsa-mir-132-3p	MMP9
hsa-mir-132-3p	MMP13
hsa-mir-132-3p	NAP1L1
hsa-mir-132-3p	NBN
hsa-mir-132-3p	NDUFB10
hsa-mir-132-3p	OAS2
hsa-mir-132-3p	PFAS
hsa-mir-132-3p	PLAGL2
hsa-mir-132-3p	PLSCR1
hsa-mir-132-3p	MAPK1
hsa-mir-132-3p	PSMA2

hsa-mir-132-3p	PSMD12
hsa-mir-132-3p	PTGS2
hsa-mir-132-3p	RAB5B
hsa-mir-132-3p	RAF1
hsa-mir-132-3p	RASA1
hsa-mir-132-3p	RB1
hsa-mir-132-3p	RPL7
hsa-mir-132-3p	RPS5
hsa-mir-132-3p	S100A1
hsa-mir-132-3p	SDF2
hsa-mir-132-3p	SH3BGRL
hsa-mir-132-3p	SLC2A1
hsa-mir-132-3p	SMN1
hsa-mir-132-3p	SOX4
hsa-mir-132-3p	SOX5
hsa-mir-132-3p	SPAST
hsa-mir-132-3p	SSR3
hsa-mir-132-3p	STAU1
hsa-mir-132-3p	ELOC
hsa-mir-132-3p	TFPI
hsa-mir-132-3p	THBS1
hsa-mir-132-3p	TSPAN6
hsa-mir-132-3p	VDAC2
hsa-mir-132-3p	ZNF711
hsa-mir-132-3p	ZNF148
hsa-mir-132-3p	ZNF236
hsa-mir-132-3p	MLLT10
hsa-mir-132-3p	FXR1
hsa-mir-132-3p	TAF15
hsa-mir-132-3p	GDF5
hsa-mir-132-3p	KDM5C
hsa-mir-132-3p	FZD6
hsa-mir-132-3p	PIP5K1A
hsa-mir-132-3p	PIK3R3
hsa-mir-132-3p	CNTNAP1
hsa-mir-132-3p	PLA2G4C
hsa-mir-132-3p	CD164
hsa-mir-132-3p	ATP6V0E1
hsa-mir-132-3p	USP8
hsa-mir-132-3p	PDLIM7
hsa-mir-132-3p	ARHGAP32
hsa-mir-132-3p	DAZAP2
hsa-mir-132-3p	HS3ST3B1
hsa-mir-132-3p	NR1D2

hsa-mir-132-3p	ACTR2
hsa-mir-132-3p	FRY
hsa-mir-132-3p	SPRY1
hsa-mir-132-3p	STAG1
hsa-mir-132-3p	RACK1
hsa-mir-132-3p	EXOC5
hsa-mir-132-3p	YKT6
hsa-mir-132-3p	CD226
hsa-mir-132-3p	CHL1
hsa-mir-132-3p	NUP50
hsa-mir-132-3p	NCKAP1
hsa-mir-132-3p	PLPBP
hsa-mir-132-3p	TUSC2
hsa-mir-132-3p	ZNF652
hsa-mir-132-3p	RAB18
hsa-mir-132-3p	TBC1D9
hsa-mir-132-3p	RRS1
hsa-mir-132-3p	FBXO28
hsa-mir-132-3p	ANKRD28
hsa-mir-132-3p	OTUD3
hsa-mir-132-3p	CAMSAP2
hsa-mir-132-3p	CSTF2T
hsa-mir-132-3p	SMCHD1
hsa-mir-132-3p	CRTC1
hsa-mir-132-3p	SIRT1
hsa-mir-132-3p	OCLN
hsa-mir-132-3p	RTEL1-TNFRSF6B
hsa-mir-376c-3p	DFFA
hsa-mir-376c-3p	TIMM8A
hsa-mir-376c-3p	CENPA
hsa-mir-376c-3p	DAPK1
hsa-mir-376c-3p	GRB2
hsa-mir-376c-3p	IGF1R
hsa-mir-376c-3p	MC2R
hsa-mir-376c-3p	MDM2
hsa-mir-376c-3p	ORC4
hsa-mir-376c-3p	PDPK1
hsa-mir-376c-3p	CDK14
hsa-mir-376c-3p	PRKCH
hsa-mir-376c-3p	RAB1A
hsa-mir-376c-3p	SIAH2
hsa-mir-376c-3p	SKIL
hsa-mir-376c-3p	ELF4
hsa-mir-376c-3p	NR5A2

hsa-mir-376c-3p	TAT
hsa-mir-376c-3p	TGFA
hsa-mir-376c-3p	TGFBR1
hsa-mir-376c-3p	TSPAN6
hsa-mir-376c-3p	UBE2D3
hsa-mir-376c-3p	UGT2B15
hsa-mir-376c-3p	UGT2B17
hsa-mir-376c-3p	VLDLR
hsa-mir-376c-3p	WNT10B
hsa-mir-376c-3p	FZD6
hsa-mir-376c-3p	CBX4
hsa-mir-376c-3p	SNCB
hsa-mir-376c-3p	SP4
hsa-mir-376c-3p	RPS6KA5
hsa-mir-376c-3p	FXR2
hsa-mir-376c-3p	MORF4L2
hsa-mir-376c-3p	BZW1
hsa-mir-376c-3p	SECISBP2L
hsa-mir-376c-3p	JADE3
hsa-mir-376c-3p	G3BP1
hsa-mir-376c-3p	ENOX2
hsa-mir-376c-3p	FRS2
hsa-mir-376c-3p	SNRNP27
hsa-mir-376c-3p	CHEK2
hsa-mir-376c-3p	FOXJ3
hsa-mir-376c-3p	NEDD4L
hsa-mir-376c-3p	SYF2
hsa-mir-376c-3p	LSM14A
hsa-mir-376c-3p	FAM8A1
hsa-mir-376c-3p	BCL11A
hsa-mir-376c-3p	TMEM106B
hsa-mir-376c-3p	FNBP1L
hsa-mir-376c-3p	YTHDF1
hsa-mir-376c-3p	PSPC1
hsa-mir-376c-3p	ERO1B
hsa-mir-376c-3p	RTN4
hsa-mir-376c-3p	ZNFX1
hsa-mir-376c-3p	ENPP5
hsa-mir-376c-3p	PRRG3
hsa-mir-376c-3p	METTL22
hsa-mir-376c-3p	ATAT1
hsa-mir-376c-3p	DNAJB14
hsa-mir-376c-3p	CYTOR
hsa-mir-376c-3p	ACVR1C

hsa-mir-376c-3p	ARHGAP42
hsa-mir-376c-3p	TVP23C
hsa-mir-376c-3p	PRPS1L1
hsa-mir-376c-3p	RBM20
hsa-mir-376c-3p	ZNF829
hsa-mir-376c-3p	ANKRD36
hsa-mir-376c-3p	GOLGA7B
hsa-mir-376c-3p	TNFRSF10B
hsa-mir-376c-3p	CLDN1
hsa-mir-376c-3p	MTRNR2L2
hsa-mir-376c-3p	MTRNR2L6
hsa-mir-376c-3p	MTRNR2L8
hsa-mir-376c-3p	ATM
hsa-mir-376c-3p	BBS4
hsa-mir-376c-3p	BCL2
hsa-mir-376c-3p	BMI1
hsa-mir-376c-3p	RUNX2
hsa-mir-376c-3p	CD59
hsa-mir-376c-3p	MTRNR2L1
hsa-mir-376c-3p	ADH4
hsa-mir-376c-3p	ANK1
hsa-mir-376c-3p	TMEM170B
hsa-mir-376c-3p	ARF6
hsa-mir-383-5p	CAPN6
hsa-mir-383-5p	DIO1
hsa-mir-383-5p	GAS1
hsa-mir-383-5p	IRF1
hsa-mir-383-5p	KCNJ12
hsa-mir-383-5p	LDHA
hsa-mir-383-5p	NPAT
hsa-mir-383-5p	SLC11A2
hsa-mir-383-5p	NRF1
hsa-mir-383-5p	PLCG2
hsa-mir-383-5p	PPP1R10
hsa-mir-383-5p	GATA6
hsa-mir-383-5p	GLUL
hsa-mir-383-5p	MSH6
hsa-mir-383-5p	HNRNPH1
hsa-mir-383-5p	IGF1R
hsa-mir-383-5p	SRSF2
hsa-mir-383-5p	TAF13
hsa-mir-383-5p	TPM3
hsa-mir-383-5p	TRAF5
hsa-mir-383-5p	TSN

hsa-mir-383-5p	VCP
hsa-mir-383-5p	VEGFA
hsa-mir-383-5p	ZFP37
hsa-mir-383-5p	TWF1
hsa-mir-383-5p	RAD21
hsa-mir-383-5p	RHD
hsa-mir-383-5p	RPL41
hsa-mir-383-5p	SFPQ
hsa-mir-383-5p	ADSS
hsa-mir-383-5p	AHR
hsa-mir-383-5p	ALDH1B1
hsa-mir-383-5p	ATP5G3
hsa-mir-383-5p	ATR
hsa-mir-383-5p	CCND1
hsa-mir-383-5p	MED16
hsa-mir-383-5p	SMC2
hsa-mir-383-5p	NCKAP1
hsa-mir-383-5p	ZNF460
hsa-mir-383-5p	PRDX3
hsa-mir-383-5p	CHEK2
hsa-mir-383-5p	SYNRG
hsa-mir-383-5p	CEP162
hsa-mir-383-5p	CLUAP1
hsa-mir-383-5p	GGA2
hsa-mir-383-5p	WBP2
hsa-mir-383-5p	HEBP2
hsa-mir-383-5p	GABARAPL1
hsa-mir-383-5p	APOL2
hsa-mir-383-5p	MOB4
hsa-mir-383-5p	CLIC4
hsa-mir-383-5p	ZBTB20
hsa-mir-383-5p	FBXW8
hsa-mir-383-5p	PCDH11X
hsa-mir-383-5p	TMOD3
hsa-mir-383-5p	ZBTB21
hsa-mir-383-5p	MRNIP
hsa-mir-383-5p	WAC
hsa-mir-383-5p	SUCO
hsa-mir-383-5p	CTDSPL2
hsa-mir-383-5p	TRMT112
hsa-mir-383-5p	NCKIPSD
hsa-mir-383-5p	CLIC6
hsa-mir-383-5p	RBM27
hsa-mir-383-5p	DDIT4

hsa-mir-383-5p	WDR5B
hsa-mir-383-5p	TRPM7
hsa-mir-383-5p	FNBP1L
hsa-mir-383-5p	C5orf22
hsa-mir-383-5p	YOD1
hsa-mir-383-5p	TMEM30A
hsa-mir-383-5p	CENPN
hsa-mir-383-5p	SLC22A23
hsa-mir-383-5p	CRTC3
hsa-mir-383-5p	RSG1
hsa-mir-383-5p	SH3TC2
hsa-mir-383-5p	PCNX2
hsa-mir-383-5p	KLHL15
hsa-mir-383-5p	GDPD5
hsa-mir-383-5p	PCDH11Y
hsa-mir-383-5p	ATG10
hsa-mir-383-5p	GTPBP3
hsa-mir-383-5p	UNC119B
hsa-mir-383-5p	ZIC5
hsa-mir-383-5p	ANKRD44
hsa-mir-383-5p	DSEL
hsa-mir-383-5p	SCAMP4
hsa-mir-383-5p	SGK494
hsa-mir-383-5p	TRIM71
hsa-mir-383-5p	TMEM199
hsa-mir-383-5p	DIRAS1
hsa-mir-383-5p	RIMBP3C
hsa-mir-383-5p	CREBRF
hsa-mir-383-5p	UBN2
hsa-mir-383-5p	ZDHHC24
hsa-mir-383-5p	PLA2G4D
hsa-mir-383-5p	ZNF699
hsa-mir-383-5p	PEAR1
hsa-mir-383-5p	PICSAR
hsa-mir-383-5p	MICA
hsa-mir-383-5p	HSPE1-MOB4
hsa-mir-383-5p	KIAA0100
hsa-mir-383-5p	SLC35E2
hsa-mir-383-5p	EDF1
hsa-mir-383-5p	TNFSF13
hsa-mir-383-5p	ZMYM2
hsa-mir-383-5p	DYRK2
hsa-mir-383-5p	GOSR1
hsa-mir-383-5p	TMEM59

hsa-mir-383-5p	DDX21
hsa-mir-383-5p	GRAP2
hsa-mir-383-5p	IER2
hsa-mir-505-3p	OGFR
hsa-mir-505-3p	ZNF652
hsa-mir-505-3p	SUB1
hsa-mir-505-3p	EBNA1BP2
hsa-mir-505-3p	LSM4
hsa-mir-505-3p	PTCD1
hsa-mir-505-3p	PLEKHG3
hsa-mir-505-3p	GIGYF2
hsa-mir-505-3p	SERBP1
hsa-mir-505-3p	INTU
hsa-mir-505-3p	KCNMB4
hsa-mir-505-3p	DROSHA
hsa-mir-505-3p	TMOD3
hsa-mir-505-3p	FAHD2A
hsa-mir-505-3p	FIS1
hsa-mir-505-3p	RSRC1
hsa-mir-505-3p	ASB1
hsa-mir-505-3p	IL20RB
hsa-mir-505-3p	TREM1
hsa-mir-505-3p	PUS7
hsa-mir-505-3p	GNL3L
hsa-mir-505-3p	KLHDC4
hsa-mir-505-3p	FNBP1L
hsa-mir-505-3p	CWC25
hsa-mir-505-3p	YTHDF1
hsa-mir-505-3p	ATG2B
hsa-mir-505-3p	RBM28
hsa-mir-505-3p	MAP1S
hsa-mir-505-3p	FANCI
hsa-mir-505-3p	MAML3
hsa-mir-505-3p	OTUD5
hsa-mir-505-3p	ZFP64
hsa-mir-505-3p	CBWD1
hsa-mir-505-3p	BAHD1
hsa-mir-505-3p	RUFY3
hsa-mir-505-3p	DIP2C
hsa-mir-505-3p	RRP1B
hsa-mir-505-3p	GRAMD4
hsa-mir-505-3p	CLUH
hsa-mir-505-3p	BICD2
hsa-mir-505-3p	PPP1R13B

hsa-mir-505-3p	ACAP2
hsa-mir-505-3p	BACE1
hsa-mir-505-3p	PISD
hsa-mir-505-3p	PSMD10
hsa-mir-505-3p	PTPRJ
hsa-mir-505-3p	REST
hsa-mir-505-3p	RGS2
hsa-mir-505-3p	RPL9
hsa-mir-505-3p	RPLP0
hsa-mir-505-3p	RPS15
hsa-mir-505-3p	SALL1
hsa-mir-505-3p	SCD
hsa-mir-505-3p	SEC13
hsa-mir-505-3p	SRSF1
hsa-mir-505-3p	SOD2
hsa-mir-505-3p	SOX11
hsa-mir-505-3p	SYT4
hsa-mir-505-3p	TAF9
hsa-mir-505-3p	TCF7L2
hsa-mir-505-3p	TGFA
hsa-mir-505-3p	TGFBR2
hsa-mir-505-3p	THOP1
hsa-mir-505-3p	TLE3
hsa-mir-505-3p	TNFAIP3
hsa-mir-505-3p	TP53BP1
hsa-mir-505-3p	TPBG
hsa-mir-505-3p	TPD52
hsa-mir-505-3p	TUBB2A
hsa-mir-505-3p	TXNRD1
hsa-mir-505-3p	UBE2D3
hsa-mir-505-3p	UQCRFS1
hsa-mir-505-3p	UTRN
hsa-mir-505-3p	VBP1
hsa-mir-505-3p	ZNF208
hsa-mir-505-3p	FZD5
hsa-mir-505-3p	DGS2
hsa-mir-505-3p	HIST1H4D
hsa-mir-505-3p	LTBP4
hsa-mir-505-3p	NCK2
hsa-mir-505-3p	IRS4
hsa-mir-505-3p	SLC25A12
hsa-mir-505-3p	RTL8C
hsa-mir-505-3p	BTRC
hsa-mir-505-3p	BTAF1

hsa-mir-505-3p	PRC1
hsa-mir-505-3p	MTA2
hsa-mir-505-3p	SNAP29
hsa-mir-505-3p	MORF4L2
hsa-mir-505-3p	SOCS5
hsa-mir-505-3p	MDC1
hsa-mir-505-3p	PDE4DIP
hsa-mir-505-3p	KDM4A
hsa-mir-505-3p	BMS1
hsa-mir-505-3p	PJA2
hsa-mir-505-3p	TLK1
hsa-mir-505-3p	TRIM28
hsa-mir-505-3p	ALYREF
hsa-mir-505-3p	TUBB3
hsa-mir-505-3p	NDRG1
hsa-mir-505-3p	ZBTB18
hsa-mir-505-3p	HMGN4
hsa-mir-505-3p	TM9SF1
hsa-mir-505-3p	CCT7
hsa-mir-505-3p	PAICS
hsa-mir-505-3p	STAMBP
hsa-mir-505-3p	SPINT2
hsa-mir-505-3p	DMRT2
hsa-mir-505-3p	CD226
hsa-mir-505-3p	PNMA2
hsa-mir-505-3p	GRK3
hsa-mir-505-3p	AMFR
hsa-mir-505-3p	XIAP
hsa-mir-505-3p	RHOH
hsa-mir-505-3p	BMP2
hsa-mir-505-3p	CANX
hsa-mir-505-3p	CCND2
hsa-mir-505-3p	CD47
hsa-mir-505-3p	CDC5L
hsa-mir-505-3p	CHEK1
hsa-mir-505-3p	COL4A1
hsa-mir-505-3p	CSE1L
hsa-mir-505-3p	CSF1
hsa-mir-505-3p	CSNK1A1
hsa-mir-505-3p	CTSZ
hsa-mir-505-3p	DDX3X
hsa-mir-505-3p	DDX6
hsa-mir-505-3p	DHX9
hsa-mir-505-3p	TSC22D3

hsa-mir-505-3p	EEF1A1
hsa-mir-505-3p	EIF1AX
hsa-mir-505-3p	ELF4
hsa-mir-505-3p	ELK3
hsa-mir-505-3p	EPHA4
hsa-mir-505-3p	FANCD2
hsa-mir-505-3p	XRCC6
hsa-mir-505-3p	GAS1
hsa-mir-505-3p	GGCX
hsa-mir-505-3p	GPLD1
hsa-mir-505-3p	H3F3B
hsa-mir-505-3p	HDLBP
hsa-mir-505-3p	HNRNPF
hsa-mir-505-3p	HOXA9
hsa-mir-505-3p	HSPA1B
hsa-mir-505-3p	ID4
hsa-mir-505-3p	IGF1
hsa-mir-505-3p	EIF6
hsa-mir-505-3p	JARID2
hsa-mir-505-3p	KCNH1
hsa-mir-505-3p	TNPO1
hsa-mir-505-3p	LGALS8
hsa-mir-505-3p	LIMS1
hsa-mir-505-3p	SMAD2
hsa-mir-505-3p	MAZ
hsa-mir-505-3p	DNAJB9
hsa-mir-505-3p	MKI67
hsa-mir-505-3p	MYBPC1
hsa-mir-505-3p	MYBL2
hsa-mir-505-3p	MYH10
hsa-mir-505-3p	NPTX1
hsa-mir-505-3p	ORC2
hsa-mir-505-3p	OSBP
hsa-mir-505-3p	POLG
hsa-mir-505-3p	POLR2D
hsa-mir-505-3p	PPP1CB
hsa-mir-505-3p	PRKCA
hsa-mir-505-3p	MYO19
hsa-mir-505-3p	EDEM3
hsa-mir-505-3p	TET1
hsa-mir-505-3p	VMP1
hsa-mir-505-3p	TMEM185A
hsa-mir-505-3p	PRRC2B
hsa-mir-505-3p	FUT10

hsa-mir-505-3p	MICALCL
hsa-mir-505-3p	TRIM4
hsa-mir-505-3p	KBTBD6
hsa-mir-505-3p	MCFD2
hsa-mir-505-3p	ZNF845
hsa-mir-505-3p	TMEM132B
hsa-mir-505-3p	ZNF511
hsa-mir-505-3p	ANKRD13B
hsa-mir-505-3p	GJD3
hsa-mir-505-3p	TPRG1L
hsa-mir-505-3p	TYW5
hsa-mir-505-3p	NUDT16
hsa-mir-505-3p	RIMS4
hsa-mir-505-3p	TMEM37
hsa-mir-505-3p	SRXN1
hsa-mir-505-3p	FBXL16
hsa-mir-505-3p	SLC5A12
hsa-mir-505-3p	DGKH
hsa-mir-505-3p	PRICKLE2
hsa-mir-505-3p	FUT11
hsa-mir-505-3p	TUBB
hsa-mir-505-3p	CBWD5
hsa-mir-505-3p	HNRNPA3
hsa-mir-505-3p	OPN5
hsa-mir-505-3p	ZBTB9
hsa-mir-505-3p	FBXO33
hsa-mir-505-3p	NEGR1
hsa-mir-505-3p	C5orf64
hsa-mir-505-3p	ANKRD13D
hsa-mir-505-3p	ACER2
hsa-mir-505-3p	ZNF445
hsa-mir-505-3p	FAM89A
hsa-mir-505-3p	GJB7
hsa-mir-505-3p	SOGA3
hsa-mir-505-3p	BEND4
hsa-mir-505-3p	HIST2H4B
hsa-mir-505-3p	NBPF8
hsa-mir-505-3p	CASTOR2
hsa-mir-505-3p	PET117
hsa-mir-505-3p	TNRC6C
hsa-mir-505-3p	FAM160B1
hsa-mir-505-3p	THADA
hsa-mir-505-3p	UBE2O
hsa-mir-505-3p	NUCKS1

hsa-mir-505-3p	YTHDC2
hsa-mir-505-3p	GID4
hsa-mir-505-3p	NABP2
hsa-mir-505-3p	QSER1
hsa-mir-505-3p	C15orf39
hsa-mir-505-3p	PRDM10
hsa-mir-421	ZFP36L2
hsa-mir-421	CASP2
hsa-mir-421	GRK3
hsa-mir-421	AP1G1
hsa-mir-421	SLC25A6
hsa-mir-421	XIAP
hsa-mir-421	RHOB
hsa-mir-421	RND3
hsa-mir-421	RHOH
hsa-mir-421	ATM
hsa-mir-421	ATP6V0C
hsa-mir-421	DUSP3
hsa-mir-421	E2F6
hsa-mir-421	CASP3
hsa-mir-421	CDH1
hsa-mir-421	CEBPB
hsa-mir-421	CEBPD
hsa-mir-421	COL4A1
hsa-mir-421	COPA
hsa-mir-421	CSE1L
hsa-mir-421	CSNK1A1
hsa-mir-421	DDX3X
hsa-mir-421	DFFB
hsa-mir-421	TSC22D3
hsa-mir-421	HTT
hsa-mir-421	HOXA9
hsa-mir-421	HSP90AA1
hsa-mir-421	ID4
hsa-mir-421	IGF1
hsa-mir-421	INPP4A
hsa-mir-421	ITPK1
hsa-mir-421	JARID2
hsa-mir-421	KCNN3
hsa-mir-421	LBR
hsa-mir-421	LIMS1
hsa-mir-421	SMAD4
hsa-mir-421	MAZ
hsa-mir-421	CD46

hsa-mir-421	FOXO4	
hsa-mir-421	MTAP	
hsa-mir-421	MYBPC1	
hsa-mir-421		2-Sep
hsa-mir-421	NFE2L1	
hsa-mir-421	NPM1	
hsa-mir-421	NPTX1	
hsa-mir-421	PDPK1	
hsa-mir-421	PHKA1	
hsa-mir-421	POLG	
hsa-mir-421	POLR2D	
hsa-mir-421	PPP2CB	
hsa-mir-421	PRCC	
hsa-mir-421	MAPK8	
hsa-mir-421	PSME1	
hsa-mir-421	EEF1A1	
hsa-mir-421	EIF4A1	
hsa-mir-421	EIF4G2	
hsa-mir-421	FAT2	
hsa-mir-421	GALK2	
hsa-mir-421	GAS1	
hsa-mir-421	NR6A1	
hsa-mir-421	GCSH	
hsa-mir-421	GRK6	
hsa-mir-421	NR3C1	
hsa-mir-421	HADHA	
hsa-mir-421	VMP1	
hsa-mir-421	FAM172A	
hsa-mir-421	PARP10	
hsa-mir-421	RAB2B	
hsa-mir-421	TRIM4	
hsa-mir-421	ZNF30	
hsa-mir-421	MCFD2	
hsa-mir-421	RASL10B	
hsa-mir-421	NXPE3	
hsa-mir-421	ASB16	
hsa-mir-421	SFXN1	
hsa-mir-421	PAXBP1	
hsa-mir-421	FAM122A	
hsa-mir-421	GJD3	
hsa-mir-421	TPRG1L	
hsa-mir-421	FAM168B	
hsa-mir-421	RIMS4	
hsa-mir-421	TMEM37	

hsa-mir-421	FBXL16
hsa-mir-421	GAREM2
hsa-mir-421	CREBRF
hsa-mir-421	PPTC7
hsa-mir-421	DGKH
hsa-mir-421	PRICKLE2
hsa-mir-421	OTUD1
hsa-mir-421	CBWD5
hsa-mir-421	NEGR1
hsa-mir-421	SREK1IP1
hsa-mir-421	RGMB
hsa-mir-421	ZNF678
hsa-mir-421	CCDC39
hsa-mir-421	FAM89A
hsa-mir-421	GJB7
hsa-mir-421	C5orf46
hsa-mir-421	RBMXL1
hsa-mir-421	HIST2H4B
hsa-mir-421	NBPF8
hsa-mir-421	CASTOR2
hsa-mir-421	DDX20
hsa-mir-421	ATXN2L
hsa-mir-421	ZNF652
hsa-mir-421	MLXIP
hsa-mir-421	BAHD1
hsa-mir-421	CNOT1
hsa-mir-421	PHLPP2
hsa-mir-421	NCDN
hsa-mir-421	RHOBTB2
hsa-mir-421	ZC3H7B
hsa-mir-421	DNAJC16
hsa-mir-421	SMCHD1
hsa-mir-421	PPP1R13B
hsa-mir-421	SPECC1L
hsa-mir-421	LARS2
hsa-mir-421	SIRT3
hsa-mir-421	CBX7
hsa-mir-421	ACOT9
hsa-mir-421	ZMYND8
hsa-mir-421	BACE1
hsa-mir-421	FKBP8
hsa-mir-421	LSM4
hsa-mir-421	NIPBL
hsa-mir-421	RAB26

hsa-mir-421	NSL1
hsa-mir-421	FAM32A
hsa-mir-421	AGO1
hsa-mir-421	FAM155B
hsa-mir-421	AFF4
hsa-mir-421	INTU
hsa-mir-421	EIF3K
hsa-mir-421	KCNMB4
hsa-mir-421	RACGAP1
hsa-mir-421	LMCD1
hsa-mir-421	GMNN
hsa-mir-421	RPL26L1
hsa-mir-421	ZNF219
hsa-mir-421	DNAJC27
hsa-mir-421	RSRC1
hsa-mir-421	AMZ2
hsa-mir-421	RNF138
hsa-mir-421	TREM1
hsa-mir-421	VSIG10
hsa-mir-421	PQLC2
hsa-mir-421	YTHDF1
hsa-mir-421	GID8
hsa-mir-421	VPS37C
hsa-mir-421	ATG2B
hsa-mir-421	PNPO
hsa-mir-421	RIF1
hsa-mir-421	MIS18BP1
hsa-mir-421	LIN7C
hsa-mir-421	COPRS
hsa-mir-421	ZNF280C
hsa-mir-421	NDC1
hsa-mir-421	DOK4
hsa-mir-421	TSR1
hsa-mir-421	ZFP64
hsa-mir-421	CBWD1
hsa-mir-421	CMAS
hsa-mir-421	DUS3L
hsa-mir-421	CDC42SE2
hsa-mir-421	PARP11
hsa-mir-421	RALGAPB
hsa-mir-421	SENP7
hsa-mir-421	ISY1
hsa-mir-421	TNRC6C
hsa-mir-421	FAM160B1

hsa-mir-421	CREBZF
hsa-mir-421	ARHGAP22
hsa-mir-421	NCAPG
hsa-mir-421	PCNX4
hsa-mir-421	RANBP2
hsa-mir-421	RBBP7
hsa-mir-421	REST
hsa-mir-421	TRIM27
hsa-mir-421	RGR
hsa-mir-421	RGS2
hsa-mir-421	RPL18A
hsa-mir-421	RPS10
hsa-mir-421	RPS18
hsa-mir-421	RREB1
hsa-mir-421	SCD
hsa-mir-421	SRSF7
hsa-mir-421	SLC6A13
hsa-mir-421	SLIT1
hsa-mir-421	SOD2
hsa-mir-421	SRP9
hsa-mir-421	SRPRA
hsa-mir-421	SYT4
hsa-mir-421	TAF9
hsa-mir-421	TCF7L2
hsa-mir-421	TUBB2A
hsa-mir-421	TXNRD1
hsa-mir-421	UBE2D3
hsa-mir-421	UQCRFS1
hsa-mir-421	ZNF208
hsa-mir-421	ZXDA
hsa-mir-421	RASAL1
hsa-mir-421	AP3B1
hsa-mir-421	USO1
hsa-mir-421	BTRC
hsa-mir-421	SLC28A1
hsa-mir-421	VAPA
hsa-mir-421	GOSR1
hsa-mir-421	TTC37
hsa-mir-421	CEP57
hsa-mir-421	KIAA0100
hsa-mir-421	TLK1
hsa-mir-421	DDX39A
hsa-mir-421	TUBA1B
hsa-mir-421	SLC9A6

hsa-mir-421	CAP1
hsa-mir-421	TM9SF1
hsa-mir-421	P3H4
hsa-mir-421	SPINT2
hsa-mir-421	CD226
hsa-mir-421	MTHFD2
hsa-mir-421	PPARGC1A
hsa-mir-421	MSL3
hsa-mir-421	CDC37
hsa-mir-421	TET1
hsa-mir-421	CD276
hsa-mir-421	PRR7
hsa-mir-421	GFOD2
hsa-mir-421	AHNAK
hsa-mir-421	TMEM109
hsa-mir-421	MRPL38
hsa-mir-421	GID4
hsa-mir-421	FBXO11
hsa-mir-421	RAB11FIP1
hsa-mir-421	QSER1
hsa-mir-421	CTC1
hsa-mir-876-5p	EMP2
hsa-mir-876-5p	EPB41L1
hsa-mir-876-5p	P2RX7
hsa-mir-876-5p	P2RY1
hsa-mir-876-5p	PER1
hsa-mir-876-5p	PTPN14
hsa-mir-876-5p	RORA
hsa-mir-876-5p	FOLR1
hsa-mir-876-5p	FOXA2
hsa-mir-876-5p	HSPD1
hsa-mir-876-5p	HTR7
hsa-mir-876-5p	IMPG1
hsa-mir-876-5p	MAOB
hsa-mir-876-5p	MAT2A
hsa-mir-876-5p	DNAJB9
hsa-mir-876-5p	HIST1H3E
hsa-mir-876-5p	RGS5
hsa-mir-876-5p	AKR7A2
hsa-mir-876-5p	TIMELESS
hsa-mir-876-5p	ABCG2
hsa-mir-876-5p	RPS16
hsa-mir-876-5p	ATXN1
hsa-mir-876-5p	SSR2

hsa-mir-876-5p	AURKA
hsa-mir-876-5p	TAPBP
hsa-mir-876-5p	TPM4
hsa-mir-876-5p	ZNF131
hsa-mir-876-5p	SLC10A3
hsa-mir-876-5p	ATP5F1
hsa-mir-876-5p	CALM2
hsa-mir-876-5p	CAPZA1
hsa-mir-876-5p	CLCN6
hsa-mir-876-5p	PYGO1
hsa-mir-876-5p	VPS4A
hsa-mir-876-5p	TOR1B
hsa-mir-876-5p	TMX2
hsa-mir-876-5p	C1RL
hsa-mir-876-5p	PGPEP1
hsa-mir-876-5p	RPP25
hsa-mir-876-5p	RFWD3
hsa-mir-876-5p	TXLNG
hsa-mir-876-5p	ALG1
hsa-mir-876-5p	C8orf4
hsa-mir-876-5p	TAOK1
hsa-mir-876-5p	ANO8
hsa-mir-876-5p	PLEKHG2
hsa-mir-876-5p	METTL16
hsa-mir-876-5p	CTC1
hsa-mir-876-5p	RBM4B
hsa-mir-876-5p	USP48
hsa-mir-876-5p	ZNF502
hsa-mir-876-5p	AGAP1
hsa-mir-876-5p	BRI3BP
hsa-mir-876-5p	PPM1K
hsa-mir-876-5p	FUT11
hsa-mir-876-5p	ZNF707
hsa-mir-876-5p	S100A7A
hsa-mir-876-5p	RBMXL1
hsa-mir-876-5p	ARGFX
hsa-mir-876-5p	C8orf58
hsa-mir-876-5p	TOMM20
hsa-mir-876-5p	WASF2
hsa-mir-876-5p	ZNF256
hsa-mir-876-5p	NDRG1
hsa-mir-876-5p	PRDX3
hsa-mir-876-5p	CBX1
hsa-mir-876-5p	ZNF277

hsa-mir-876-5p	IKZF2
hsa-mir-876-5p	ZNF507
hsa-mir-876-5p	TTLL12
hsa-mir-876-5p	MESD
hsa-mir-876-5p	KIAA0895
hsa-mir-3167	MAOB
hsa-mir-3167	MAT2A
hsa-mir-3167	DNAJB9
hsa-mir-3167	P2RX7
hsa-mir-3167	P2RY1
hsa-mir-3167	PER1
hsa-mir-3167	PTPN14
hsa-mir-3167	RORA
hsa-mir-3167	RPS16
hsa-mir-3167	ATXN1
hsa-mir-3167	SSR2
hsa-mir-3167	AURKA
hsa-mir-3167	TAPBP
hsa-mir-3167	TPM4
hsa-mir-3167	ZNF131
hsa-mir-3167	SLC10A3
hsa-mir-3167	HIST1H3E
hsa-mir-3167	RGS5
hsa-mir-3167	AKR7A2
hsa-mir-3167	TIMELESS
hsa-mir-3167	ABCG2
hsa-mir-3167	TOMM20
hsa-mir-3167	WASF2
hsa-mir-3167	ZNF256
hsa-mir-3167	NDRG1
hsa-mir-3167	PRDX3
hsa-mir-3167	CBX1
hsa-mir-3167	ZNF277
hsa-mir-3167	IKZF2
hsa-mir-3167	ZNF507
hsa-mir-3167	TTLL12
hsa-mir-3167	MESD
hsa-mir-3167	KIAA0895
hsa-mir-3167	PYGO1
hsa-mir-3167	VPS4A
hsa-mir-3167	TOR1B
hsa-mir-3167	TMX2
hsa-mir-3167	C1RL
hsa-mir-3167	PGPEP1

hsa-mir-3167	RPP25
hsa-mir-3167	RFWD3
hsa-mir-3167	TXLNG
hsa-mir-3167	ALG1
hsa-mir-3167	C8orf4
hsa-mir-3167	TAOK1
hsa-mir-3167	ANO8
hsa-mir-3167	PLEKHG2
hsa-mir-3167	METTL16
hsa-mir-3167	CTC1
hsa-mir-3167	RBM4B
hsa-mir-3167	USP48
hsa-mir-3167	SRRM4
hsa-mir-3167	ZNF502
hsa-mir-3167	AGAP1
hsa-mir-3167	BRI3BP
hsa-mir-3167	PPM1K
hsa-mir-3167	FUT11
hsa-mir-3167	ZNF707
hsa-mir-3167	S100A7A
hsa-mir-3167	RBMXL1
hsa-mir-3167	ARGFX
hsa-mir-3167	C8orf58
hsa-mir-3167	CLCN6
hsa-mir-3167	ATP5F1
hsa-mir-3167	CALM2
hsa-mir-3167	CAPZA1
hsa-mir-3167	FOXA2
hsa-mir-3167	EMP2
hsa-mir-3167	EPB41L1
hsa-mir-3167	FOLR1
hsa-mir-3167	HSPD1
hsa-mir-3167	HTR7
hsa-mir-3167	IMPG1
hsa-mir-4428	UBE2G1
hsa-mir-4428	EIF4H
hsa-mir-4428	SF1
hsa-mir-4428	ZNF8
hsa-mir-4428	ZNF84
hsa-mir-4428	LUZP1
hsa-mir-4428	TAGLN2
hsa-mir-4428	TP63
hsa-mir-4428	KCNK5
hsa-mir-4428	VAMP8

hsa-mir-4428	CD164	
hsa-mir-4428	NOLC1	
hsa-mir-4428	GTF3C4	
hsa-mir-4428	TGFBRAP1	
hsa-mir-4428	SH3PXD2A	
hsa-mir-4428	VGLL4	
hsa-mir-4428	TRAM2	
hsa-mir-4428	SPRY1	
hsa-mir-4428	SPON2	
hsa-mir-4428	VAV3	
hsa-mir-4428	GIPC1	
hsa-mir-4428	ARPP19	
hsa-mir-4428	ZNF273	
hsa-mir-4428	RALBP1	
hsa-mir-4428	MAPRE3	
hsa-mir-4428	MRPS27	
hsa-mir-4428	FAM168A	
hsa-mir-4428	SLC7A8	
hsa-mir-4428	PSD4	
hsa-mir-4428	CD2AP	
hsa-mir-4428	CADM1	
hsa-mir-4428	ANAPC13	
hsa-mir-4428	ZNF385A	
hsa-mir-4428	CNTNAP2	
hsa-mir-4428	SLITRK5	
hsa-mir-4428	GPKOW	
hsa-mir-4428	A1CF	
hsa-mir-4428	RAX	
hsa-mir-4428	TMED5	
hsa-mir-4428	SLC35C2	
hsa-mir-4428	WAC	
hsa-mir-4428	CSNK1G1	
hsa-mir-4428	KLHL24	
hsa-mir-4428	SAMD4B	
hsa-mir-4428	STRADB	
hsa-mir-4428	RBM38	
hsa-mir-4428	IWS1	
hsa-mir-4428	RUFY2	
hsa-mir-4428	PSENEN	
hsa-mir-4428	EIF5A2	
hsa-mir-4428	NUFIP2	
hsa-mir-4428	PLA2G2F	
hsa-mir-4428		1-Mar
hsa-mir-4428	CREB3L2	

hsa-mir-4428	METTL22
hsa-mir-4428	C1orf115
hsa-mir-4428	MYH14
hsa-mir-4428	NOL10
hsa-mir-4428	TTYH3
hsa-mir-4428	ARHGAP39
hsa-mir-4428	RAB1B
hsa-mir-4428	MIDN
hsa-mir-4428	RASL10B
hsa-mir-4428	MRRF
hsa-mir-4428	RP1L1
hsa-mir-4428	PRRT2
hsa-mir-4428	MB21D1
hsa-mir-4428	SNAP47
hsa-mir-4428	SLC16A10
hsa-mir-4428	C2CD4C
hsa-mir-4428	TCHHL1
hsa-mir-4428	ARHGAP42
hsa-mir-4428	DUSP18
hsa-mir-4428	PPP1R1C
hsa-mir-4428	AMOTL1
hsa-mir-4428	ZFP1
hsa-mir-4428	TTLL9
hsa-mir-4428	TOGARAM2
hsa-mir-4428	TXLNA
hsa-mir-4428	LGI3
hsa-mir-4428	SIAH3
hsa-mir-4428	CAVIN1
hsa-mir-4428	ZADH2
hsa-mir-4428	MSL1
hsa-mir-4428	PABPC1L2A
hsa-mir-4428	LCE1B
hsa-mir-4428	PLEKHM3
hsa-mir-4428	BEND4
hsa-mir-4428	PABPC1L2B
hsa-mir-4428	LINC01556
hsa-mir-4428	ANXA11
hsa-mir-4428	CALM3
hsa-mir-4428	CALR
hsa-mir-4428	CASP2
hsa-mir-4428	CCND2
hsa-mir-4428	CHRM3
hsa-mir-4428	DDX6
hsa-mir-4428	DFFB

hsa-mir-4428	COCH
hsa-mir-4428	DLX6
hsa-mir-4428	EFNB1
hsa-mir-4428	ELF3
hsa-mir-4428	EN2
hsa-mir-4428	ENSA
hsa-mir-4428	EP300
hsa-mir-4428	FPR1
hsa-mir-4428	GRSF1
hsa-mir-4428	FOXA1
hsa-mir-4428	IGF2
hsa-mir-4428	INCENP
hsa-mir-4428	ITPKB
hsa-mir-4428	KPNA4
hsa-mir-4428	TNPO1
hsa-mir-4428	LDLR
hsa-mir-4428	MAX
hsa-mir-4428	MECP2
hsa-mir-4428	MID1
hsa-mir-4428	MOG
hsa-mir-4428	TRIM37
hsa-mir-4428	NAB2
hsa-mir-4428	NDUFS1
hsa-mir-4428	NPM1
hsa-mir-4428	CDK14
hsa-mir-4428	PTK7
hsa-mir-4428	RAD21
hsa-mir-4428	RFX3
hsa-mir-4428	RPS15A
hsa-mir-4428	SHMT2
hsa-mir-4428	SIX3
hsa-mir-4428	SNRPB
hsa-mir-4428	SOX4
hsa-mir-4428	SP1
hsa-mir-4428	STK4
hsa-mir-4428	TMBIM6
hsa-mir-4428	TFAP4
hsa-mir-4428	TFDP2
hsa-mir-4428	TPM3
hsa-mir-4761-3p	RHOG
hsa-mir-4761-3p	DNM2
hsa-mir-4761-3p	MARK2
hsa-mir-4761-3p	EVX1
hsa-mir-4761-3p	HNRNPU

hsa-mir-4761-3p	ID2
hsa-mir-4761-3p	MAZ
hsa-mir-4761-3p	MDK
hsa-mir-4761-3p	PPP1R12A
hsa-mir-4761-3p	MAPK1
hsa-mir-4761-3p	SSTR3
hsa-mir-4761-3p	ZNF223
hsa-mir-4761-3p	BTG2
hsa-mir-4761-3p	TAGLN2
hsa-mir-4761-3p	OGT
hsa-mir-4761-3p	TM9SF1
hsa-mir-4761-3p	ARPP19
hsa-mir-4761-3p	ZNF460
hsa-mir-4761-3p	MTUS2
hsa-mir-4761-3p	JADE2
hsa-mir-4761-3p	ZNF281
hsa-mir-4761-3p	ATP6V0A2
hsa-mir-4761-3p	SMUG1
hsa-mir-4761-3p	TMEM2
hsa-mir-4761-3p	BRD7
hsa-mir-4761-3p	SH3GLB1
hsa-mir-4761-3p	CTDSPL2
hsa-mir-4761-3p	WDR45B
hsa-mir-4761-3p	CCNL1
hsa-mir-4761-3p	ENTPD7
hsa-mir-4761-3p	RAB25
hsa-mir-4761-3p	NABP1
hsa-mir-4761-3p	SH3TC2
hsa-mir-4761-3p	ZMAT4
hsa-mir-4761-3p	RUBCNL
hsa-mir-4761-3p	ZNF594
hsa-mir-4761-3p	MIDN
hsa-mir-4761-3p	MCFD2
hsa-mir-4761-3p	IFFO2
hsa-mir-4761-3p	TRIM71
hsa-mir-4761-3p	SESN3
hsa-mir-4761-3p	ZNF322P1
hsa-mir-4761-3p	POTEG
hsa-mir-4761-3p	POTEM
hsa-mir-873-3p	ALDH1B1
hsa-mir-873-3p	SERPING1
hsa-mir-873-3p	CA6
hsa-mir-873-3p	CD40LG
hsa-mir-873-3p	CDKN1A

hsa-mir-873-3p	CHD3
hsa-mir-873-3p	COL9A2
hsa-mir-873-3p	CRK
hsa-mir-873-3p	CSTF1
hsa-mir-873-3p	EGR3
hsa-mir-873-3p	ENSA
hsa-mir-873-3p	ERCC4
hsa-mir-873-3p	F2RL2
hsa-mir-873-3p	FKBP1A
hsa-mir-873-3p	GABRB1
hsa-mir-873-3p	HDGF
hsa-mir-873-3p	CXCL10
hsa-mir-873-3p	IRAK2
hsa-mir-873-3p	KCNN3
hsa-mir-873-3p	LYZ
hsa-mir-873-3p	NUCB1
hsa-mir-873-3p	OLR1
hsa-mir-873-3p	CDK16
hsa-mir-873-3p	NPY4R
hsa-mir-873-3p	MAP2K2
hsa-mir-873-3p	PRKX
hsa-mir-873-3p	TMPRSS15
hsa-mir-873-3p	QSOX1
hsa-mir-873-3p	RPL12
hsa-mir-873-3p	RPL18A
hsa-mir-873-3p	MSMO1
hsa-mir-873-3p	TRAPPC2
hsa-mir-873-3p	TADA2A
hsa-mir-873-3p	KLF10
hsa-mir-873-3p	TUFT1
hsa-mir-873-3p	UGDH
hsa-mir-873-3p	UQCRFS1
hsa-mir-873-3p	VHL
hsa-mir-873-3p	VSNL1
hsa-mir-873-3p	ZFX
hsa-mir-873-3p	ZNF157
hsa-mir-873-3p	FZD5
hsa-mir-873-3p	HIST1H2BG
hsa-mir-873-3p	AKR7A2
hsa-mir-873-3p	EIF3H
hsa-mir-873-3p	SLC16A3
hsa-mir-873-3p	RASSF9
hsa-mir-873-3p	B4GALT5
hsa-mir-873-3p	SOCS5

hsa-mir-873-3p	PPIP5K1
hsa-mir-873-3p	HEPH
hsa-mir-873-3p	TRIM66
hsa-mir-873-3p	SLC35E2
hsa-mir-873-3p	ABCC5
hsa-mir-873-3p	DCAF7
hsa-mir-873-3p	PCGF3
hsa-mir-873-3p	COLEC10
hsa-mir-873-3p	NUPL2
hsa-mir-873-3p	DDX19B
hsa-mir-873-3p	SEPHS1
hsa-mir-873-3p	KIN
hsa-mir-873-3p	POFUT2
hsa-mir-873-3p	CSTF2T
hsa-mir-873-3p	USP22
hsa-mir-873-3p	JADE2
hsa-mir-873-3p	ATP6V0A2
hsa-mir-873-3p	TMEM245
hsa-mir-873-3p	GAPVD1
hsa-mir-873-3p	FBXL3
hsa-mir-873-3p	ARL5A
hsa-mir-873-3p	SIGLEC8
hsa-mir-873-3p	HCAR1
hsa-mir-873-3p	SESN1
hsa-mir-873-3p	TMED7
hsa-mir-873-3p	DCTN4
hsa-mir-873-3p	ANGPT4
hsa-mir-873-3p	PHF7
hsa-mir-873-3p	MBD3
hsa-mir-873-3p	GPR173
hsa-mir-873-3p	CCSER2
hsa-mir-873-3p	GNL3L
hsa-mir-873-3p	BNC2
hsa-mir-873-3p	ZNF331
hsa-mir-873-3p	CAMK2N1
hsa-mir-873-3p	POLR3E
hsa-mir-873-3p	ERBIN
hsa-mir-873-3p	PCNP
hsa-mir-873-3p	PHTF2
hsa-mir-873-3p	CC2D2A
hsa-mir-873-3p	USP36
hsa-mir-873-3p	KIAA1456
hsa-mir-873-3p	RANBP10
hsa-mir-873-3p	SH2D4A

hsa-mir-873-3p	HERPUD2
hsa-mir-873-3p	NBEAL1
hsa-mir-873-3p	METRN
hsa-mir-873-3p	CDC73
hsa-mir-873-3p	TMC5
hsa-mir-873-3p	TRMT2B
hsa-mir-873-3p	CLPB
hsa-mir-873-3p	ZNF611
hsa-mir-873-3p	C9orf64
hsa-mir-873-3p	ZBED3
hsa-mir-873-3p	FAXC
hsa-mir-873-3p	HES7
hsa-mir-873-3p	HIST1H2AH
hsa-mir-873-3p	MIDN
hsa-mir-873-3p	KNSTRN
hsa-mir-873-3p	TIMM29
hsa-mir-873-3p	MYLK3
hsa-mir-873-3p	DSEL
hsa-mir-873-3p	SYAP1
hsa-mir-873-3p	FAM83F
hsa-mir-873-3p	TSPEAR-AS2
hsa-mir-873-3p	TTC39C
hsa-mir-873-3p	ZNF440
hsa-mir-873-3p	CHCHD4
hsa-mir-873-3p	SLC2A14
hsa-mir-873-3p	ZNF417
hsa-mir-873-3p	SPC24
hsa-mir-873-3p	CKAP2L
hsa-mir-873-3p	CNTN4
hsa-mir-873-3p	PPTC7
hsa-mir-873-3p	OR7D2
hsa-mir-873-3p	AAED1
hsa-mir-873-3p	ARID2
hsa-mir-873-3p	STK32A
hsa-mir-873-3p	SAMD9L
hsa-mir-873-3p	RNF152
hsa-mir-873-3p	TMED4
hsa-mir-873-3p	SCUBE3
hsa-mir-873-3p	NKAPL
hsa-mir-873-3p	DEFB105A
hsa-mir-873-3p	ZNF549
hsa-mir-873-3p	SPRYD4
hsa-mir-873-3p	C17orf105
hsa-mir-873-3p	FAM102B

hsa-mir-873-3p	ZNF619
hsa-mir-873-3p	COX18
hsa-mir-873-3p	RPL7L1
hsa-mir-873-3p	C9orf47
hsa-mir-873-3p	ANKRD42
hsa-mir-873-3p	KLHL38
hsa-mir-873-3p	SMTNL2
hsa-mir-873-3p	ZNF850
hsa-mir-873-3p	MTX3
hsa-mir-873-3p	PLA2G2C
hsa-mir-873-3p	FOXL2NB
hsa-mir-873-3p	MXRA7
hsa-mir-873-3p	ZNF716
hsa-mir-873-3p	FBXO47
hsa-mir-873-3p	DEFB105B
hsa-mir-873-3p	ZNF487
hsa-mir-873-3p	STMP1
hsa-mir-873-3p	LINC01556
hsa-mir-873-3p	HTR5A-AS1
hsa-mir-873-3p	KLLN
hsa-mir-873-3p	ZNF783
hsa-mir-873-3p	SLFN12L

Table S3. Expression levels of potential miRNAs of PTTG3P in breast cancer and normal controls.

miRNA ID	Accession	Average expression in cancer	Average expression in normal	P-value
hsa-miR-505-3p	MIMAT0002876	42.77	39.06	0.022
hsa-miR-421	MIMAT0003339	2.23	1.15	5.70E-07
hsa-miR-4428	MIMAT0018943	0.01	0.01	0.77
hsa-miR-873-3p	MIMAT0022717	0.04	0.01	0.05
hsa-miR-3167	MIMAT0015042	0.01	0.01	0.8
hsa-miR-876-5p	MIMAT0004924	0.04	0.03	0.72
hsa-miR-4761-3p	MIMAT0019909	0.01	0.01	0.52
hsa-miR-129-5p	MIMAT0000242	4.71	6.6	2.70E-15
hsa-miR-212-3p	MIMAT0000269	3.97	4.03	0.89
hsa-miR-132-3p	MIMAT0000426	74.61	98.08	0.093
hsa-miR-376c-3p	MIMAT0000720	4.42	9.37	1.80E-14
hsa-miR-383-5p	MIMAT0000738	1.67	4.3	2.00E-41

Table S4. Co-expressed genes of PTTG3P obtained by UALCAN database.

Co-expressed genes of PTTG3P
PTTG1
UBE2C
CDCA3
TROAP
AURKB
CENPA
CKS2
SKA1
CDC25C
CCNB2
CDC20
CENPW
KIF18B
PIF1
KIF2C
HMMR
CDC45
UBE2T
HJURP
KIFC1
CCNB1
ZNF695
RAD54L
TPX2
TACC3
E2F8
E2F1
KIF20A
NCAPG
OIP5
CDCA8
TUBA1C
NDC80
SNRPG
PLK1
UBE2S
CKS1B
SPC25
LMNB2
PA2G4P4
CCDC99
SNRPA

MRPS5
KIF15
FAM64A
C1orf135
SNRPD1
C16orf61
CDT1
UQCRH
CDKN3
DSCC1
BIRC5
NUF2
FAM54A
C17orf53
MELK
MAGOH
FAM72B
FOXM1
PSRC1
TOMM5
CENPN
DTD1
GTSE1
TCOF1
LYAR
BOLA3
DEPDC1B
H2AFZ
TRIP13
DDX12
DLGAP5
LMNB1
EZH2
ORC6L
MND1
RANBP1
RNASEH2A
CDC25A
KIF4A
RFC4
SKA3
TTK
NUSAP1
CHEK2

KIF11
CDKN2A
CEP55
NOP2
EXO1
NEK2
WDR4
RECQL4
ORC1L
LSM2
FAM83D
RAD51AP1
TUBA1B
TRAIP
PHF19
FAM136A
MYBL2
SGOL1
MIR17HG
DBF4
MCM7
UQCRHL
CCNA2
STMN1
NOP16
NDUFA9
CDCA5
DTYMK
PSMB2
RAD51
SNRPC
MRPL51
PKMYT1
C15orf23
BUB1
MXD3
EPR1
POLQ
SHFM1
PFDN2
NDUFAF4
TPI1
CENPH
PBK

PDCD5
LOC388955
DEPDC1
FEN1
C6orf129
CCDC58
CDCA7
HMGB2
SLC25A19
KIF23
MFSD2B
ERCC6L
PSMG1
ATP5EP2
NCAPH
CDK1
MAD2L1
NPM3
ECE2
PDSS1
C9orf40
ASPM
NEIL3
GSG2
LOC100130932
GAPDH
MED30
NUDT1
ESPL1
FBXO5
PPIL5
FAM72A
DONSON
GMNN
HMGA1
KIF14
MAGOHB
TCF19
ATP5J2
MLF2
PRELID1
FANCA
POLR2F
LOC341056

RFC2
HNRNPAB
AURKAPS1
SF3B14
GRK6
RPIA
RCCD1
KIF18A
CENPM
PDHA1
GINS1
RHEB
AURKA
THOC4
RRP1
NCAPG2
BUB1B
BRIX1
TOMM40
LBR
CTPS
ENY2
WDR67
NUDT5
CHAC2
CEBPB
SNHG1
ASF1B
NXT1
LSM7
TXN
C12orf48
C5orf34
DDX11
NASP
DDX39
POC1A
C9orf100
FANCG
BLM
GPSM2
FAM72D
DAZAP1
ANP32E

PSMG3
FANCE

Table S5. Sequences of primers used in this study.

qRT-PCR primers		Sequences
PTTG3P	Forward primer	GGGGTCTGGACCTTCAATCAA
PTTG3P	Reverse primer	GCTTTAGGTAAGGATGTGGGA
PTTG1	Forward primer	ACCCGTGTGGTTGCTAAGG
PTTG1	Reverse primer	ACGTGGTGTTGAAACTTGAGAT
PTTG2	Forward primer	ATTGGAGAACCAGGCACC
PTTG2	Reverse primer	CGTCGTGTTAAAACTTGAGATA
U6	Forward primer	CTCGCTTCGGCAGCACA
U6	Reverse primer	GAAGCCUAGAGGAUUGAAGGG
GAPDH	Forward primer	AATGGACAACTGGTCGTGGAC
GAPDH	Reverse primer	CCCTCCAGGGGATCTGTTTG
